# Understanding the influence of alkali cations and halogen anions on the cooperativity of cyclic hydrogen‐bonded rosettes in supramolecular stacks

**DOI:** 10.1002/asia.202201010

**Published:** 2022-11-23

**Authors:** Andre Nicolai Petelski, Célia Fonseca Guerra

**Affiliations:** ^1^ Department of Theoretical Chemistry Amsterdam Center for Multiscale Modeling (ACMM) Amsterdam Institute of Molecular and Life Sciences (AIMMS) Vrije Universiteit Amsterdam De Boelelaan 1083 1081 HV Amsterdam The Netherlands; ^2^ Departamento de Ingeniería Química Grupo de Investigación en Química Teórica y Experimental (QuiTEx) Facultad Regional Resistencia Universidad Tecnológica Nacional French 414 H3500CHJ Resistencia Chaco Argentina

**Keywords:** Charge transfer, cooperative effects, hydrogen bonds, self-assembly, stacking interactions

## Abstract

Hydrogen‐bonded supramolecular systems are known to obtain extra stabilization from the complexation with ions, like guanine quadruplex (GQ). They experience strong hydrogen bonds due to cooperative effects. To gain deeper understanding of the interplay between ions and hydrogen‐bonding cooperativity, relativistic dispersion‐corrected density functional theory (DFT‐D) computations were performed on triple‐layer hydrogen‐bonded rosettes of ammeline interacting with alkali metal cations and halides. Our results show that when ions are placed between the stacks, the hydrogen bonds are weakened but, at the same time, the cooperativity is strengthened. This phenomenon can be traced back to the shrinkage of the cavity as the ions pull the monomers closer together and therefore the distance between the monomers becomes smaller. On one hand this results in a larger steric repulsion, but on the other hand, the donor‐acceptor interactions are enhanced due to the larger overlap between the donating and accepting orbitals leading to more charge donation and therefore an enhanced electrostatic attraction.

## Graphical abstract

**Come closer**: DFT‐D computations show that when ions are added into hydrogen‐bonded stacks, the monomers are pushed closer to each other. This inclusion forces stronger orbital interactions and charge transfer against a larger Pauli repulsion. Despite that hydrogen bonds and stacking interactions are weakened, the systems gain a boost of cooperativity.

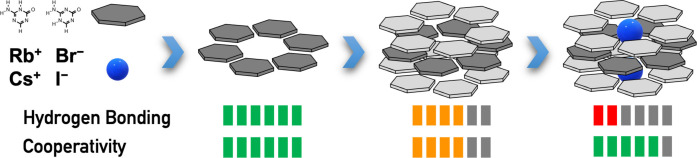

## Introduction

In the context of molecular interactions, cooperativity can be defined as the enhancement of one interaction due to the presence of another one, whether there are three or more molecules interacting each other.[^1^, ^2^] This collective phenomenon is crucial for some macroscopic properties like those of water.^[3]^ Its quantification is therefore of vital importance for a complete understanding of supramolecular assembly.[^2^, ^4^] The study of the impact that ions can exert on cooperative systems can be traced back to 1957 in the work of Frank and Wen about ion‐water interactions.^[5]^ They are one of the first authors that postulated the cooperative nature of water and explained why halide salts promotes negative dielectric relaxation times in water. Years after, the group of Bakker^[6]^ showed how highly hydrated ions induce cooperativity in water. Some theoretical attempts have also been made in this way. While Liu et al.^[7]^ have shown that water clusters (up to 20 molecules) with F^−^ or Li^+^ were found to be negatively cooperative; Guevara‐Vela^[8]^ and coworkers quantified cooperative and anticooperative effects in some water clusters with ions. This lack of a solid consensus indicates there is much work to be done on this topic.

Cooperative systems can then be classified into two main categories: linear chains and cyclic arrangements. The source of cooperativity within the first group has been thoroughly studied within hydrogen,^[9]^ pnicogen^[10]^ and halogen‐bonded systems.^[11]^ However, and to the best of our knowledge, there is only one record that deals with the influence of ions on linear hydrogen‐bonded arrays with positive cooperativity. Subha Mahadevi and Narahari Sastry^[12]^ analyzed linear complexes of water, formamide and acetamide interacting at one of the extremes with Mg^2+^, Na^+^, H^+^, Cl^−^ and OH^−^. They found out that Mg^2+^ promotes the highest bonding energy per monomer added to the cluster. Besides, while monovalent cations have a reduced impact, anions show the lowest synergy improvement among all the systems.

When it comes to cyclic arrangements, it is known that some cooperative systems, like guanine (G) quartets and quadruplexes (GQ), obtain a boost of stabilization when they coordinate cations.^[13]^ This is generally called cation‐templated assembly.[^14^, ^15^] In recent years there has been an increasing attention on hydrogen‐bonded rosettes that are able of coordinating different ions^[16]^ and also transporting them through the central pore when the rosettes stacks on top of each other.^[17]^ Previous studies on GQ pointed out that sodium cations are able to enhance the synergy of the sandwiched G quartet.^[18]^ Yet, it is still unclear the mechanism of this enhancement and whether this is something to be considered in other cyclic systems.

**Scheme 1:**
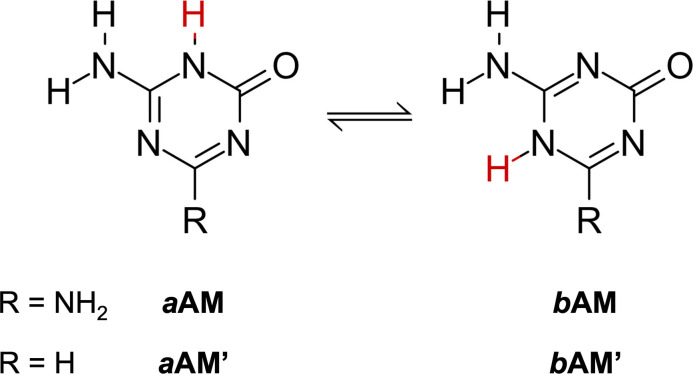
Structure of two tautomers (*a* and *b*) of ammeline ‐AM‐ (R=NH that can form hydrogen bonded rosettes with high synergy (*a*AM and *b*AM). Structures with R=H correspond to our simplified models *a*AM’ and *b*AM’.

With the views above mentioned in mind, in this work we seek to find out the impact of ions on three different cyclic hydrogen‐bonded systems. Based on previous studies,^[19]^ we investigated two hydrogen‐bonded rosettes of two ammeline (AM) tautomers: *a*AM’ and *b*AM’ as shown in Scheme 1. We also considered the G quartet as a natural reference.[^18^, ^20^] By using quantitative Kohn‐Sham molecular orbital (KS‐MO) theory combined with an energy decomposition analyses (EDA) we studied the formation of single rosettes, then the formation of three‐layer systems and finally their coordination with alkali cations (Na^+^, K^+^, Rb^+^ and Cs^+^) and halogen anions (Br^−^ and I^−^). We also followed the cooperativity of the hydrogen bonds in each of the three states and finally uncover why the ions cause an improvement of the synergy.

## Computational Methods

All calculations were performed by using the Amsterdam Density Functional (ADF) program developed by Baerends et al.^[21]^ based on dispersion‐corrected relativistic density functional theory at the ZORA‐BLYP‐D3/TZP level for geometry optimizations and ZORA‐BLYP‐D3/TZ2P for energies. Previous works[^22^, ^23^, ^24^] have shown that this level of theory gives excellent understanding of bonding mechanism in hydrogen bonding and also in long range interactions within weakly‐bound complexes.^[11]^ Furthermore, the use of the TZP basis set for large supramolecular systems has shown to furnish accurate results.^[16c]^

The bonding energies of the dimers, the quartet and the hexamers were computed with Equation 1:

(1) $$\Delta E_{\mathrm{bond}}^{n}=E_{m_{n}}-n\times E_{m};\;n=2,\;4,\;6$$

Here, *n* is the number of monomers and m=*a*AM’, *b*AM’ or G, therefore $E_{m_{n}}$ is the energy of the optimized complex, and *E*_m_ is the energy of the isolated monomer. The overall bond energy of every supramolecular system is also made up of two energy terms, as shown in Equation 2.

(2) $$\Delta E{}_{\mathrm{bond}}=\Delta E{}_{\mathrm{strain}}+\Delta E{}_{\mathrm{int}}$$

Here, the strain energy Δ*E*_strain_ is the energy needed to deform the isolated structures into the geometry they adopt within the complex. The interaction energy Δ*E*_int_ is the actual energy change when the deformed structures are combined to form the interacting complex.^[25]^

The interaction energy can be further decomposed into physically meaningful terms within the framework of the Kohn−Sham molecular orbital theory using a quantitative energy decomposition analysis^[26]^ (EDA). This approach decomposes the Δ*E*_int_ into the following terms [Eq. 3]:

(3) $$\Delta E{}_{\mathrm{int}}=\Delta V{}_{\mathrm{elstat}}+\Delta E{}_{\mathrm{Pauli}}+\Delta E{}_{\mathrm{oi}}+\Delta E{}_{\mathrm{disp}}$$

In this equation, Δ*V*_elstat_ is the classical electrostatic interaction between the unperturbed charge distributions of the prepared units and is usually attractive. The Pauli repulsion Δ*E*_Pauli_ comprises the destabilizing interactions between occupied orbitals and is responsible for steric repulsions. The Δ*E*_oi_ term accounts for donor−acceptor interactions between occupied orbitals on one moiety with unoccupied orbitals of the other, including the HOMO−LUMO interactions) and polarization (empty/occupied orbital mixing on one fragment due to the presence of another fragment). The term Δ*E*_disp_ accounts for the dispersion corrections.

The electron charge redistribution was analyzed with the Voronoi deformation density (VDD) analysis.^[27]^ The atomic charges obtained with this method are computed as the numerical integration of the deformation density in the volume of the Voronoi cell of atom A [Eq. (4)]. This Voronoi cell is determined as the compartment of space between the bond midplanes on and perpendicular to all bond axes between nucleus A and the neighboring nuclei.

(4) $$Q_{A}=-\;\int_{\mathrm{Voronoi}\;\mathrm{cell}\;\mathrm{of}\;A}\left[\rho \left(r\right)-\sum_{B}\rho_{B}\left(r\right)\right]dr$$

In this equation, *ρ*(r) is the electron density of the molecule/supramolecule and Σ_B_ *ρ*_B_(r) is the sum of the atomic densities *ρ*_B_ of a neutral atom without any chemical interaction. The VDD charge *Q*_A_ measures the amount of charge that flows out (*Q*_A_>0) or into (*Q*_A_<0) the Voronoi cell of atom A upon interaction.

## Results and Discussion

### Hydrogen‐bonded rosettes

**Figure 1:**
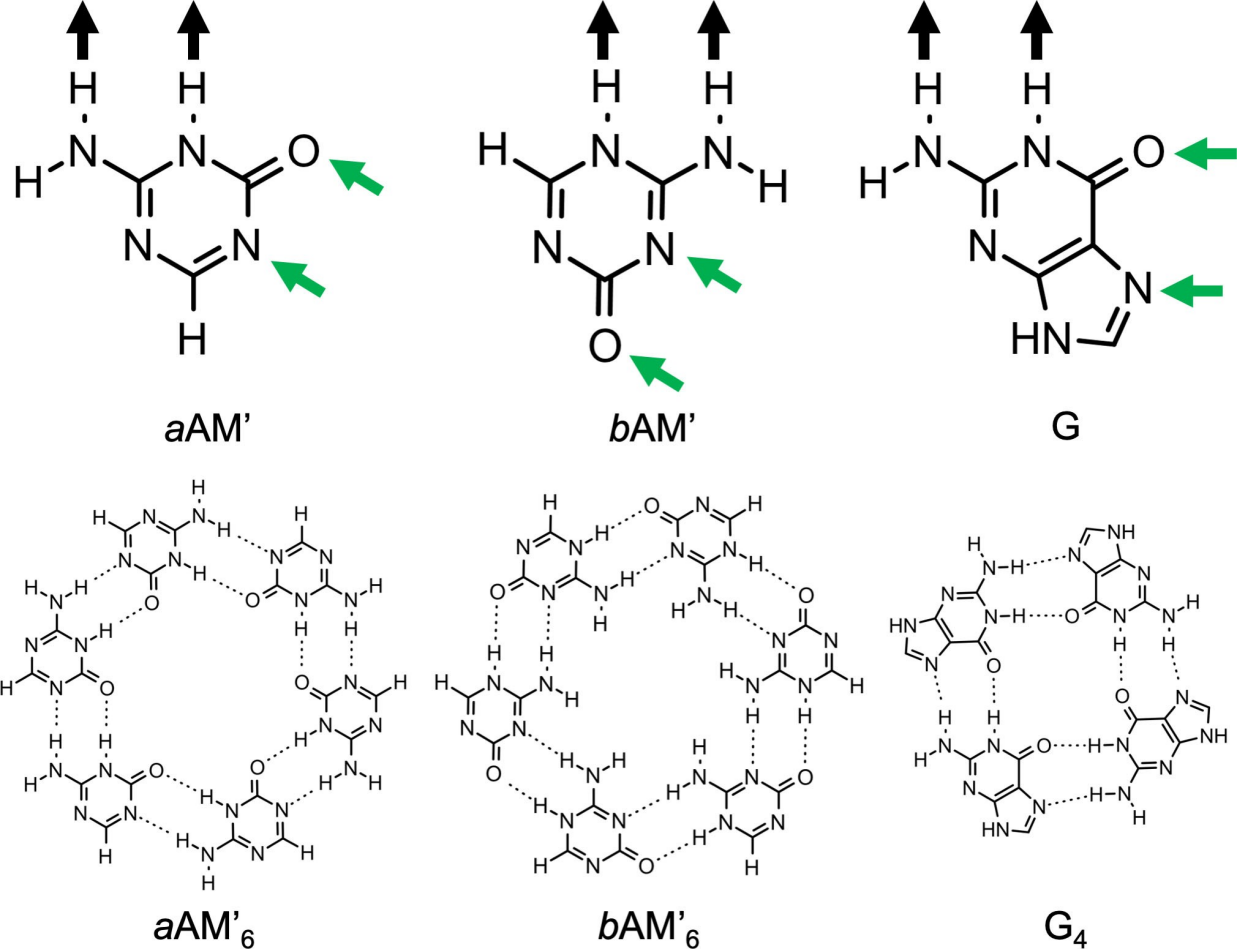
Structures of *a*‐ammeline (*a*AM’), *b*‐ammeline (*b*AM’) and Guanine (G) monomers and their corresponding cyclic hydrogen‐bonded structures. Black and green arrows indicate the hydrogen bond donors (D–H) and acceptors (A) respectively.

We studied two systems that can form hydrogen‐bonded cycles: two tautomers of an AM derivative, *a*AM’ and *b*AM’ as shown in Figure 1. The G quartet was taken as a natural reference (see also Figure 1).[^18^, ^20^] The green arrows indicate the direction of the incoming monomer or hydrogen bonding donor. These systems share the same type of interactions: that is, an N−H⋅⋅⋅N hydrogen bond between an amine group as donor and an endocyclic N atom as acceptor, and an N−H⋅⋅⋅O hydrogen bond between a secondary amine group as donor and a carbonyl group as an acceptor. Therefore, we can compare their hydrogen‐bonding behaviors in different environments.

**Figure 2:**
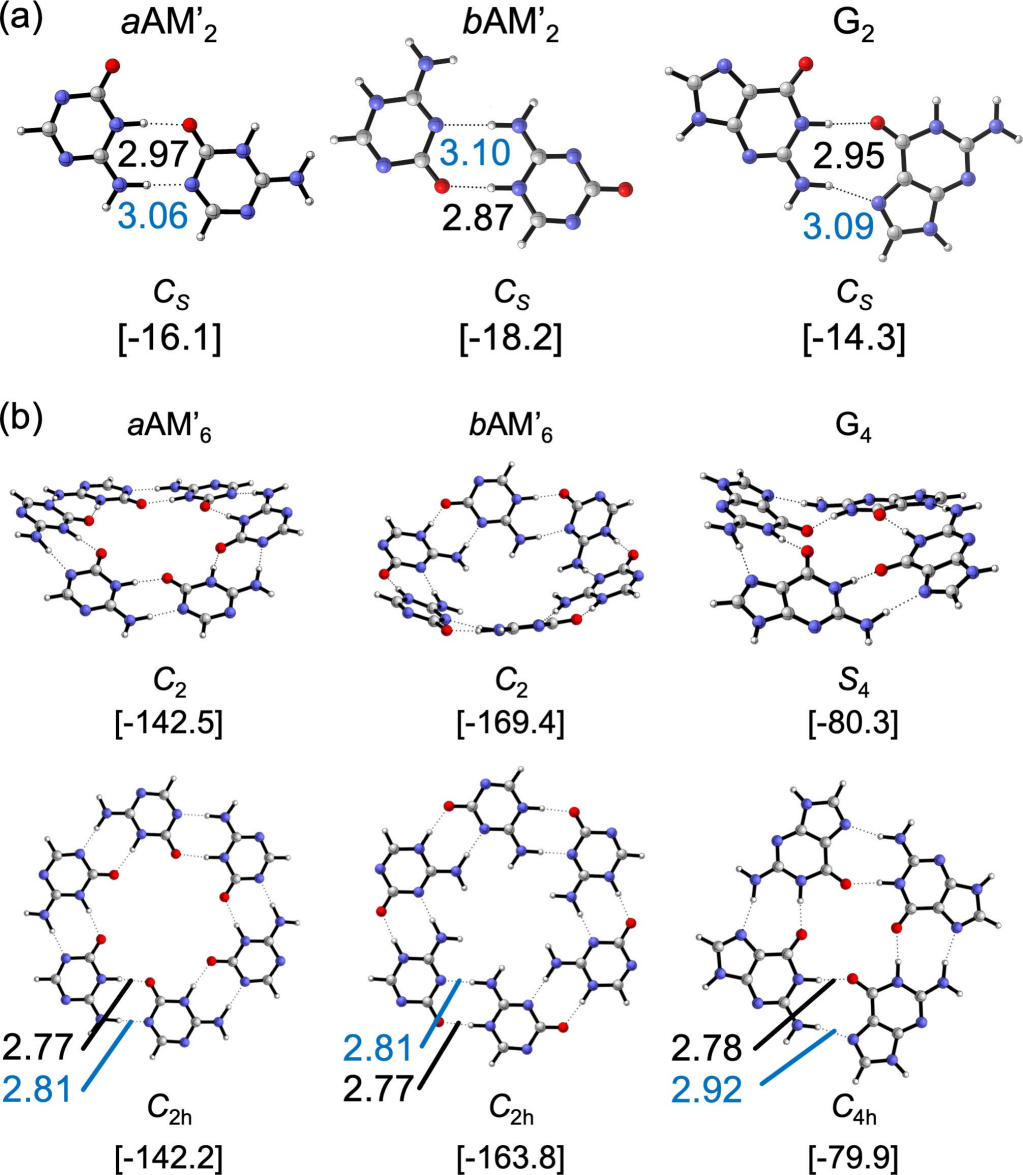
(a) Optimized structures of *a*AM’, *b*AM’ and G hydrogen‐bonded dimers with *C*_S_ symmetry (b) Global minima of rosette‐like structures. Structures were computed at the ZORA‐BLYP‐D3/TZ2P//ZORA‐BLYP‐D3/TZP level of theory. N⋅⋅⋅O bond lengths in black (in Å), N⋅⋅⋅N bond lengths in blue (in Å), and bonding energies between brackets (in kcal mol^−1^).

We started by analyzing the geometries and energies of *a*AM’, *b*AM’ and G dimers, to trace the similarities and differences of their hydrogen bonds. Figure 2.a displays the optimized structures of *a*AM’_2_, *b*AM’_2_ and G_2_ complexes with *C*_S_ symmetry, along with their hydrogen bond distances and energies. By comparing these dimers, the N⋅⋅⋅O bond lengths of the N−H⋅⋅⋅O interactions ranging from 2.87 to 2.95 Å are shorter than the N⋅⋅⋅N distances within the N−H⋅⋅⋅N hydrogen bonds (3.07–3.10 Å), and this is valid for the three systems. The N⋅⋅⋅O bond length of the *b*AM’_2_ dimer is the shortest one among all the dimers. The stabilization energies of the structures are separated by around 2 kcal mol^−1^. The most stabilized dimer is *b*AM’_2_ (−18.2 kcal mol^−1^) followed by *a*AM’_2_ (−16.1 kcal mol^−1^) and G_2_ (−14.3 kcal mol^−1^). To trace the effects of the neighboring monomers in the rosettes and quartets, we will monitor two distances in the next section: The N⋅⋅⋅O (black) and N⋅⋅⋅N (blue) bond lengths of the N−H⋅⋅⋅O and N−H⋅⋅⋅N hydrogen bonds respectively (see Figure 2).

Figure 2b displays the cyclic systems of *a*AM’, *b*AM’ and G. The non‐planar symmetries of *a*AM’_6_ and G_4_ show the same bonding energies as their planar counterparts, ∼−142 and 80 kcal mol^−1^, respectively. However, this is not the case of the *b*AM’_6_ rosette, as it displays a low planarization energy (Δ*E C*_2_ →*C*_2*h*_) of 5.6 kcal mol^−1^. As experimentally these systems are on a surface^[28]^ or stacked on top of each other[^16a^, ^29^] and therefore stabilized by the interaction with the other surface, we will perform the analyses on the planar structures.

**Scheme 2:**
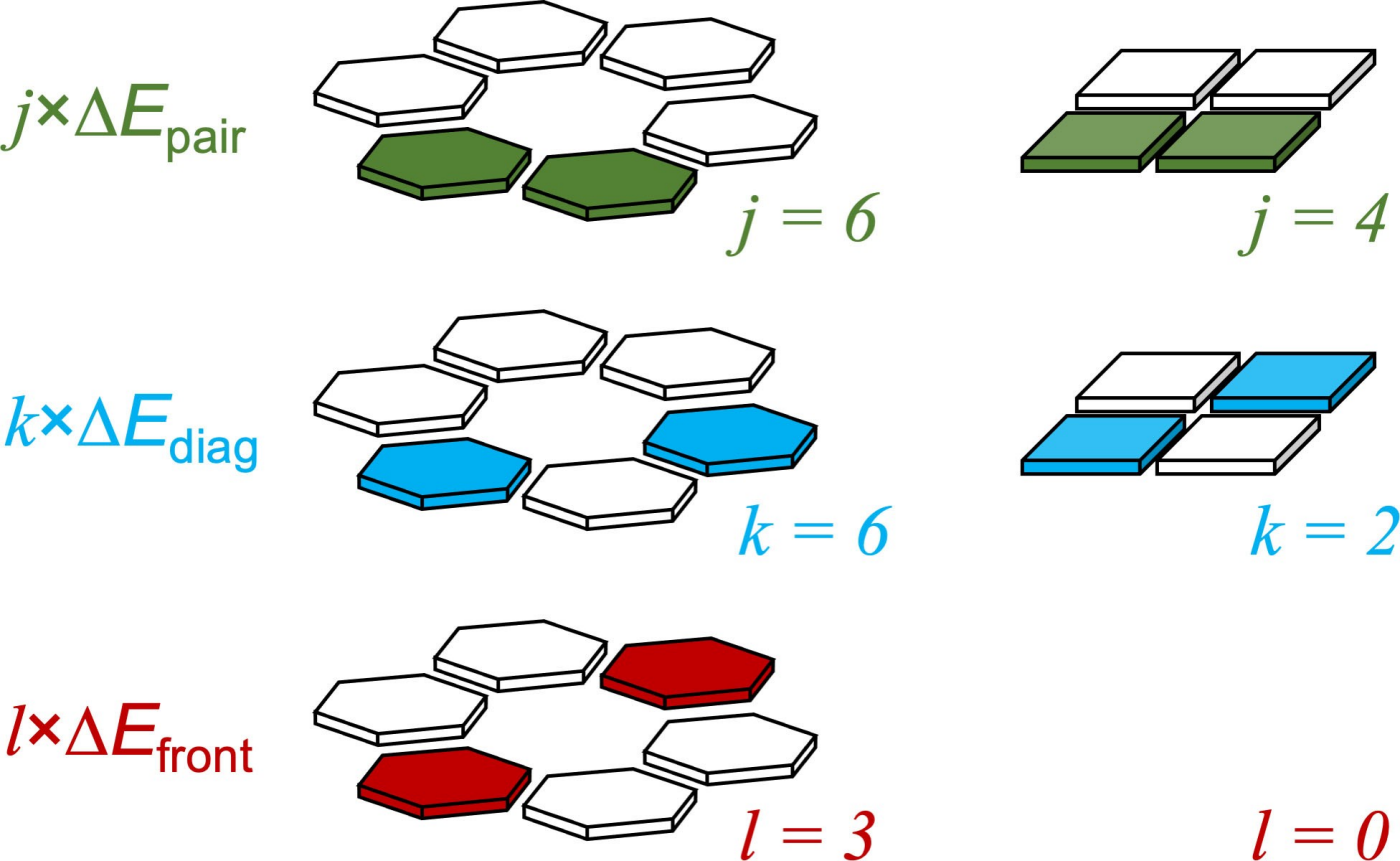
Definition of interaction energy terms in an empty rosette/quartet.

From our works on cooperativity,[^18^, ^19^, ^20^, ^30^] we know that cyclic hydrogen‐bonded systems with the hydrogen bonds pointing in the same direction experience positive cooperativity. The synergy that arises in this type of systems can be computed by comparing the interaction energy of the rosette/quartet Δ*E*_int_ with the summation Δ*E*_sum_ of the individual pairwise interactions for all possible pairs of units in the rosette/quartet [Eq. (5)] as shown in Scheme 2.

(5) $$\Delta E{}_{\mathrm{sum}}=j\times \Delta E{}_{\mathrm{pair}}+k\times \Delta E{}_{\mathrm{diag}}+l\times \Delta E{}_{\mathrm{front}}$$

The interaction energy between two hydrogen‐bonded molecules (see Scheme 2, top) is computed as the difference between the energy of the pair *E*_pair_ and the energy of the corresponding monomers $E_{m}^{\mathrm{pair}}$ with the geometry they acquire in the rosette/quartet [Eq. (6)]. Similarly, the interaction energy between two mutually diagonally oriented molecules Δ*E*_diag_, and the interaction energy between two frontal molecules Δ*E*_front_ is computed according to Equations (7) and (8) respectively.

(6) $$\Delta E_{\mathrm{pair}}=E_{\mathrm{pair}}-2\times E_{m}^{\mathrm{pair}}$$

(7) $$\Delta E_{\mathrm{diag}}=E_{\mathrm{diag}}-2\times E_{m}^{\mathrm{diag}}$$

(8) $$\Delta E_{\mathrm{front}}=E_{\mathrm{front}}-2\times E_{m}^{\mathrm{front}}$$

The synergy Δ*E*_syn_ that occurs in the rosette motifs is then defined as Equation 9

(9) $$\Delta E{}_{\mathrm{syn}}=\Delta E{}_{\mathrm{int}}-\Delta E{}_{\mathrm{sum}}$$

In this formula, if |Δ*E*_int_|>|Δ*E*_sum_| it is said that there is a constructive or positive cooperativity effect, and it means the average energy of the hydrogen bonds is reinforced with regards to their isolated counterparts.

Table 1 collects the bonding energies and hydrogen bond lengths of the dimers and their respective rosettes (*C*_2*h*_ symmetry), and the synergy of their cyclic supramolecules. As can be seen in Table 1, the *d*(N⋅⋅⋅N) and *d*(N⋅⋅⋅O) distances of the hexamers are shorter than those observed in their respective dimers. The *d*(N⋅⋅⋅N) distances are shortened from 3.06 to 2.81 Å within *a*AM’, from 3.10 to 2.81 Å within *b*AM’ and from 3.09 to 2.92 Å within G. With regards to *d*(N⋅⋅⋅O) distances they undergo a shortening of about 0.2 Å when going from dimers to the cycles. In the three cases analyzed herein, the strengthening of the hydrogen‐bonds due to cooperativity effects is also verified. For instance, the dimerization energy of *a*AM’ (the bonding energy of the dimer) is −15.8 kcal mol^−1^, but within the cyclic hexamer the equivalent average pair energy is −23.6 kcal mol^−1^. This result means that the pair of hydrogen bonds within the hexamer becomes 49% stronger relative to the isolated interactions.

**Table 1:** Bond energies [kcal mol^−1^] and hydrogen‐bond lengths [Å] of *a*AM’, *b*AM’ and G dimers and cyclic hydrogen‐bonded complexes with *C*_S_ symmetry.

Complex	Δ*E*_bond_^[a]^	Δ*E*_int_^[b]^	Δ*E*_syn_^[c]^	*d*(N⋅⋅⋅N)^[d]^	*d*(N⋅⋅⋅O)^[d]^
*a*AM’_2_	−16.1	−17.0	–	3.06	2.97
*a*AM’_6_	−142.2	−161.1	−44.6	2.81	2.77
*b*AM’_2_	−18.2	−19.0	–	3.10	2.87
*b*AM’_6_	−163.8	−188.8	−59.8	2.81	2.77
G_2_	−14.3	−16.0	–	3.09	2.95
G_4_	−79.1	−89.0	−20.9	2.92	2.78

[a] Bonding energy [Eq. (1)]. [b] Interaction energy. [c] Synergy [Eqs. (5–9)]. [d] interatomic distances (see Figure 2).

### Structure of 3‐layer systems

**Figure 3:**
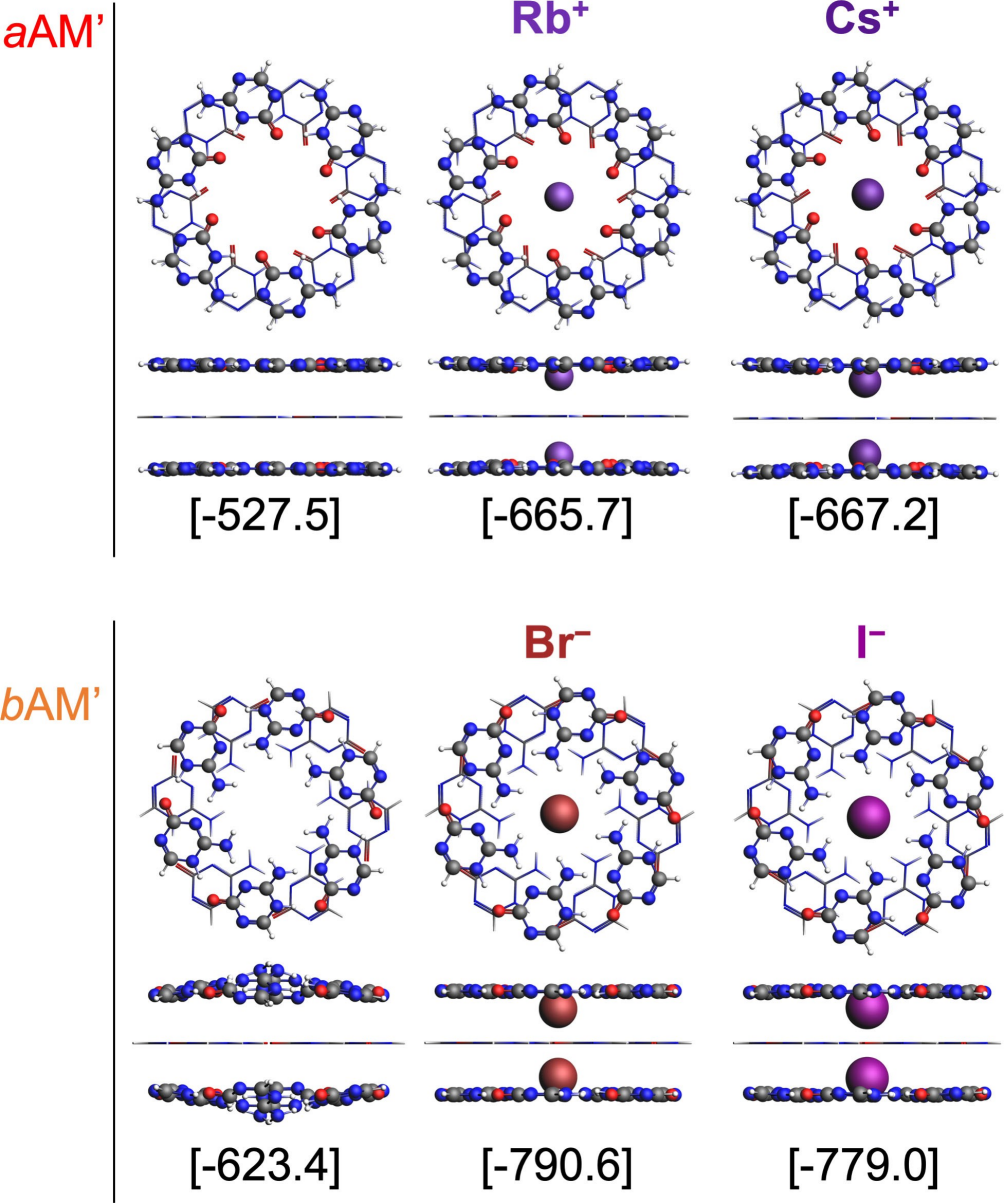
Top and side views of *a*AM’, *b*AM’ three‐layer systems optimized at BLYP‐D3/TZ2P//ZORA‐BLYP‐D3/TZP with *C*_2_ h symmetry in gas phase. Middle rosettes are represented with sticks and outer layers and ions with ball and sticks. Bonding energies are between brackets (in kcal mol^−1^).

In this section we compare the situation of a single rosette between two other layers, this is within a stacked system. Then, alike the GQ, we inserted two ions between the layers. The molecular structures of *a*AM’, *b*AM’ three‐layer systems are shown in Figure 3 (our naturally occurring references, the GQ structures with Na^+^ and K^+^, are shown in Figure S1 in the Supporting Information). The bonding energies displayed in Figure 3 were computed according to Equation 10.

(10) $$\Delta E_{\mathrm{bond}}=E_{m_{6}-i-\left[m_{6}\right]{-i-m}_{6}}-18\cdot E_{m}-2\cdot E_{i}$$

Here $E_{m_{6}-i-\left[m_{6}\right]{-i-m}_{6}}$ is the energy of the stacked system with ions, *E*_m_ is the energy of the isolated monomers (m=*a*AM’ or *b*AM’), and *E*_i_ is the energy of the ion, where i=Rb^+^, Cs^+^, Br^−^ or I^−^. An equivalent formula can be written for the GQ in which the total number of monomers is 12. The optimization of the structures with *C*_2*h*_ symmetry keeps the middle layer planar, and therefore the structures let us compare the cooperativity of the middle rosette under different circumstances. This procedure is only admissible if such structures do not differ too much in energy from those without symmetry restrictions. Therefore, we also optimized the systems with *C*_1_ symmetry (see Figure S2 in the Supporting Information). All the *C*_2*h*_ structures show planar rosettes, except the *b*AM’ system without ions. With regards to the *C*_1_ structures, the *a*AM’ three‐layer system is completely planar, however after ion addition, either Rb^+^ or Cs^+^, all the rosettes adopt a saddle‐like shape. The energy needed to turn these systems planar, this is Δ*E C*_1_ →*C*_2*h*_ is below 1 kcal mol^−1^. The largest difference is that for the *b*AM’ system without ions. The energy needed to turn the *C*_1_ structure into *C*_2*h*_ is just 3 kcal mol^−1^. Thus, the low difference in energy between both symmetries allow us to analyze the *C*_2*h*_ structures. Apart from the *b*AM’ complex, our model complexes are all planar. When looking at our reference, the GQ system evidences some compression on the axis containing the ions (see Figure S1). After introducing Na^+^ or K^+^ into the empty scaffold, the oxygen atoms of the outer G‐quartets are pulled towards the cations, and they adopt a quasi‐bawl‐like shape.

**Figure 4:**
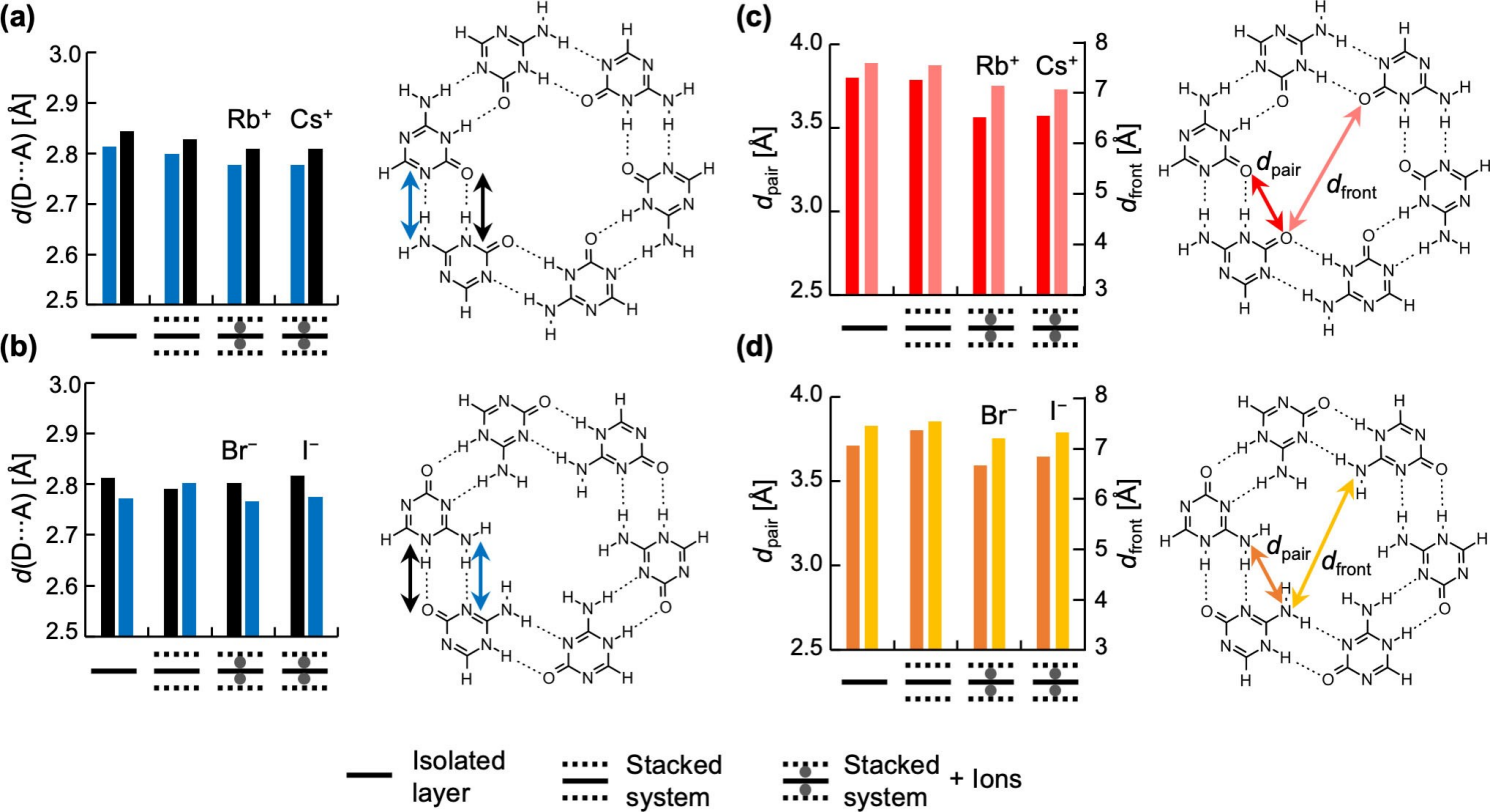
Changes of *d*(D⋅⋅⋅A) (O⋅⋅⋅N and N⋅⋅⋅N) atomic distances of D‐H⋅⋅⋅A hydrogen bonds (D=donor, A=acceptor) within the middle layer of the three‐layer stacks. (a) *a*AM’, **(b)** *b*AM’, and changes of O⋅⋅⋅O and N⋅⋅⋅N atomic distances in (c) *a*AM’, (d) *b*AM’.

To get more structural details, we followed the changes in atomic distances *d*(D⋅⋅⋅A) between heavy atoms of the D−H⋅⋅⋅A hydrogen bonds within the middle rosette, and the average values were plotted in Figure 4a–b. Since we have rosettes with different size cavities and in the presence of alkali cations and halogen anions with also different sizes, there is no clear pattern. Nevertheless, Figure 4 reveals there are two things in common among all systems. First, at least one of the hydrogen bonds of the middle layer undergoes a contraction after introducing ions within the empty scaffolds, which will lead to the modification of the interaction energy. This is also verified when going from the isolated cycle to the 3‐layer system. Second, the elongation or contraction also depends on the size of the ions. Thus, if we look at the middle layers with the series of the smallest ions, this is Rb^+^/Br^−^, the final picture is a rosette more contracted than its isolated counterpart. For example, the *d*(N⋅⋅⋅N) and *d*(N⋅⋅⋅O) distances of the *a*AM’_6_ isolated rosette are 3.03 and 2.97 Å respectively, but, within the Rb^+^ coordination complex the same distances are reduced to 2.78 and 2.81 Å respectively. When analyzing the *b*AM’_6_ hexamer the *d*(N⋅⋅⋅N) and *d*(N⋅⋅⋅O) distances are reduced from 3.10 and 2.88 Å in the empty stack to 2.77 and 2.80 Å within the Br^−^ coordination complex. Due to the smaller size of the GQ cavity, this contraction is more evident (see Figure S3a in the Supporting information). These observations are similar to those of van Mourik and Dingley for GQ.^[17a]^ Even more, previous works on stack systems have demonstrated the resemblance of hydrogen bonds with halogen bonds (XB) within a model system of a brominated G quartet (GBr_4_).^[20]^ Figure S4 shows three parent systems of GQ: GBr_4_ (analogue to G quartet), G_4_‐[GBr_4_]‐G_4_ and G_4_‐K^+^‐[GBr_4_]‐Rb^+^‐G_4_. The middle layer of these stacks displays 6 XB. As shown in Figure S5a, alike our model systems, the G_4_‐K^+^‐[GBr_4_]‐Rb^+^‐G_4_ stack also reveals the halogen‐bonded middle layer is more contracted than its isolated counterpart GBr_4_.

Figures 4c and **d** display the changes of O⋅⋅⋅O and N⋅⋅⋅N atomic distances between hydrogen‐bonded pairs (*d*_pair_) and between frontal molecules (*d*_front_) within the middle layer. This graphic therefore gives information about the radial compression between paired molecules and about the diameter of the inner cavity near the ions. If we move from the isolated rosettes to the empty scaffold and then to the coordination complexes, the changes in the atomic distances of the middle rosette show there is a contraction in all the systems. The analyzed structures show that the systems with the smallest ions (Rb^+^ and Br^−^) are more contracted than their isolated rosettes. When introducing bigger ions (Cs^+^ and I^−^) the atomic distances are slightly expanded again but they do not reach their original values. The same observations are valid for the GQ, as shown in Figure S3b, and also for the halogen‐bonded quartet GBr_4_ as shown in Figure S5b in the Supporting Information. Due to symmetry reasons, the outer layers are completely eclipsed as shown in the top views of Figure 3. Therefore, we also measured the distances between two eclipsed oxygens in the *a*AM’ systems from one outer rosette to the other one, and two eclipsed N(H_2_) atoms in the *b*AM’ system (see Figure S6 in the supporting information). In the *a*AM’_6_‐[*a*AM’_6_]‐*a*AM’_6_→*a*AM’_6_‐Rb^+^‐[*a*AM’_6_]‐Rb^+^‐*a*AM’_6_ transition, there is a contraction of 0,27 Å; in the *b*AM’ system with Br^−^ the interstack N⋅⋅⋅N average distance goes from 7.48 to 6.07 Å, while the G quadruplex with Na^+^ shows a contraction of 0.99 Å with regards to the empty scaffold. These contractions are then translated in lower stacking distances between the layers. Overall, all the ions induce two types of forces in the stacked systems: a radial one that contracts the rosettes and shortens the hydrogen bond distances, and an axial force that reduces the stacking distances between the rosettes.

### Energies of 3‐layer systems

**Scheme 3:**
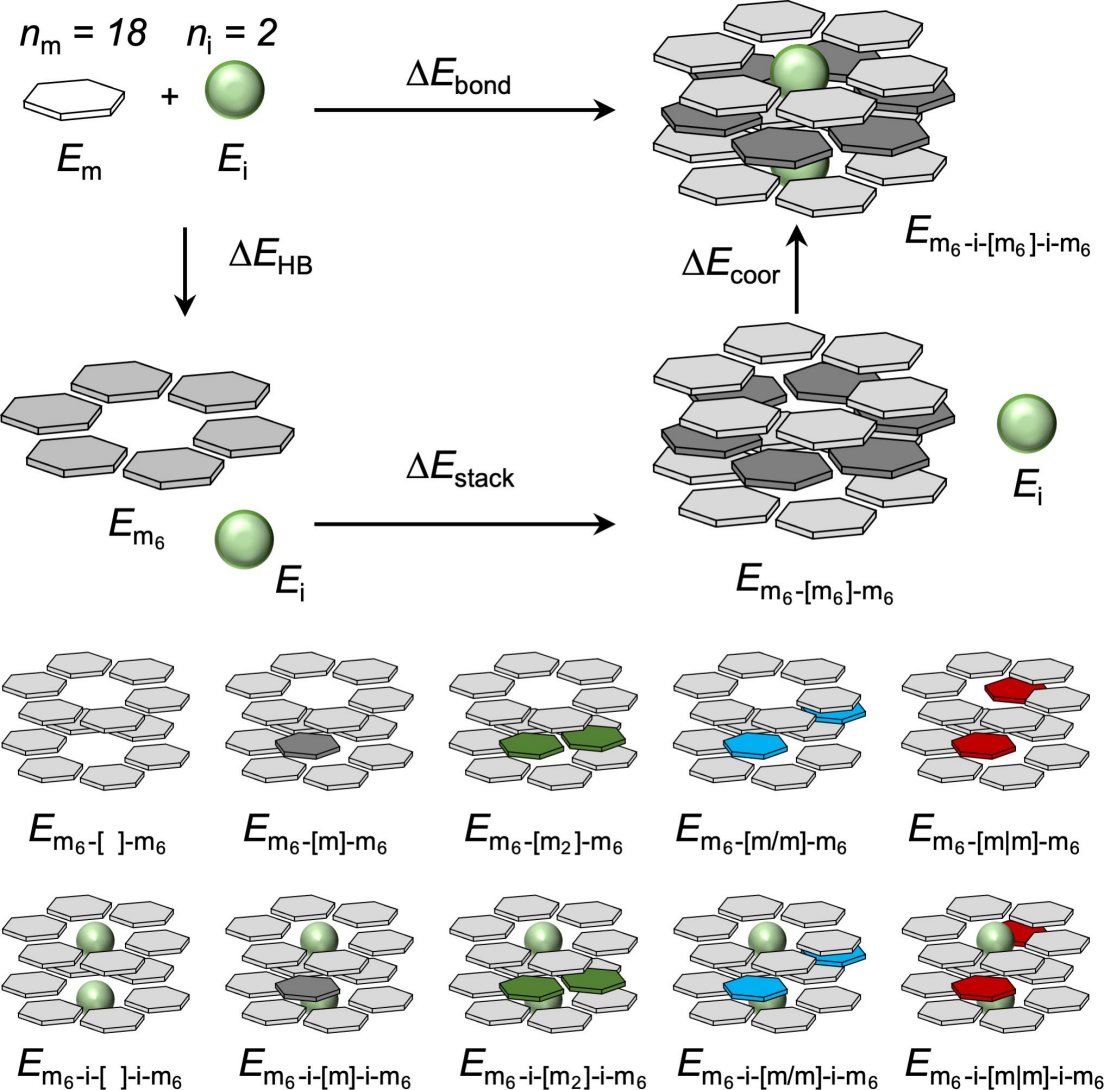
Definition of interaction‐energy terms in the m_6_‐i‐[m_6_]‐i‐m_6_ stacked systems (m=*a*AM’, *b*AM’; i=Rb^+^, Cs^+^, Br^−^ or I^−^).

The bonding energy computed by Equation (10) can be further dissected by following the cycle of Scheme 3. This method let us obtain information about two fundamental energy components: the stacking Δ*E*_stack_ and the coordination Δ*E*_coor_ energies, which were computed with Equations 11 and 12 respectively.

(11) $$\Delta E_{\mathrm{stack}}=E_{m_{6}-\left[m_{6}\right]{-m}_{6}}-E_{m_{6}}^{\mathrm{top}}-E_{m_{6}}^{\mathrm{mid}}-E_{m_{6}}^{\mathrm{bot}}$$

(12) $$\Delta E_{\mathrm{coor}}=E_{m_{6}-i-\left[m_{6}\right]{-i-m}_{6}}-E_{m_{6}-\left[m_{6}\right]{-m}_{6}}-2\cdot E_{i}$$

In Equations (11) and (12), $E_{m_{6}-\left[m_{6}\right]{-m}_{6}}$ is the energy of the prepared stack with the structure of the final coordination complex; and $E_{m_{6}}^{\mathrm{top}}$ , $E_{m_{6}}^{\mathrm{mid}}$ , and $E_{m_{6}}^{\mathrm{bot}}$ are the energies of the prepared rosettes (top, middle and bottom, respectively) with the structures they acquire in the final m_6_‐i‐[m_6_]‐i‐m_6_ complex as shown in Scheme 3.

To inspect the effects of both the presence of the outer layers and the presence of the ions on the hydrogen bonds, we computed the interaction energy of the middle layer [Eq. 13].

(13) $$\Delta E_{\mathrm{int}}=\left(E_{m_{6}-\left[m_{6}\right]{-m}_{6}}-E_{m_{6}-\left[\;\right]{-m}_{6}}\right)-6\cdot \left(E_{m_{6}-\left[m\right]{-m}_{6}}-E_{m_{6}-\left[\;\right]{-m}_{6}}\right)$$

Here, $E_{m_{6}-\left[\;\right]{-m}_{6}}$ is the energy of the outer layers with the structures they acquire in the three‐layer system, and $E_{m_{6}-\left[m\right]{-m}_{6}}$ is the energy of the outer layers but considering just one monomer in between (see also Scheme 3). Since we are interested in the behavior of the middle layer between the stacks, the synergy of the hydrogen‐bonded rosette was quantified by comparing the interaction energy of the middle layer [Eq. (13)] with Δ*E*_sum_ [Eq. (5)], the sum of the individual pairwise interactions for all possible pairs of units within the middle layer. In this new situation Δ*E*_pair_ is the interaction between two hydrogen‐bonded molecules within the middle layer and in the geometry of the rosette but considering the surrounding monomers and ions. Again, Δ*E*_diag_ is the interaction between two mutually diagonally oriented molecules, and Δ*E*_front_ is the interaction between two faced molecules. Each pairwise interaction energy was computed according to Equations (14), (15) and (16). All the interaction energy terms are defined in Scheme 3. Equivalent formulas can be written for the systems with ions but considering the energy terms shown at the bottom of Scheme 3 (see Equations (S1‐S3) in the supporting information).

(14) $$\Delta E_{\mathrm{pair}}=\left(E_{m_{6}-\left[m_{2}\right]{-m}_{6}}-E_{m_{6}-\left[\;\right]{-m}_{6}}\right)-\;2\cdot \left(E_{m_{6}-\left[m\right]{-m}_{6}}-E_{m_{6}-\left[\;\right]{-m}_{6}}\right)$$

(15) $$\Delta E_{\mathrm{diag}}=\left(E_{m_{6}-\left[m/m\right]{-m}_{6}}-E_{m_{6}-\left[\;\right]{-m}_{6}}\right)-\;2\cdot \left(E_{m_{6}-\left[m\right]{-m}_{6}}-E_{m_{6}-\left[\;\right]{-m}_{6}}\right)$$

(16) $$\Delta E_{\mathrm{front}}=\left(E_{m_{6}-\left[\left.m\right|m\right]{-m}_{6}}-E_{m_{6}-\left[\;\right]{-m}_{6}}\right)-\;2\cdot \left(E_{m_{6}-\left[m\right]{-m}_{6}}-E_{m_{6}-\left[\;\right]{-m}_{6}}\right)$$

As shown in Table 2 for *a*AM’ and *b*AM’ systems (see also Table S1 for GQ), the ions weaken the hydrogen bond energy of the middle rosette. In the case of *a*AM’ and *b*AM’, the metal ions reduce the hydrogen bond energy by 8% (from −156.5 to −143.4 kcal mol^−1^), whilst the halogen anions also reduce this energy by around 3% (from −184.1 to −177.6 kcal mol^−1^). With regards to the G quadruplex, the bigger the ion, the smaller the weakening: sodium destabilizes the hydrogen bond energy by 34%, while potassium does it by 18%. The fact that the structural changes are more pronounced in the GQ is consistent with the weakening of the hydrogen bonds. Likewise, in all cases, the stacking energy is also destabilized after ions addition. This is because within the coordination complexes the rosette layers are closer than in the empty systems, as shown in Figure S4. Consequently, Pauli repulsion increases as the orbitals get closer to each other (see Table S2) and the stacking energy becomes less stabilizing.

**Table 2:** Table Analysis of interaction energies [kcal mol^−1^] of AM stack complexes.

Top layer	[ ]	Bottom layer	[Middle layer]	Δ*E*_int_^[a]^	Δ*E*_sum_^[b]^	Δ*E*_syn_^[c]^	Δ*E*_stack_^[d]^	Δ*E*_coor_^[e]^
*a*AM’_6_‐	[ ]	‐*a*AM’_6_	*a*AM’_6_	−156.5	−120.6	−35.9	−93.5	–
*b*AM’_6_‐	[ ]	‐*b*AM’_6_	*b*AM’_6_	−184.1	−139.4	−44.7	−121.8	–
*a*AM’_6_‐Rb^+^‐	[ ]	‐Rb^+^‐*a*AM’_6_	*a*AM’_6_	−143.4	−104.4	−39.0	−84.8	−239.9
*a*AM’_6_‐Cs^+^‐	[ ]	‐Cs^+^‐*a*AM’_6_	*a*AM’_6_	−144.9	−106.0	−38.9	−86.7	−235.8
*b*AM’_6_‐Br^−^‐	[ ]	‐Br^−^‐*b*AM’_6_	*b*AM’_6_	−177.6	−129.3	−48.3	−109.9	−269.3
*b*AM’_6_‐I^−^‐	[ ]	‐I^−^‐*b*AM’_6_	*b*AM’_6_	−178.6	−131.9	−46.7	−112.9	−245.8

[a] Interaction energy of the middle layer [Eq. (13)]. [b] Summation of the individual pairwise interactions [Eq. (5, 14–16)]. [c] Synergy of the middle layer [Eq. (9)]. [d] Stacking energy [Eq. (11)]. [e] Coordination energy [Eq. (12)].

When looking the *a*AM’ systems, Rb^+^ and Cs^+^ cause an improvement of the synergy from −36 to −39 kcal mol^−1^. Besides, bromide cause a maximum increment of the synergy by 3.6 kcal mol^−1^. As was already pointed out in previous work,^[18]^ the sodium cations preserve the cooperativity of the middle layer of G_4_ even to a higher extent than in the stacks without them. Thus, the improvement of the synergy due to ion addition is also present in other cyclic hydrogen‐bonded systems like those observed in this work. The intuitive concept of cooperativity states that the average hydrogen bond energy is strengthened. Therefore, one may wonder how the synergy is enhanced while the hydrogen bond energy is weakened at the same time.

### The origin of the improved synergy

**Scheme 4:**
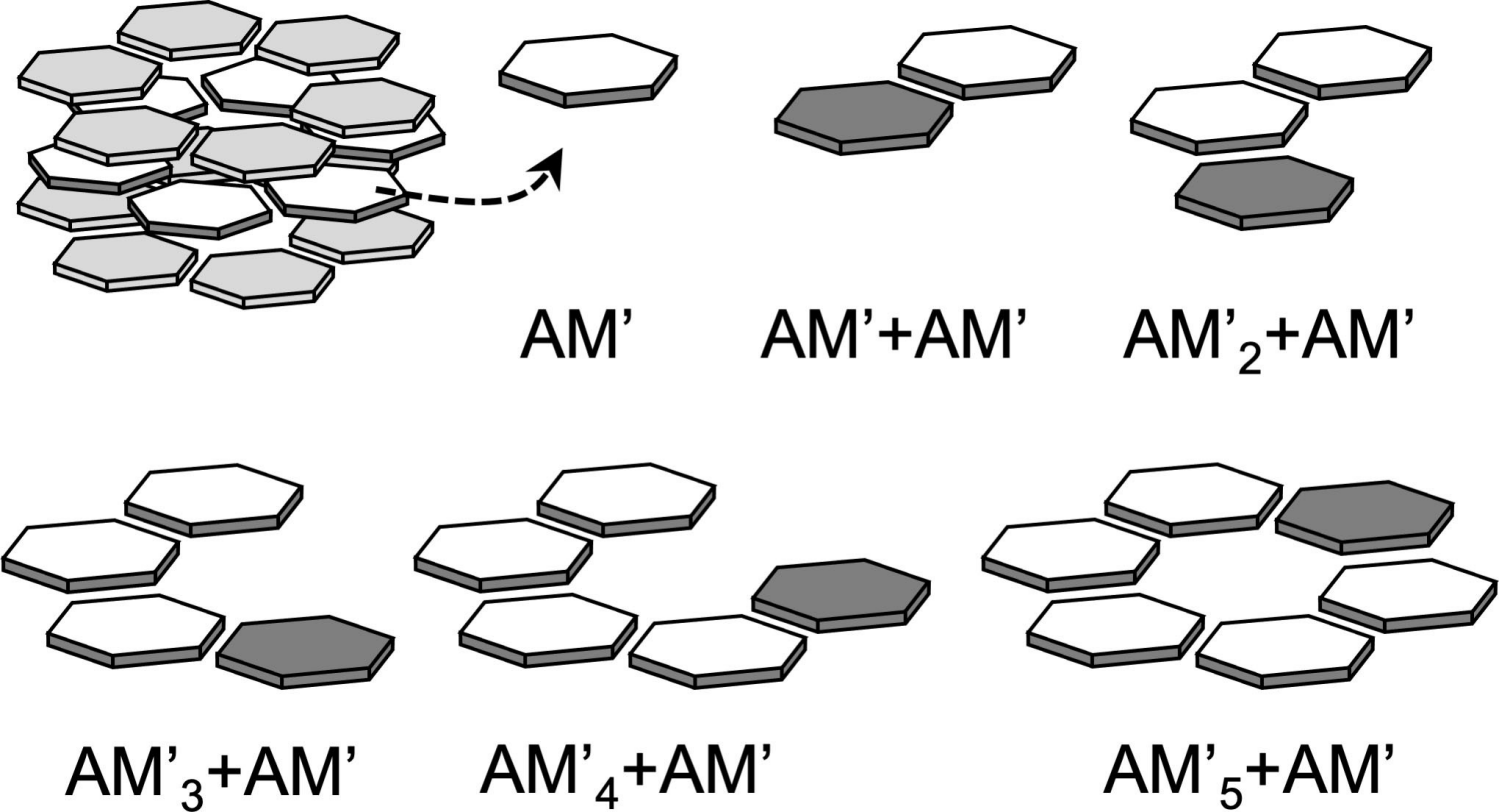
Representation of molecular fragments in the formation of the cyclic structures by a stepwise addition of monomers (*m*=*a*AM’, *b*AM’) in one‐way direction: *m*_n_+*m* (n=1, 2, 3, 4, 5). Dark grey hexagons represent the incoming *m* (electron donors).

Now that we know how the monovalent ions modify the supramolecular structures, we will focus on the synergy of the central layer. To this end, we took the middle layers out of their environments and analyzed them within two situations: first, in the system without ions, and second, in the system that undergoes the biggest contraction, this is those with Rb^+^ and Br^−^. We explored the development of the synergy by analyzing the construction of the hydrogen‐bonded cycles monomer by monomer. Scheme 4 shows this procedure as a stepwise addition of an incoming monomer (grey hexagon) to the former one till the close of the cycle. The white hexagons were considered as a single fragment. Then, we decomposed the interaction energy in each step. The synergy of the EDA components (either interaction, orbital, Pauli, electrostatic or dispersion) within the hexamers, is computed as follow:

(17) $$\Delta E_{\mathrm{syn},\;\mathrm{oi}}\;=\sum_{n=1}^{5}\Delta E_{\mathrm{oi}}\left(m_{n+1}\right)--\Delta E_{\mathrm{sum},\;\mathrm{oi}}$$

Here, Δ*E*_sum, oi_ is the Δ*E*_sum_ computed by Equation (5) but for the orbital interaction component. The Δ*E*_syn_ of the other EDA components was computed as follows:

(18) $$\Delta E_{\mathrm{syn},\;\mathrm{Pauli}}\;=\sum_{n=1}^{5}\Delta E_{\mathrm{Pauli}}\left(m_{n+1}\right)\;--\Delta E_{\mathrm{sum},\;\mathrm{Pauli}}$$

(19) $$\Delta V_{\mathrm{syn},\;\mathrm{elstat}}\;=\sum_{n=1}^{5}\Delta V_{\mathrm{elstat}}\left(m_{n+1}\right)-\Delta V_{\mathrm{sum},\;\mathrm{elstat}}$$

(20) $$\Delta E_{\mathrm{syn},\;\mathrm{disp}}\;=\sum_{n=1}^{5}\Delta E_{\mathrm{disp}}\left(m_{n+1}\right)--\Delta E_{\mathrm{sum},\;\mathrm{disp}}$$

Results for *a*AM’_6_ and *b*AM’_6_ are collected in Tables 3 and 4 respectively, while results of our reference system (GQ) are shown in Table S3. In each table we have two situations: before (a) and after ion addition (b). From Tables 3.a and 4.a we can see the increase of every component with the regular addition of monomers due to the presence of cooperativity, as was already observed in previous works within the same isolated systems.[^18^, ^20^] In all the cases, the greatest contribution to the synergy comes mainly from the electrostatic and orbital interactions.

**Table 3:** Energy decomposition [kcal mol^−1^] for the formation of the middle rosette [*a*AM_*n*+1_] from *a*AM_*n*_+*a*AM in a stepwise one‐way direction (*n*=1, 2, 3,4,5).

*n*+1	Δ*E*_int_	Δ*E*_oi_	Δ*E*_Pauli_	Δ*V*_elstat_	Δ*E*_disp_
**(a)** *a*AM_6_−[*a*AM_*n*+1_]−*a*AM_6_					
1+1	−17.0	−20.5	39.9	−32.1	−4.4
2+1	−23.8	−22.8	39.1	−35.7	−4.4
3+1	−26.8	−23.7	38.9	−37.5	−4.4
4+1	−30.6	−24.7	38.6	−40.0	−4.5
5+1	−63.3	−56.0	80.2	−78.6	−8.9
Δ*E*_syn_	−47.1	−24.1	−3.4	−19.5	0.0
**(b)** *a*AM_6_‐Rb^+^‐[*a*AM_*n*+1_]‐Rb^+^‐*a*AM_6_					
1+1	−16.2	−21.8	43.0	−32.7	−4.7
2+1	−22.7	−23.8	41.2	−35.4	−4.8
3+1	−26.5	−25.7	42.2	−38.4	−4.7
4+1	−30.1	−26.4	41.5	−40.3	−4.9
5+1	−62.7	−59.7	85.9	−79.5	−9.5
Δ*E*_syn_	−50.2	−25.8	−4.1	−20.4	0.0

**Table 4:** Energy decomposition [kcal mol^−1^] for the formation of the middle rosette [*b*AM_*n*+1_] from *b*AM_*n*_+*b*AM in a stepwise one‐way direction (*n*=1, 2, 3,4,5).

*n*+1	Δ*E*_int_	Δ*E*_oi_	Δ*E*_Pauli_	Δ*V*_elstat_	Δ*E*_disp_
**(a)** *b*AM_6_−[*b*AM_*n*+1_]−*b*AM_6_					
1+1	−20.1	−22.1	41.8	−34.8	−5.0
2+1	−27.9	−25.7	44.5	−41.7	−5.1
3+1	−31.4	−24.4	40.2	−42.2	−4.9
4+1	−35.8	−27.3	43.3	−46.7	−5.1
5+1	−75.5	−61.6	82.5	−86.3	−10.0
Δ*E*_syn_	−57.9	−27.8	1.2	−31.3	0.1
**(b)** *b*AM_6_‐Br^−^‐[*b*AM_*n*+1_]‐Br^−^‐*b*AM_6_					
1+1	−19.5	−25.0	48.0	−37.2	−5.3
2+1	−27.8	−27.9	46.5	−41.1	−5.4
3+1	−31.6	−29.3	46.3	−43.2	−5.4
4+1	−36.4	−30.8	46.1	−46.2	−5.5
5+1	−77.4	−70.1	95.7	−92.3	−10.8
Δ*E*_syn_	−64.4	−32.3	−5.3	−26.7	0.0

We have seen that after ion addition to the cavities of the three‐layer systems, the middle layer rosettes undergo a radial contraction. In this transition the synergy is enhanced. The improvement of the synergy is also present in these isolated layers. However, the values are not the same as those of Table 2 because we are losing the effects of the neighboring monomers. The extra gain in cooperativity is −3.1, −6.5 and −1.9 kcal mol^−1^, respectively, for the *a*AM’_6_, *b*AM’_6_ and G_4_ layers. As shown in Tables 3 and 4 and Table S3 the sequential pair interactions within situation (b) are weaker than those of (a). The first dimerization energy decreases as follow: from −17.0 to −16.2 kcal mol^−1^ for *a*AM’_2_, from −20.1 to −19.5 kcal mol^−1^ for *b*AM’_2_, and from −16.0 to −14.2 kcal mol^−1^ for G_2_. However, due to the shorter hydrogen bond distances, the orbital interactions in the situation **(b)** are stronger than those in **(a)** and they become even stronger with the incoming monomer. As expected, the Pauli repulsion, which accounts for the repulsive interaction between the occupied orbitals of the two moieties, also increases after the contraction of the hydrogen bonds.

**Figure 5:**
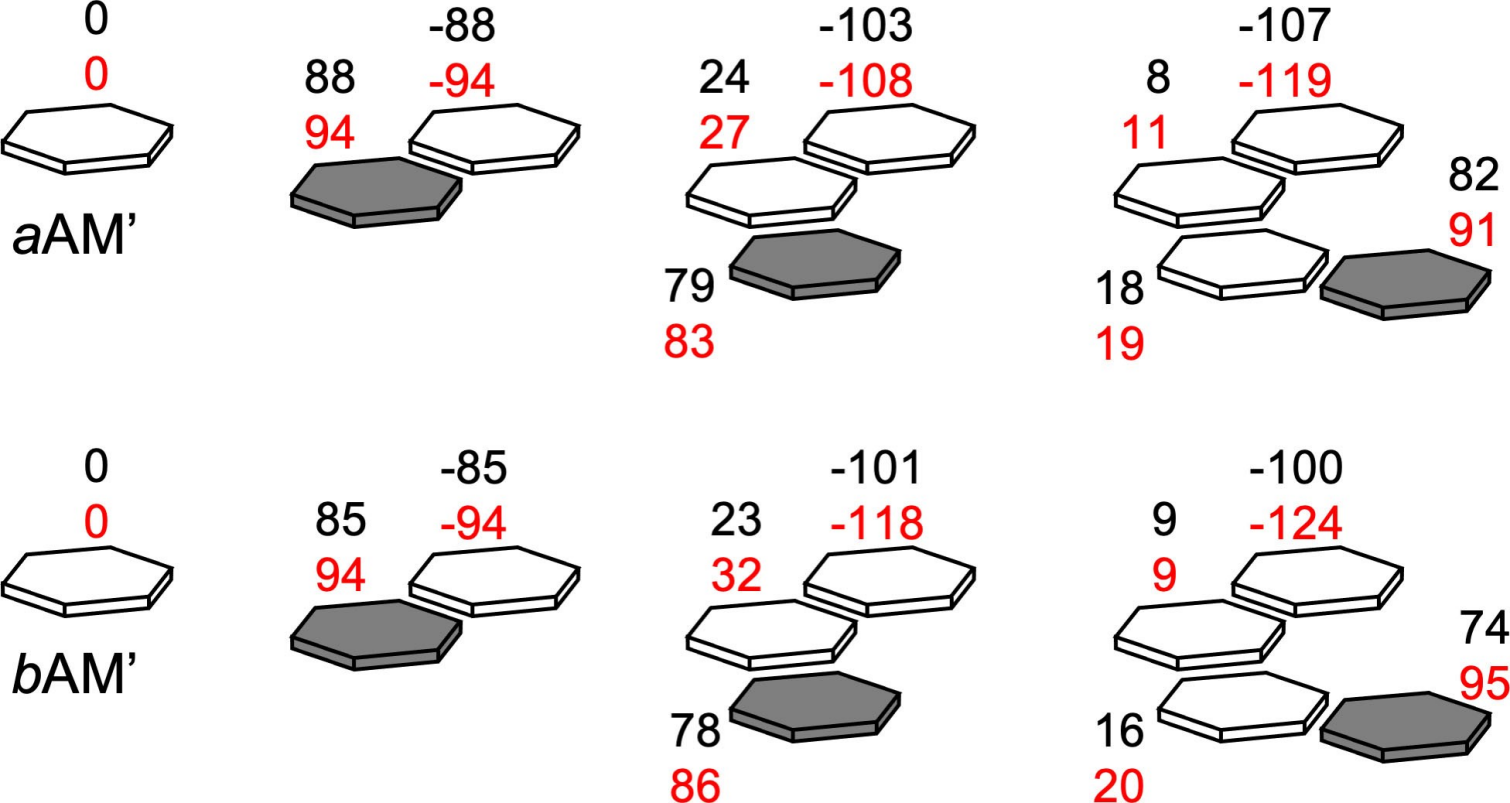
VDD charges in milli‐electron units before (black values) and after (red values) ion addition (Rb^+^ for *a*AM’ and Br^−^ for *b*AM’) in the formation of cyclic structures.

We can get more insight into the improvement of cooperativity by analyzing the charge redistribution before and after the contraction. To this end, we computed the Voronoi deformation density (VDD) charges by following the steps of Scheme 4. In line with our previous reports,^[20]^ the charge separation gradually increases throughout the stepwise addition of monomers as shown in Figure 5 (black values).

**Figure 6:**
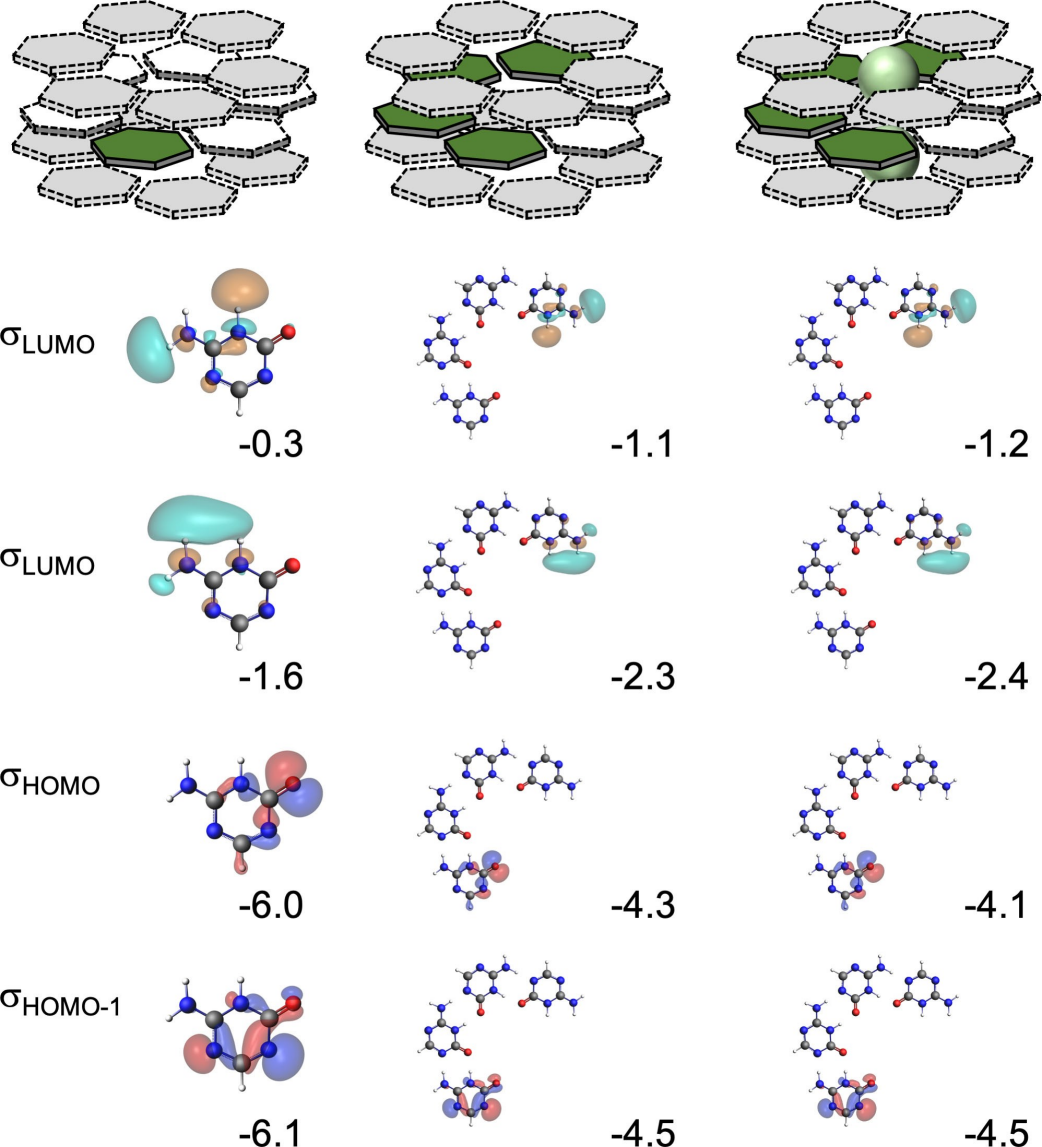
N−H unoccupied orbitals (σ_LUMO_ and σ_LUMO+1_), and oxygen (σ_HOMO_) and nitrogen lone‐pair orbitals (σ_HOMO−1_), of the front atoms of *a*AM’ and *a*AM’_4_ and their corresponding energies [eV] for three different states (top). Calculated at the ZORA‐BLYP‐D3(BJ)/TZ2P level of theory.

For instance, the monomers with available hydrogen bond acceptors (white squares and hexagons) become more negative in each step. The first *a*AM’ and *b*AM’ monomers experience a gain of −19 (from −88 to −107 milli‐electrons) and −15 milli‐electrons (from −85 to −100 milli‐electrons) respectively (see black values of Figure 5). After the addition of the first series of ions (Rb^+^ and Br^−^), the middle layers undergo a contraction that reduces some of the hydrogen bond distances as was shown in Figure 4. Therefore, the incoming monomers push more charge into the sigma electron system due to stronger orbital donor‐acceptor interactions in these contracted systems. Red values of Figure 5 show the extra gain of charge because of the contraction. The *a*AM’ dimer has VDD atomic charges of −88 and +88 milli‐electrons before ion addition. Within the second situation, the charge separation rises to −94 and +94 milli‐electrons. As shown in Figure 6, the antibonding LUMOs of the hydrogen‐bond donor are stabilized because they get more positive (see Figure 5), while lone pair orbitals are destabilized due to a charge accumulation. Therefore, the donor‐acceptor interactions are more stabilizing within the contracted systems. The same effect is also observed in the *b*AM’ and G systems. Therefore, the radial contraction forces more charge‐transfer within the sigma electron system due to stronger donor‐acceptor orbital interactions between the monomers. The electrostatic attraction becomes more pronounced, and this effect results in an extra gain of cooperativity.

## Conclusion

In this work we have uncovered how ions can improve cooperativity in hydrogen and halogen‐bonded supramolecular stacks. We have thoroughly analyzed the cooperativity effects within three cyclic hydrogen‐bonded supramolecules and how ion coordination influences the structure and the synergy within the layers of the supramolecular stacks.

Our DFT‐D computations reveal that the three‐layer systems undergo subtle structural changes when they accommodate ions within their cavities. These geometrical rearrangements include hydrogen bonds shortening, the reduction of the rosette's inner diameter and the simultaneous flattening of the stacks, which is translated in a shrinkage of the cavity. This is also valid for halogen bonded cyclic rosettes. These modifications are produced by a compression that the ions exert due to coordination. The smaller the ion the bigger the compression of the cavity.

Since structural changes are associated to stability, after ion introduction the hydrogen bond energy of the middle rosette is weakened, but at the same time the synergy is improved. The compression between the monomers imposes stronger orbital donor‐acceptor interactions due to the shorter intermolecular distances and thus more charge transfer between the two monomers. At the same time, this effect is also counteracted by larger Pauli repulsion due to the larger overlap between the occupied orbitals. The overall result is an enhancement of cooperativity despite the debilitation of non‐covalent interactions.

## Conflict of interest

The authors declare no conflict of interest.

1

## Supporting information

As a service to our authors and readers, this journal provides supporting information supplied by the authors. Such materials are peer reviewed and may be re‐organized for online delivery, but are not copy‐edited or typeset. Technical support issues arising from supporting information (other than missing files) should be addressed to the authors.

Supporting Information

## Data Availability

The data that support the findings of this study are available in the supplementary material of this article.
