# Supplementary material for: Understanding the influence of alkali cations and halogen anions on the cooperativity of cyclic hydrogen‐bonded rosettes in supramolecular stacks
Source: Chem Asian J. 2022 Nov 23;17(24):e202201010. doi: 10.1002/asia.202201010 (PMC10099620; doi:10.1002/asia.202201010)
Supplement: Supplementary file 1 — Supporting Information [file ASIA-17-0-s001.pdf]

CHEMISTRY
AN ASIAN JOURNAL

Supporting Information

Understanding the influence of alkali cations and halogen anions on the cooperativity of cyclic hydrogen-bonded rosettes in supramolecular stacks

Andre Nicolai Petelski* and Célia Fonseca Guerra*This manuscript is part of a special collection on Halogen Bonding.© 2022 Wiley-VCH GmbH. This is an open access article under the terms of the Creative Commons Attribution License, which permits use, distribution and reproduction in any medium, provided the original work is properly cited.

Figure S1. Top and side views of guanine quadruplexes optimized at BLYP-D3/TZ2P//ZORA-BLYP-D3/TZP with C_{2h} symmetry in gas phase. Middle rosettes are represented with sticks and outer layers and ions with ball and sticks. Bonding energies are between brackets (in kcal mol^{-1})…………………S2

Figure S2. Top and side views of *a*AM', *b*AM' three-layer systems optimized at BLYP-D3/TZP without symmetry restrictions (C_1) in gas phase. Middle rosettes are represented with sticks and outer layers and ions with ball and sticks…………………………………………………………………S2

Figure S3. (a) Changes of *d*(D···A) (O···N and N···N) atomic distances within D–H···A hydrogen bonds (D = donor, A = acceptor) within GQ middle layer, and **(b)** changes of O···O atomic distances within GQ middle layer……………………………………………………………………………………S3

Figure S4. (a) Structures of brominated guanine quadruplex (GBr_4) and two sandwich model complexes of GBr_4 in between two guanine quartets optimized at BLYP-D3(BJ)/TZ2P (taken from L.P. Wolters, N. W. G. Smits, C. Fonseca Guerra, *Phys. Chem. Chem. Phys.* **2015**, *17*, 1585-1592)………S3

Figure S5. (a) Changes of *d*(Br···A) (Br···O and Br···N) atomic distances within N–Br···A halogen bonds (D = donor, A = acceptor) within the Br-halo-guanine quartet middle layer, and **(b)** changes of O···O atomic distances within GQ middle layer………………………………………………………S3

Figure S6. Average O···O and N···N distances from top to bottom layer………………………………S4

Supporting equations. Individual pairwise interactions for all possible pairs of units in the stack complexes with ions, where m_6 is either *a*AM'$_6$ or *b*AM'$_6$, and i = Rb+, Cs+, Br$^-$, I$^-$…………………..S4

Table S1. Analysis of interaction energies [kcal mol^{-1}] of guanine quadruplex………………………S5

Table S2. Energy decomposition analysis [kcal mol^{-1}] of the stacking energy (interaction energy between the layers) computed at BLYP-D3/TZP//BLYP-D3/TZ2P…………………………………..S5

Table S3. Energy decomposition [kcal mol^{-1}] for the formation of the middle quartet [G_{n+1}] from G_n+G in a stepwise one-way direction (n = 1, 2, 3) computed at BLYP-D3/TZP//BLYP-D3/TZ2P………..S5

Cartesian coordinates……………………………………………………………………………..S6

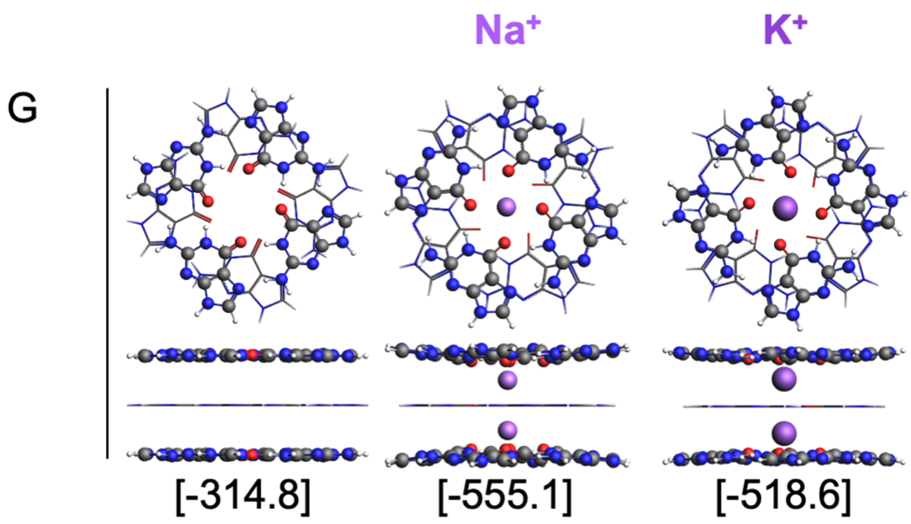

Figure S1. Top and side views of guanine quadruplexes optimized at BLYP-D3/TZ2P//ZORA-BLYP-D3/TZP with C_{2h} symmetry in gas phase. Middle rosettes are represented with sticks and outer layers and ions with ball and sticks. Bonding energies are between brackets (in kcal mol^{-1}).

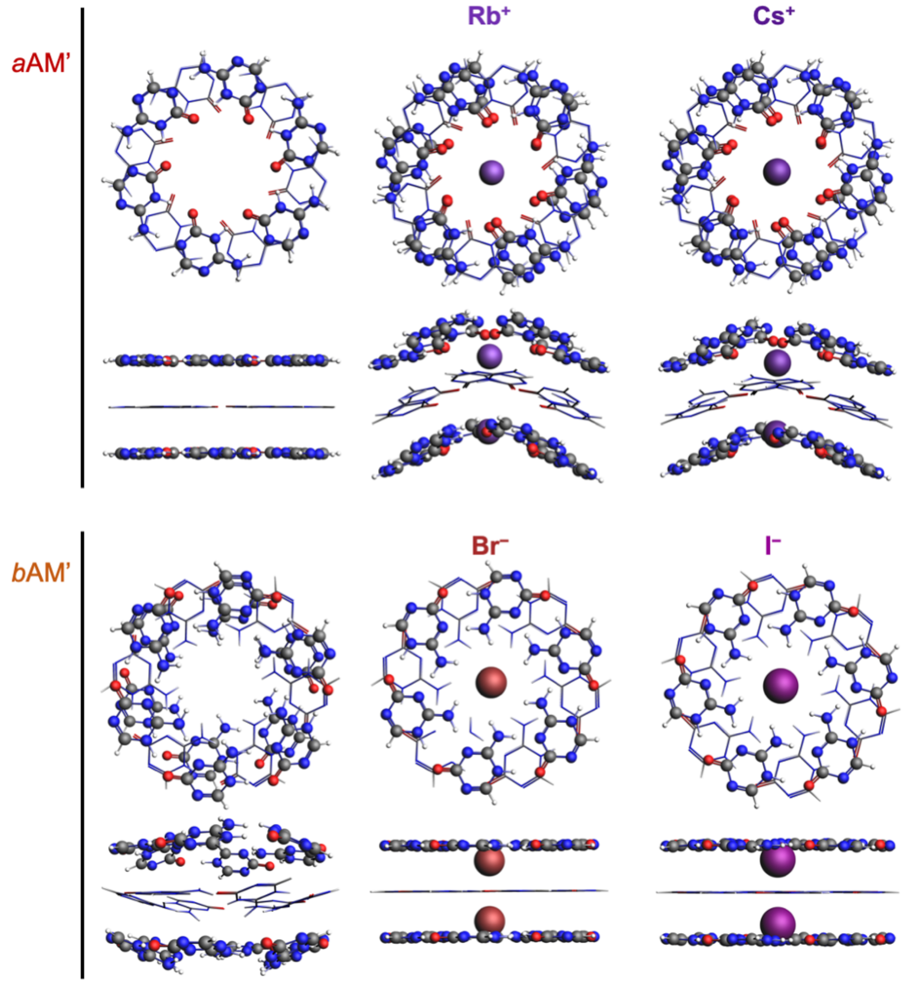

Figure S2. Top and side views of *a*AM', *b*AM' three-layer systems optimized at BLYP-D3/TZP without symmetry restrictions (C_1) in gas phase. Middle rosettes are represented with sticks and outer layers and ions with ball and sticks.

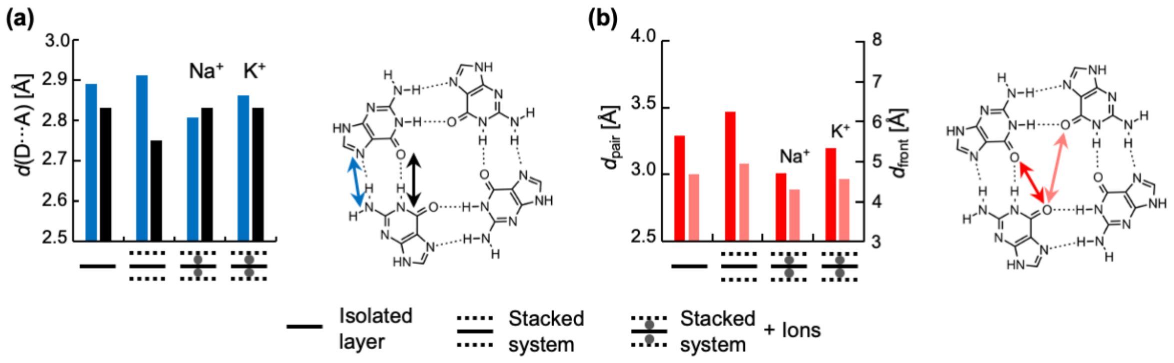

Figure S3. (a) Changes of *d*(D···A) (O···N and N···N) atomic distances within D–H···A hydrogen bonds (D = donor, A = acceptor) within GQ middle layer, and **(b)** changes of O···O atomic distances within GQ middle layer.

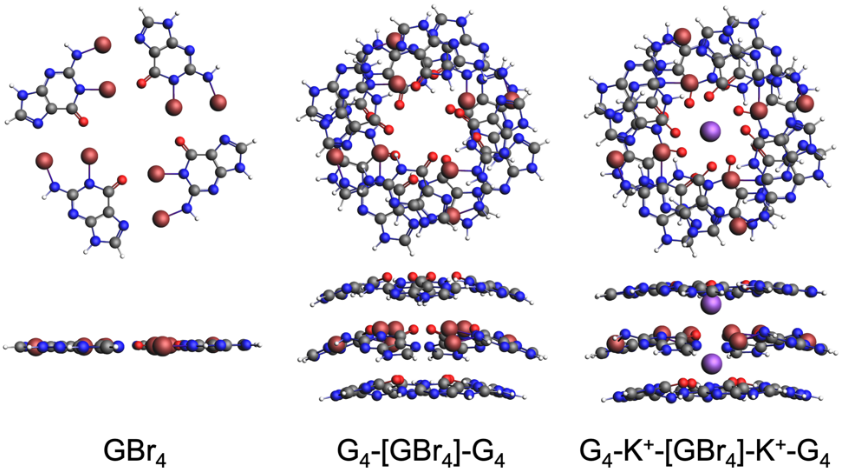

Figure S4. (a) Structures of brominated guanine quadruplex (GBr_4) and two sandwich model complexes of GBr_4 in between two guanine quartets optimized at BLYP-D3(BJ)/TZ2P (taken from L.P. Wolters, N. W. G. Smits, C. Fonseca Guerra, *Phys. Chem. Chem. Phys.* **2015**, *17*, 1585-1592)

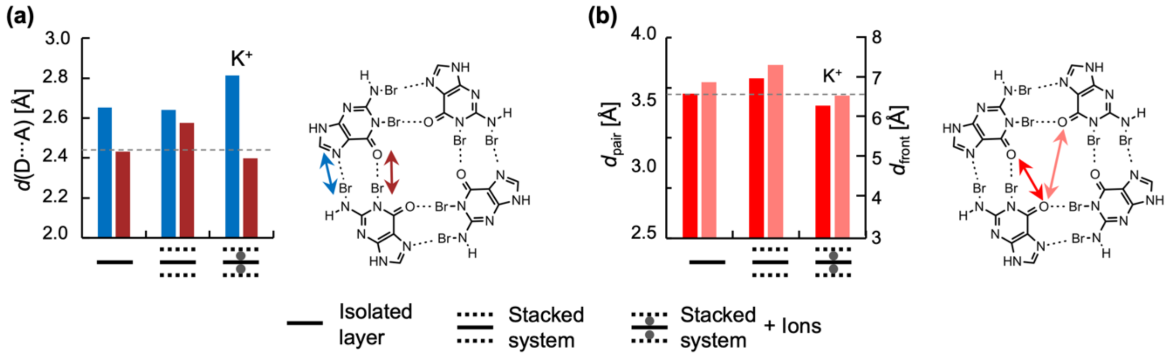

Figure S5. (a) Changes of *d*(Br···A) (Br···O and Br···N) atomic distances within N–Br···A halogen bonds (D = donor, A = acceptor) within the Br-halo-guanine quartet middle layer, and **(b)** changes of O···O atomic distances within GQ middle layer.

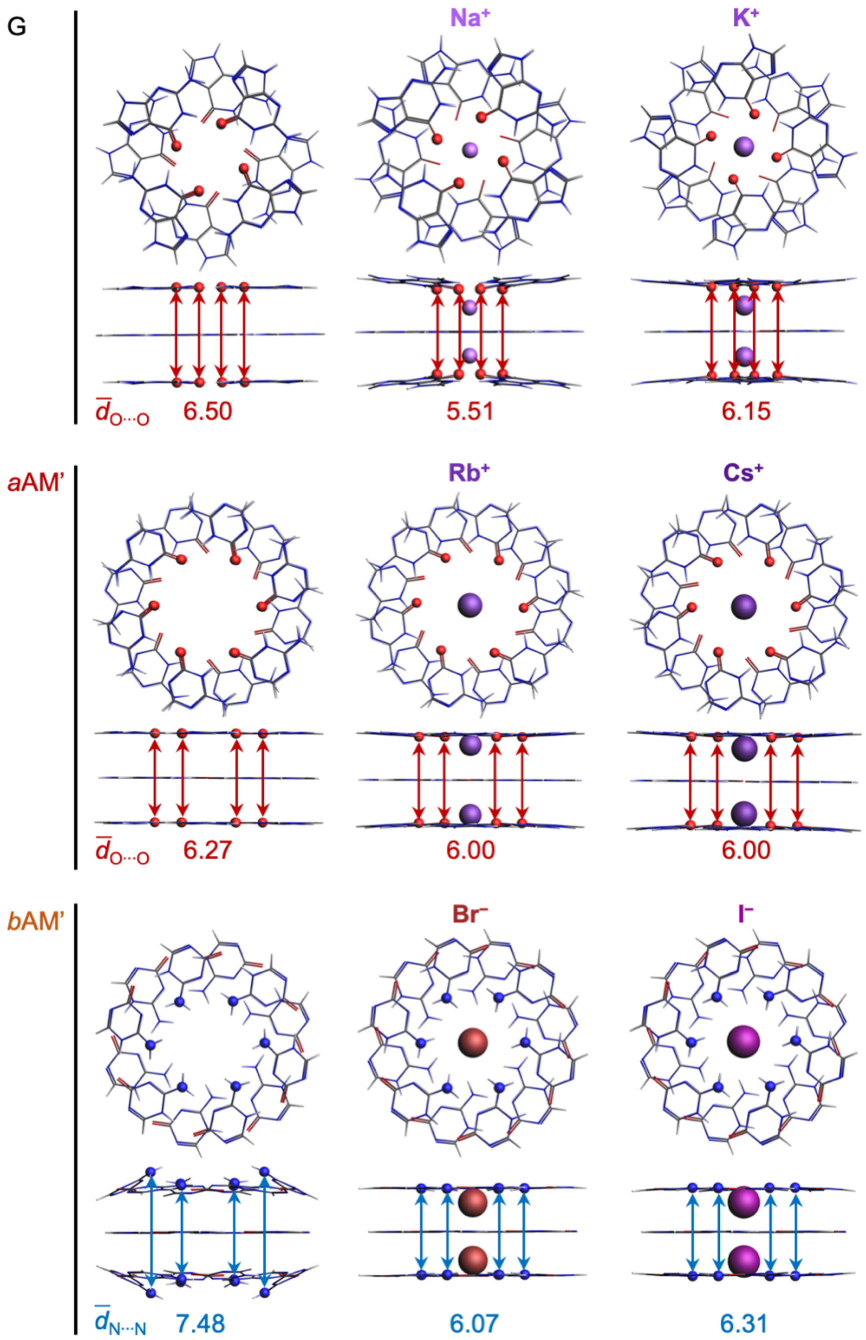

Figure S6. Average O···O and N···N distances from top to bottom layer.

Supporting equations. Individual pairwise interactions for all possible pairs of units in the stack complexes with ions, where m_6 is either *a*AM'$_6$ or *b*AM'$_6$, and i = Rb+, Cs+, Br$^-$, I$^-$.

$$\Delta E_{\text{pair}} = \left(E_{\text{m}_6\text{-i-[m}_2\text{]-i-m}_6} - E_{\text{m}_6\text{-i-[\ ]-i-m}_6}\right) - 2 \cdot \left(E_{\text{m}_6\text{-i-[m]-i-m}_6} - E_{\text{m}_6\text{-i-[\ ]-i-m}_6}\right) \quad \text{(S1)}$$

$$\Delta E_{\text{diag}} = \left(E_{\text{m}_6\text{-i-[m/m]-i-m}_6} - E_{\text{m}_6\text{-i-[\ ]-i-m}_6}\right) - 2 \cdot \left(E_{\text{m}_6\text{-i-[m]-i-m}_6} - E_{\text{m}_6\text{-i-[\ ]-i-m}_6}\right) \quad \text{(S2)}$$

$$\Delta E_{\text{front}} = \left(E_{\text{m}_6\text{-i-[m|m]-i-m}_6} - E_{\text{m}_6\text{-i-[\ ]-i-m}_6}\right) - 2 \cdot \left(E_{\text{m}_6\text{-i-[m]-i-m}_6} - E_{\text{m}_6\text{-i-[\ ]-i-m}_6}\right) \quad \text{(S3)}$$

Table S1. Analysis of interaction energies [kcal mol^{-1}] of guanine quadruplex.

Top layer	[ ]	Bottom layer	[Middle layer]	ΔE_{int}	ΔE_{sum}	ΔE_{syn}	ΔE_{stack}	ΔE_{coor}
G_4-	[ ]	-G_4	G_4	-86.9	-71.4	-15.5	-69.5	-
G_4-	[ ]	-G_4	GBr_4	-56.0[a]	-43.4[a]	-12.7[a]		
G_4-Na^+-	[ ]	-Na^+-G_4	G_4	-57.2	-36.8	-20.4	-61.9	-379.4
G_4-K^+-	[ ]	-K^+-G_4	G_4	-71.6	-55.1	-16.5	-61.9	-312.4
G_4-K^+-	[ ]	-K^+-G_4	GBr_4	-47.0[a]	-29.3[a]	-17.7[a]		

[a] Values taken from: L.P. Wolters, N. W. G. Smits, C. Fonseca Guerra, Covalency in Resonance-Assisted Halogen Bonds Demonstrated with Cooperativity in N-Halo-Guanine Quartets, *Phys. Chem. Chem. Phys.* **2015**, *17*, 1585-1592.

Table S2. Energy decomposition analysis [kcal mol^{-1}] of the stacking energy (interaction energy between the layers) computed at BLYP-D3/TZP//BLYP-D3/TZ2P.

System	ΔE_{stack}	ΔE_{oi}	ΔE_{Pauli}	ΔV_{elstat}	ΔE_{disp}
*a*AM’$_6$-[*a*AM’$_6$]-*a*AM’$_6$	-93,5	-26,7	151,7	-53,7	-164,9
*a*AM’$_6$-Rb^+-[*a*AM’$_6$]-Rb^+-*a*AM’$_6$	-89,2	-30,8	158,7	-46,3	-170,8
*a*AM’$_6$-Cs^+-[*a*AM’$_6$]-Cs^+-*a*AM’$_6$	-89,2	-30,5	157,6	-45,9	-170,3
*b*AM’$_6$-[*b*AM’$_6$]-*b*AM’$_6$	-119,1	-40,9	171,6	-87,1	-162,7
*b*AM’$_6$-Br^--[*b*AM’$_6$]-Br^--*b*AM’$_6$	-109,9	-42,5	189,0	-68,1	-188,3
*b*AM’$_6$-I^--[*b*AM’$_6$]-I^--*b*AM’$_6$	-112,9	-41,5	179,1	-68,2	-182,4
G_4-[G_4]-G_4	-69,5	-22,0	126,6	-35,4	-138,7
G_4-Na^+-[G_4]-Na^+-G_4	-61,9	-28,9	153,4	-32,7	-153,8
G_4-K^+-[G_4]-K^+-G_4	-61,9	-19,3	92,4	-10,1	-124,8

Table S3. Energy decomposition [kcal mol^{-1}] for the formation of the middle quartet [G_{n+1}] from G_n+G in a stepwise one-way direction (n = 1, 2, 3) computed at BLYP-D3/TZP//BLYP-D3/TZ2P.

n+1	ΔE_{int}	ΔE_{oi}	ΔE_{Pauli}	ΔV_{elstat}	ΔE_{disp}
(a) G_4–[G_{n+1}]–G_4					
1+1	-16,0	-16,8	32,4	-27,2	-4,5
2+1	-22,9	-19,1	32,9	-32,1	-4,6
3+1	-50,9	-43,1	64,3	-62,9	-9,1
ΔE_{syn}	-21,5	-11,5	-0,2	-9,8	0,0
(b) G_4-Na^+-[G_{n+1}]-Na^+-G_4					
1+1	-14,2	-18,8	37,3	-27,6	-5,0
2+1	-20,3	-21,0	36,4	-30,5	-5,2
3+1	-47,4	-47,9	74,0	-63,3	-10,2
ΔE_{syn}	-23,4	-11,9	-1,6	-10,0	0,0

Cartesian coordinates

*a*AM'

C	-1.75365530	-4.42062174	-0.30095635
N	-0.48783313	-5.03351814	0.06245395
C	-0.34605608	-6.38194984	0.21450593
N	-1.37003395	-7.21051352	0.03472340
C	-2.54598917	-6.59579834	-0.30010218
N	-2.80156727	-5.31295236	-0.47872794
H	0.27955204	-4.38217069	0.22722749
H	1.71173199	-6.34947453	0.44860761
N	0.86558132	-6.88771567	0.60346245
H	0.95023753	-7.89769968	0.53221668
O	-1.79872200	-3.20319173	-0.41721892
H	-3.38678360	-7.28019560	-0.44141032

(C_s) *a*AM'$_2$

H	-3.92146300	2.04458700	0.00000000
H	-6.32034400	1.75059800	0.00000000
N	-6.39090700	2.77294300	0.00000000
H	-4.51839800	6.74166900	0.00000000
C	-4.86897200	-0.66342300	0.00000000
N	-4.63407800	-2.08007300	0.00000000
C	-5.65876100	-2.98542500	0.00000000
N	-6.93379300	-2.58750100	0.00000000
C	-7.11329600	-1.24294400	0.00000000
N	-6.19860900	-0.28114200	0.00000000
H	-3.65216400	-2.35857700	0.00000000
H	-4.43695400	-4.67584300	0.00000000
N	-5.38116500	-4.31033500	0.00000000
H	-7.30001000	3.21978600	0.00000000
H	-6.16465800	-4.95437700	0.00000000
O	-3.90106800	0.09397200	0.00000000
H	-8.15646900	-0.91845500	0.00000000
O	-1.77694700	3.33470900	0.00000000
C	-2.87593100	3.88347600	0.00000000
N	-4.06163100	3.06323200	0.00000000
C	-5.31593900	3.59971900	0.00000000
N	-5.50954900	4.92516600	0.00000000
C	-4.36082800	5.65880900	0.00000000
N	-3.10050600	5.25574800	0.00000000

(C_s) *a*AM'$_6$

C	-1.85654200	-4.46064500	0.00000000
N	-0.55262700	-5.00347500	0.00000000
C	-0.34512300	-6.36057400	0.00000000
N	-1.38050500	-7.23517100	0.00000000
C	-2.59350700	-6.66842800	0.00000000
N	-2.90242900	-5.37011000	0.00000000
H	0.26139800	-4.34403600	0.00000000
H	1.76119100	-6.23219100	0.00000000
N	0.90299300	-6.83906500	0.00000000
C	5.67623100	2.88701900	0.00000000
H	0.98460300	-7.85026600	0.00000000

O	-2.02145100	-3.23014000	0.00000000
H	-3.43865500	-7.36288900	0.00000000
N	6.95072000	2.42575400	0.00000000
C	2.92108000	-3.82900600	0.00000000
N	4.04289200	-2.97005400	0.00000000
C	5.32221400	-3.46854700	0.00000000
N	5.56227200	-4.80260800	0.00000000
C	4.46580800	-5.57049900	0.00000000
N	3.18687600	-5.18927700	0.00000000
H	3.87992400	-1.93560300	0.00000000
H	6.26909100	-1.58103300	0.00000000
N	6.36147800	-2.62801500	0.00000000
C	7.06463800	1.09181300	0.00000000
H	7.27700800	-3.06501600	0.00000000
O	1.77250000	-3.35798200	0.00000000
H	4.64509400	-6.64950400	0.00000000
N	6.09313800	0.17686000	0.00000000
C	-4.78306200	-0.62934400	0.00000000
N	-4.60323500	-2.03062000	0.00000000
C	-5.67623100	-2.88701900	0.00000000
N	-6.95072000	-2.42575400	0.00000000
C	-7.06463800	-1.09181300	0.00000000
N	-6.09313800	-0.17686000	0.00000000
H	-3.62601400	-2.40785400	0.00000000
H	-4.51750200	-4.65120000	0.00000000
N	-5.47023400	-4.20784900	0.00000000
H	3.62601400	2.40785400	0.00000000
H	-6.30720900	-4.78105500	0.00000000
O	-3.79946200	0.12801700	0.00000000
H	-8.08793400	-0.70558600	0.00000000
H	4.51750200	4.65120000	0.00000000
C	-2.92108000	3.82900600	0.00000000
N	-4.04289200	2.97005400	0.00000000
C	-5.32221400	3.46854700	0.00000000
N	-5.56227200	4.80260800	0.00000000
C	-4.46580800	5.57049900	0.00000000
N	-3.18687600	5.18927700	0.00000000
H	-3.87992400	1.93560300	0.00000000
H	-6.26909100	1.58103300	0.00000000
N	-6.36147800	2.62801500	0.00000000
H	8.08793400	0.70558600	0.00000000
H	-7.27700800	3.06501600	0.00000000
O	-1.77250000	3.35798200	0.00000000
H	-4.64509400	6.64950400	0.00000000
N	5.47023400	4.20784900	0.00000000
C	1.85654200	4.46064500	0.00000000
N	0.55262700	5.00347500	0.00000000
C	0.34512300	6.36057400	0.00000000
N	1.38050500	7.23517100	0.00000000
C	2.59350700	6.66842800	0.00000000
N	2.90242900	5.37011000	0.00000000
H	-0.26139800	4.34403600	0.00000000
H	-1.76119100	6.23219100	0.00000000
N	-0.90299300	6.83906500	0.00000000
H	6.30720900	4.78105500	0.00000000
H	-0.98460300	7.85026600	0.00000000
O	2.02145100	3.23014000	0.00000000
H	3.43865500	7.36288900	0.00000000
O	3.79946200	-0.12801700	0.00000000

C	4.78306200	0.62934400	0.00000000
N	4.60323500	2.03062000	0.00000000

(C_1) *a*AM'$_6$

C	-1.80187600	4.34676700	-0.43247200
N	-3.07127000	3.77169900	-0.64448200
C	-4.12762000	4.52086400	-1.09352900
N	-3.98713600	5.83478000	-1.38983600
C	-2.76113800	6.33126600	-1.17060800
N	-1.67981600	5.70053700	-0.70589800
H	-3.18867000	2.74164300	-0.47853300
H	-5.54268900	2.99334000	-0.85591700
N	-5.32346600	3.93750200	-1.25370900
H	-2.63634100	7.39400100	-1.39669600
H	-6.07554600	4.55618400	-1.53871400
O	-0.86443400	3.64438400	-0.01860400
C	2.84475700	3.77990600	0.48844700
N	1.73411200	4.63436800	0.33265800
C	1.85858200	5.99436000	0.44716200
N	3.04507200	6.57274400	0.75127100
C	4.06296200	5.71464800	0.90883800
N	4.05414100	4.38442400	0.79423600
H	0.79330000	4.20366000	0.15470400
H	-0.12915200	6.40489000	-0.10065700
N	0.78324500	6.77073100	0.26109600
H	5.02608300	6.16811800	1.15963100
H	0.94510100	7.76983200	0.32943200
O	2.70659700	2.55307300	0.35129900
C	4.68499300	-0.59589500	-0.04827700
N	4.85866100	0.75428400	0.31957400
C	6.09358900	1.25059200	0.64782300
N	7.19852100	0.46658900	0.62741600
C	6.98174500	-0.79975900	0.24563700
N	5.82795000	-1.37892100	-0.09565200
H	4.01460700	1.37810000	0.34003600
H	5.42649400	3.22493500	0.95774300
N	6.21454200	2.53543400	1.00412600
H	7.86846700	-1.43891800	0.20864400
H	7.15832700	2.84841700	1.20515800
O	3.54971100	-1.02636400	-0.31134400
C	-4.68499300	0.59589500	-0.04827700
N	-4.85866100	-0.75428400	0.31957400
C	-6.09358900	-1.25059200	0.64782300
N	-7.19852100	-0.46658900	0.62741600
C	-6.98174500	0.79975900	0.24563700
N	-5.82795000	1.37892100	-0.09565200
H	-4.01460700	-1.37810000	0.34003600
H	-5.42649400	-3.22493500	0.95774300
N	-6.21454200	-2.53543400	1.00412600
H	-7.86846700	1.43891800	0.20864400
H	-7.15832700	-2.84841700	1.20515800
O	-3.54971100	1.02636400	-0.31134400
C	1.80187600	-4.34676700	-0.43247200
N	3.07127000	-3.77169900	-0.64448200
C	4.12762000	-4.52086400	-1.09352900
N	3.98713600	-5.83478000	-1.38983600
C	2.76113800	-6.33126600	-1.17060800
N	1.67981600	-5.70053700	-0.70589800

H	3.18867000	-2.74164300	-0.47853300
H	5.54268900	-2.99334000	-0.85591700
N	5.32346600	-3.93750200	-1.25370900
H	2.63634100	-7.39400100	-1.39669600
H	6.07554600	-4.55618400	-1.53871400
O	0.86443400	-3.64438400	-0.01860400
C	-2.84475700	-3.77990600	0.48844700
N	-1.73411200	-4.63436800	0.33265800
C	-1.85858200	-5.99436000	0.44716200
N	-3.04507200	-6.57274400	0.75127100
C	-4.06296200	-5.71464800	0.90883800
N	-4.05414100	-4.38442400	0.79423600
H	-0.79330000	-4.20366000	0.15470400
H	0.12915200	-6.40489000	-0.10065700
N	-0.78324500	-6.77073100	0.26109600
H	-5.02608300	-6.16811800	1.15963100
H	-0.94510100	-7.76983200	0.32943200
O	-2.70659700	-2.55307300	0.35129900

(*C*1) *a*AM’6-[*a*AM’6]-*a*AM’6

C	-3.854692000000	-2.877213000000	-3.142972000000
N	-3.008818000000	-4.007229000000	-3.140401000000
C	-3.519177000000	-5.278512000000	-3.190816000000
N	-4.856077000000	-5.506718000000	-3.198037000000
C	-5.612026000000	-4.402086000000	-3.189704000000
N	-5.217588000000	-3.129324000000	-3.175741000000
H	-1.972778000000	-3.850971000000	-3.110970000000
H	-1.641158000000	-6.243950000000	-3.199501000000
N	-2.689303000000	-6.324232000000	-3.236197000000
H	-3.133015000000	-7.236108000000	-3.243648000000
O	-3.375493000000	-1.734964000000	-3.111290000000
H	-6.692927000000	-4.567413000000	-3.190614000000
C	0.568129000000	-4.777454000000	-3.131735000000
N	1.969955000000	-4.609854000000	-3.133390000000
C	2.815542000000	-5.687951000000	-3.177627000000
N	2.344681000000	-6.959975000000	-3.174176000000
C	1.010179000000	-7.062088000000	-3.162898000000
N	0.105247000000	-6.083984000000	-3.155053000000
H	2.352228000000	-3.634218000000	-3.107974000000
H	4.590551000000	-4.545029000000	-3.199388000000
N	4.136099000000	-5.493299000000	-3.228584000000
H	4.703212000000	-6.333998000000	-3.229349000000
O	-0.181844000000	-3.791516000000	-3.105117000000
H	0.612918000000	-8.080861000000	-3.155416000000
C	4.421834000000	-1.896377000000	-3.138899000000
N	4.979045000000	-0.599320000000	-3.145744000000
C	6.335100000000	-0.407254000000	-3.202850000000
N	7.200276000000	-1.451633000000	-3.206948000000
C	6.620277000000	-2.657820000000	-3.188968000000
N	5.320647000000	-2.951614000000	-3.168492000000
H	4.326131000000	0.220423000000	-3.117925000000
H	6.232376000000	1.701119000000	-3.222019000000
N	6.826390000000	0.833741000000	-3.258597000000
H	7.837974000000	0.905045000000	-3.269264000000
O	3.193036000000	-2.051316000000	-3.102016000000
H	7.303227000000	-3.511756000000	-3.187060000000
C	3.854692000000	2.877213000000	-3.142972000000
N	3.008818000000	4.007229000000	-3.140401000000

C	3.519177000000	5.278512000000	-3.190816000000
N	4.856077000000	5.506718000000	-3.198037000000
C	5.612026000000	4.402086000000	-3.189704000000
N	5.217588000000	3.129324000000	-3.175741000000
H	1.972778000000	3.850971000000	-3.110970000000
H	1.641158000000	6.243950000000	-3.199501000000
N	2.689303000000	6.324232000000	-3.236197000000
H	3.133015000000	7.236108000000	-3.243648000000
O	3.375493000000	1.734964000000	-3.111290000000
H	6.692927000000	4.567413000000	-3.190614000000
C	-0.568129000000	4.777454000000	-3.131735000000
N	-1.969955000000	4.609854000000	-3.133390000000
C	-2.815542000000	5.687951000000	-3.177627000000
N	-2.344681000000	6.959975000000	-3.174176000000
C	-1.010179000000	7.062088000000	-3.162898000000
N	-0.105247000000	6.083984000000	-3.155053000000
H	-2.352228000000	3.634218000000	-3.107974000000
H	-4.590551000000	4.545029000000	-3.199388000000
N	-4.136099000000	5.493299000000	-3.228584000000
H	-4.703212000000	6.333998000000	-3.229349000000
O	0.181844000000	3.791516000000	-3.105117000000
H	-0.612918000000	8.080861000000	-3.155416000000
N	-6.826390000000	-0.833741000000	-3.258597000000
H	-7.837974000000	-0.905045000000	-3.269264000000
O	-3.193036000000	2.051316000000	-3.102016000000
C	-4.421834000000	1.896377000000	-3.138899000000
N	-4.979045000000	0.599320000000	-3.145744000000
H	-7.303227000000	3.511756000000	-3.187060000000
H	-6.232376000000	-1.701119000000	-3.222019000000
H	-4.326131000000	-0.220423000000	-3.117925000000
N	-5.320647000000	2.951614000000	-3.168492000000
C	-6.620277000000	2.657820000000	-3.188968000000
N	-7.200276000000	1.451633000000	-3.206948000000
C	-6.335100000000	0.407254000000	-3.202850000000
C	-2.960942000000	3.764066000000	0.008874000000
N	-4.068735000000	2.891736000000	0.006048000000
C	-5.351423000000	3.370227000000	-0.001918000000
N	-5.614772000000	4.701848000000	-0.002996000000
C	-4.529295000000	5.484080000000	0.001855000000
N	-3.248451000000	5.120617000000	0.007096000000
H	-3.883584000000	1.859172000000	0.006237000000
H	-6.269975000000	1.467839000000	-0.008904000000
N	-6.375765000000	2.515335000000	-0.009219000000
H	-7.300106000000	2.931233000000	-0.015985000000
O	-1.807028000000	3.317541000000	0.013260000000
H	-4.719097000000	6.560851000000	0.001568000000
C	-4.752071000000	-0.688773000000	0.000864000000
N	-4.551051000000	-2.084381000000	0.002815000000
C	-5.606959000000	-2.955466000000	-0.008076000000
N	-6.891815000000	-2.517603000000	-0.016116000000
C	-7.025855000000	-1.186227000000	-0.015804000000
N	-6.070326000000	-0.258982000000	-0.008721000000
H	-3.563567000000	-2.439595000000	0.007252000000
H	-4.416784000000	-4.699184000000	-0.004430000000
N	-5.378147000000	-4.269965000000	-0.011831000000
H	-6.199690000000	-4.863520000000	-0.022376000000
O	-3.787740000000	0.086380000000	0.008128000000
H	-8.053222000000	-0.812027000000	-0.021945000000
C	-1.785240000000	-4.449629000000	0.008706000000

N	-0.475597000000	-4.972198000000	0.013373000000
C	-0.247761000000	-6.322105000000	0.007803000000
N	-1.268888000000	-7.216469000000	0.003380000000
C	-2.489238000000	-6.667892000000	0.001599000000
N	-2.816056000000	-5.377071000000	0.003331000000
H	0.325441000000	-4.294559000000	0.014905000000
H	1.858067000000	-6.162127000000	0.007041000000
N	1.005308000000	-6.779890000000	0.005984000000
H	1.109610000000	-7.788108000000	-0.000726000000
O	-1.975352000000	-3.226875000000	0.010154000000
H	-3.326373000000	-7.371195000000	-0.001519000000
C	2.960942000000	-3.764066000000	0.008874000000
N	4.068735000000	-2.891736000000	0.006048000000
C	5.351423000000	-3.370227000000	-0.001918000000
N	5.614772000000	-4.701848000000	-0.002996000000
C	4.529295000000	-5.484080000000	0.001855000000
N	3.248451000000	-5.120617000000	0.007096000000
H	3.883584000000	-1.859172000000	0.006237000000
H	6.269975000000	-1.467839000000	-0.008904000000
N	6.375765000000	-2.515335000000	-0.009219000000
H	7.300106000000	-2.931233000000	-0.015985000000
O	1.807028000000	-3.317541000000	0.013260000000
H	4.719097000000	-6.560851000000	0.001568000000
N	6.070326000000	0.258982000000	-0.008721000000
N	6.891815000000	2.517603000000	-0.016116000000
C	5.606959000000	2.955466000000	-0.008076000000
C	7.025855000000	1.186227000000	-0.015804000000
H	3.563567000000	2.439595000000	0.007252000000
H	4.416784000000	4.699184000000	-0.004430000000
H	8.053222000000	0.812027000000	-0.021945000000
N	5.378147000000	4.269965000000	-0.011831000000
H	6.199690000000	4.863520000000	-0.022376000000
O	3.787740000000	-0.086380000000	0.008128000000
C	4.752071000000	0.688773000000	0.000864000000
N	4.551051000000	2.084381000000	0.002815000000
C	1.785240000000	4.449629000000	0.008706000000
N	0.475597000000	4.972198000000	0.013373000000
C	0.247761000000	6.322105000000	0.007803000000
N	1.268888000000	7.216469000000	0.003380000000
C	2.489238000000	6.667892000000	0.001599000000
N	2.816056000000	5.377071000000	0.003331000000
H	-0.325441000000	4.294559000000	0.014905000000
H	-1.858067000000	6.162127000000	0.007041000000
N	-1.005308000000	6.779890000000	0.005984000000
H	-1.109610000000	7.788108000000	-0.000726000000
O	1.975352000000	3.226875000000	0.010154000000
H	3.326373000000	7.371195000000	-0.001519000000
C	-3.823622000000	-2.914868000000	3.154931000000
N	-2.966114000000	-4.036017000000	3.159588000000
C	-3.463996000000	-5.312872000000	3.188525000000
N	-4.798264000000	-5.555098000000	3.168817000000
C	-5.565385000000	-4.458318000000	3.155497000000
N	-5.184177000000	-3.181466000000	3.160218000000
H	-1.931430000000	-3.869086000000	3.144191000000
H	-1.576530000000	-6.259136000000	3.212730000000
N	-2.624107000000	-6.350328000000	3.239857000000
H	-3.058167000000	-7.266709000000	3.223763000000
O	-3.355975000000	-1.767473000000	3.142025000000
H	-6.644323000000	-4.634775000000	3.134567000000

C	0.617543000000	-4.770399000000	3.160987000000
N	2.017598000000	-4.589128000000	3.159909000000
C	2.873829000000	-5.659090000000	3.189815000000
N	2.415681000000	-6.935704000000	3.178050000000
C	1.082194000000	-7.050705000000	3.171924000000
N	0.167638000000	-6.081552000000	3.175786000000
H	2.390256000000	-3.609504000000	3.139001000000
H	4.637106000000	-4.498468000000	3.203309000000
N	4.192691000000	-5.451439000000	3.234799000000
H	4.768428000000	-6.286139000000	3.221053000000
O	-0.141950000000	-3.791524000000	3.144567000000
H	0.694929000000	-8.073274000000	3.158483000000
N	4.983542000000	-0.549024000000	3.150917000000
C	6.338263000000	-0.343063000000	3.184559000000
N	7.214145000000	-1.378444000000	3.172942000000
C	6.646289000000	-2.590452000000	3.163166000000
N	5.349571000000	-2.897671000000	3.163712000000
H	4.321901000000	0.264084000000	3.133908000000
H	6.213956000000	1.764060000000	3.201084000000
H	7.337813000000	-3.437371000000	3.149486000000
N	6.817362000000	0.903001000000	3.233056000000
H	7.828101000000	0.985144000000	3.221749000000
O	3.212008000000	-2.019051000000	3.132847000000
C	4.439662000000	-1.851665000000	3.150056000000
C	3.823622000000	2.914868000000	3.154931000000
N	2.966114000000	4.036017000000	3.159588000000
C	3.463996000000	5.312872000000	3.188525000000
N	4.798264000000	5.555098000000	3.168817000000
C	5.565385000000	4.458318000000	3.155497000000
N	5.184177000000	3.181466000000	3.160218000000
H	1.931430000000	3.869086000000	3.144191000000
H	1.576530000000	6.259136000000	3.212730000000
N	2.624107000000	6.350328000000	3.239857000000
H	3.058167000000	7.266709000000	3.223763000000
O	3.355975000000	1.767473000000	3.142025000000
H	6.644323000000	4.634775000000	3.134567000000
C	-0.617543000000	4.770399000000	3.160987000000
N	-2.017598000000	4.589128000000	3.159909000000
C	-2.873829000000	5.659090000000	3.189815000000
N	-2.415681000000	6.935704000000	3.178050000000
C	-1.082194000000	7.050705000000	3.171924000000
N	-0.167638000000	6.081552000000	3.175786000000
H	-2.390256000000	3.609504000000	3.139001000000
H	-4.637106000000	4.498468000000	3.203309000000
N	-4.192691000000	5.451439000000	3.234799000000
H	-4.768428000000	6.286139000000	3.221053000000
O	0.141950000000	3.791524000000	3.144567000000
H	-0.694929000000	8.073274000000	3.158483000000
C	-4.439662000000	1.851665000000	3.150056000000
N	-4.983542000000	0.549024000000	3.150917000000
C	-6.338263000000	0.343063000000	3.184559000000
N	-7.214145000000	1.378444000000	3.172942000000
C	-6.646289000000	2.590452000000	3.163166000000
N	-5.349571000000	2.897671000000	3.163712000000
H	-4.321901000000	-0.264084000000	3.133908000000
H	-6.213956000000	-1.764060000000	3.201084000000
N	-6.817362000000	-0.903001000000	3.233056000000
H	-7.828101000000	-0.985144000000	3.221749000000
O	-3.212008000000	2.019051000000	3.132847000000

H	-7.337813000000	3.437371000000	3.149486000000

aAM'$_6$-[aAM'$_6$]-aAM'$_6$ – C_{2h}			
C	4.263762000000	-2.210955000000	-3.154010000000
N	4.912504000000	-0.957387000000	-3.159775000000
C	6.279684000000	-0.863729000000	-3.195522000000
N	7.067592000000	-1.967664000000	-3.184126000000
C	6.402385000000	-3.128881000000	-3.171848000000
N	5.084804000000	-3.327929000000	-3.168614000000
H	4.320415000000	-0.092535000000	-3.141241000000
H	6.332052000000	1.246938000000	-3.213490000000
N	6.860801000000	0.337985000000	-3.245467000000
H	7.874877000000	0.334997000000	-3.234987000000
O	3.026629000000	-2.277364000000	-3.132517000000
H	7.021893000000	-4.029823000000	-3.159295000000
C	4.048219000000	2.595441000000	-3.157218000000
N	3.288517000000	3.785084000000	-3.156558000000
C	3.892308000000	5.015308000000	-3.185590000000
N	5.242328000000	5.144294000000	-3.172303000000
C	5.913777000000	3.986447000000	-3.166363000000
N	5.426306000000	2.746331000000	-3.171017000000
H	2.243096000000	3.705365000000	-3.138088000000
H	2.090475000000	6.114233000000	-3.200391000000
N	3.142149000000	6.119658000000	-3.230240000000
H	3.650915000000	6.996773000000	-3.215278000000
O	3.485578000000	1.491654000000	-3.141878000000
H	7.003912000000	4.071220000000	-3.152468000000
C	-0.216864000000	4.803459000000	-3.152370000000
N	-1.626731000000	4.738650000000	-3.155206000000
C	-2.391497000000	5.875843000000	-3.185447000000
N	-1.829228000000	7.109918000000	-3.168517000000
C	-0.490899000000	7.114145000000	-3.158250000000
N	0.340391000000	6.072659000000	-3.162665000000
H	-2.079448000000	3.793133000000	-3.137026000000
H	-4.243499000000	4.863549000000	-3.206302000000
N	-3.722593000000	5.777118000000	-3.236270000000
H	-4.228705000000	6.655749000000	-3.219564000000
O	0.458743000000	3.764717000000	-3.136806000000
H	-0.020181000000	8.100988000000	-3.140626000000
C	-4.263762000000	2.210955000000	-3.154010000000
N	-4.912504000000	0.957387000000	-3.159775000000
C	-6.279684000000	0.863729000000	-3.195522000000
N	-7.067592000000	1.967664000000	-3.184126000000
C	-6.402385000000	3.128881000000	-3.171848000000
N	-5.084804000000	3.327929000000	-3.168614000000
H	-4.320415000000	0.092535000000	-3.141241000000
H	-6.332052000000	-1.246938000000	-3.213490000000
N	-6.860801000000	-0.337985000000	-3.245467000000
H	-7.874877000000	-0.334997000000	-3.234987000000
O	-3.026629000000	2.277364000000	-3.132517000000
H	-7.021893000000	4.029823000000	-3.159295000000
C	-4.048219000000	-2.595441000000	-3.157218000000
N	-3.288517000000	-3.785084000000	-3.156558000000
C	-3.892308000000	-5.015308000000	-3.185590000000
N	-5.242328000000	-5.144294000000	-3.172303000000
C	-5.913777000000	-3.986447000000	-3.166363000000
N	-5.426306000000	-2.746331000000	-3.171017000000
H	-2.243096000000	-3.705365000000	-3.138088000000

H	-2.090475000000	-6.114233000000	-3.200391000000
N	-3.142149000000	-6.119658000000	-3.230240000000
H	-3.650915000000	-6.996773000000	-3.215278000000
O	-3.485578000000	-1.491654000000	-3.141878000000
H	-7.003912000000	-4.071220000000	-3.152468000000
N	3.722593000000	-5.777118000000	-3.236270000000
H	4.228705000000	-6.655749000000	-3.219564000000
O	-0.458743000000	-3.764717000000	-3.136806000000
C	0.216864000000	-4.803459000000	-3.152370000000
N	1.626731000000	-4.738650000000	-3.155206000000
H	0.020181000000	-8.100988000000	-3.140626000000
H	4.243499000000	-4.863549000000	-3.206302000000
H	2.079448000000	-3.793133000000	-3.137026000000
N	-0.340391000000	-6.072659000000	-3.162665000000
C	0.490899000000	-7.114145000000	-3.158250000000
N	1.829228000000	-7.109918000000	-3.168517000000
C	2.391497000000	-5.875843000000	-3.185447000000
C	-2.163594000000	-4.279216000000	0.000000000000
N	-0.903929000000	-4.913308000000	0.000000000000
C	-0.794000000000	-6.278068000000	0.000000000000
N	-1.888783000000	-7.080462000000	0.000000000000
C	-3.057336000000	-6.428424000000	0.000000000000
N	-3.270796000000	-5.114229000000	0.000000000000
H	-0.047308000000	-4.308037000000	0.000000000000
H	1.318224000000	-6.304941000000	0.000000000000
N	0.414174000000	-6.844304000000	0.000000000000
H	0.428393000000	-7.857808000000	0.000000000000
O	-2.247561000000	-3.044821000000	0.000000000000
H	-3.952395000000	-7.056290000000	0.000000000000
C	2.630671000000	-4.012166000000	0.000000000000
N	3.810931000000	-3.240167000000	0.000000000000
C	5.046856000000	-3.829054000000	0.000000000000
N	5.192537000000	-5.178506000000	0.000000000000
C	4.042332000000	-5.862619000000	0.000000000000
N	2.798110000000	-5.388853000000	0.000000000000
H	3.716540000000	-2.195205000000	0.000000000000
H	6.125790000000	-2.013605000000	0.000000000000
N	6.141683000000	-3.066255000000	0.000000000000
H	7.026626000000	-3.560496000000	0.000000000000
O	1.520632000000	-3.466027000000	0.000000000000
H	4.137039000000	-6.951916000000	0.000000000000
C	4.793778000000	0.267085000000	0.000000000000
N	4.713296000000	1.674849000000	0.000000000000
C	5.840326000000	2.452316000000	0.000000000000
N	7.082387000000	1.904899000000	0.000000000000
C	7.101748000000	0.566850000000	0.000000000000
N	6.070082000000	-0.274928000000	0.000000000000
H	3.760582000000	2.114104000000	0.000000000000
H	4.806079000000	4.293549000000	0.000000000000
N	5.725881000000	3.781624000000	0.000000000000
H	6.595805000000	4.301777000000	0.000000000000
O	3.766251000000	-0.422419000000	0.000000000000
H	8.092997000000	0.105431000000	0.000000000000
C	2.163594000000	4.279216000000	0.000000000000
N	0.903929000000	4.913308000000	0.000000000000
C	0.794000000000	6.278068000000	0.000000000000
N	1.888783000000	7.080462000000	0.000000000000
C	3.057336000000	6.428424000000	0.000000000000
N	3.270796000000	5.114229000000	0.000000000000

H	0.047308000000	4.308037000000	0.000000000000
H	-1.318224000000	6.304941000000	0.000000000000
N	-0.414174000000	6.844304000000	0.000000000000
H	-0.428393000000	7.857808000000	0.000000000000
O	2.247561000000	3.044821000000	0.000000000000
H	3.952395000000	7.056290000000	0.000000000000
N	-2.798110000000	5.388853000000	0.000000000000
N	-5.192537000000	5.178506000000	0.000000000000
C	-5.046856000000	3.829054000000	0.000000000000
C	-4.042332000000	5.862619000000	0.000000000000
H	-3.716540000000	2.195205000000	0.000000000000
H	-6.125790000000	2.013605000000	0.000000000000
H	-4.137039000000	6.951916000000	0.000000000000
N	-6.141683000000	3.066255000000	0.000000000000
H	-7.026626000000	3.560496000000	0.000000000000
O	-1.520632000000	3.466027000000	0.000000000000
C	-2.630671000000	4.012166000000	0.000000000000
N	-3.810931000000	3.240167000000	0.000000000000
C	-4.793778000000	-0.267085000000	0.000000000000
N	-4.713296000000	-1.674849000000	0.000000000000
C	-5.840326000000	-2.452316000000	0.000000000000
N	-7.082387000000	-1.904899000000	0.000000000000
C	-7.101748000000	-0.566850000000	0.000000000000
N	-6.070082000000	0.274928000000	0.000000000000
H	-3.760582000000	-2.114104000000	0.000000000000
H	-4.806079000000	-4.293549000000	0.000000000000
N	-5.725881000000	-3.781624000000	0.000000000000
H	-6.595805000000	-4.301777000000	0.000000000000
O	-3.766251000000	0.422419000000	0.000000000000
H	-8.092997000000	-0.105431000000	0.000000000000
C	4.263762000000	-2.210955000000	3.154010000000
N	4.912504000000	-0.957387000000	3.159775000000
C	6.279684000000	-0.863729000000	3.195522000000
N	7.067592000000	-1.967664000000	3.184126000000
C	6.402385000000	-3.128881000000	3.171848000000
N	5.084804000000	-3.327929000000	3.168614000000
H	4.320415000000	-0.092535000000	3.141241000000
H	6.332052000000	1.246938000000	3.213490000000
N	6.860801000000	0.337985000000	3.245467000000
H	7.874877000000	0.334997000000	3.234987000000
O	3.026629000000	-2.277364000000	3.132517000000
H	7.021893000000	-4.029823000000	3.159295000000
C	4.048219000000	2.595441000000	3.157218000000
N	3.288517000000	3.785084000000	3.156558000000
C	3.892308000000	5.015308000000	3.185590000000
N	5.242328000000	5.144294000000	3.172303000000
C	5.913777000000	3.986447000000	3.166363000000
N	5.426306000000	2.746331000000	3.171017000000
H	2.243096000000	3.705365000000	3.138088000000
H	2.090475000000	6.114233000000	3.200391000000
N	3.142149000000	6.119658000000	3.230240000000
H	3.650915000000	6.996773000000	3.215278000000
O	3.485578000000	1.491654000000	3.141878000000
H	7.003912000000	4.071220000000	3.152468000000
N	-1.626731000000	4.738650000000	3.155206000000
C	-2.391497000000	5.875843000000	3.185447000000
N	-1.829228000000	7.109918000000	3.168517000000
C	-0.490899000000	7.114145000000	3.158250000000
N	0.340391000000	6.072659000000	3.162665000000

H	-2.079448000000	3.793133000000	3.137026000000
H	-4.243499000000	4.863549000000	3.206302000000
H	-0.020181000000	8.100988000000	3.140626000000
N	-3.722593000000	5.777118000000	3.236270000000
H	-4.228705000000	6.655749000000	3.219564000000
O	0.458743000000	3.764717000000	3.136806000000
C	-0.216864000000	4.803459000000	3.152370000000
C	-4.263762000000	2.210955000000	3.154010000000
N	-4.912504000000	0.957387000000	3.159775000000
C	-6.279684000000	0.863729000000	3.195522000000
N	-7.067592000000	1.967664000000	3.184126000000
C	-6.402385000000	3.128881000000	3.171848000000
N	-5.084804000000	3.327929000000	3.168614000000
H	-4.320415000000	0.092535000000	3.141241000000
H	-6.332052000000	-1.246938000000	3.213490000000
N	-6.860801000000	-0.337985000000	3.245467000000
H	-7.874877000000	-0.334997000000	3.234987000000
O	-3.026629000000	2.277364000000	3.132517000000
H	-7.021893000000	4.029823000000	3.159295000000
C	-4.048219000000	-2.595441000000	3.157218000000
N	-3.288517000000	-3.785084000000	3.156558000000
C	-3.892308000000	-5.015308000000	3.185590000000
N	-5.242328000000	-5.144294000000	3.172303000000
C	-5.913777000000	-3.986447000000	3.166363000000
N	-5.426306000000	-2.746331000000	3.171017000000
H	-2.243096000000	-3.705365000000	3.138088000000
H	-2.090475000000	-6.114233000000	3.200391000000
N	-3.142149000000	-6.119658000000	3.230240000000
H	-3.650915000000	-6.996773000000	3.215278000000
O	-3.485578000000	-1.491654000000	3.141878000000
H	-7.003912000000	-4.071220000000	3.152468000000
C	0.216864000000	-4.803459000000	3.152370000000
N	1.626731000000	-4.738650000000	3.155206000000
C	2.391497000000	-5.875843000000	3.185447000000
N	1.829228000000	-7.109918000000	3.168517000000
C	0.490899000000	-7.114145000000	3.158250000000
N	-0.340391000000	-6.072659000000	3.162665000000
H	2.079448000000	-3.793133000000	3.137026000000
H	4.243499000000	-4.863549000000	3.206302000000
N	3.722593000000	-5.777118000000	3.236270000000
H	4.228705000000	-6.655749000000	3.219564000000
O	-0.458743000000	-3.764717000000	3.136806000000
H	0.020181000000	-8.100988000000	3.140626000000

(C_{2h}) aAM'$_6$-Rb^+-[aAM'$_6$]-Rb^+-aAM'$_6$

C	4.131453000000	-2.089102000000	-3.083401000000
N	4.832094000000	-0.877238000000	-3.110518000000
C	6.205617000000	-0.849752000000	-3.202729000000
N	6.933137000000	-1.990233000000	-3.241266000000
C	6.214828000000	-3.113280000000	-3.216254000000
N	4.884475000000	-3.241601000000	-3.146114000000
H	4.261996000000	0.003849000000	-3.064035000000
H	6.293126000000	1.225635000000	-3.215186000000
N	6.831802000000	0.324132000000	-3.244349000000
H	7.841674000000	0.297136000000	-3.341858000000
O	2.882509000000	-2.090717000000	-2.996286000000
H	6.784209000000	-4.045385000000	-3.249906000000
C	3.872771000000	2.532286000000	-3.082839000000

N	3.173565000000	3.745318000000	-3.106212000000
C	3.836956000000	4.948660000000	-3.193957000000
N	5.188456000000	5.008656000000	-3.231115000000
C	5.801756000000	3.824993000000	-3.210225000000
N	5.247397000000	2.608569000000	-3.145420000000
H	2.125382000000	3.692121000000	-3.062301000000
H	2.083952000000	6.062815000000	-3.209083000000
N	3.134240000000	6.078501000000	-3.232652000000
H	3.663498000000	6.939190000000	-3.328696000000
O	3.250185000000	1.449365000000	-2.999800000000
H	6.893703000000	3.852027000000	-3.243252000000
C	-0.258486000000	4.619268000000	-3.086427000000
N	-1.658548000000	4.621791000000	-3.109669000000
C	-2.368054000000	5.798487000000	-3.196630000000
N	-1.743233000000	6.998320000000	-3.232493000000
C	-0.411523000000	6.936062000000	-3.211775000000
N	0.364103000000	5.847161000000	-3.148733000000
H	-2.137342000000	3.687875000000	-3.062926000000
H	-4.209527000000	4.837507000000	-3.210069000000
N	-3.697805000000	5.754709000000	-3.236943000000
H	-4.179161000000	6.643409000000	-3.330196000000
O	0.366445000000	3.537599000000	-3.003651000000
H	0.111913000000	7.894801000000	-3.243629000000
C	-4.131453000000	2.089102000000	-3.083401000000
N	-4.832094000000	0.877238000000	-3.110518000000
C	-6.205617000000	0.849752000000	-3.202729000000
N	-6.933137000000	1.990233000000	-3.241266000000
C	-6.214828000000	3.113280000000	-3.216254000000
N	-4.884475000000	3.241601000000	-3.146114000000
H	-4.261996000000	-0.003849000000	-3.064035000000
H	-6.293126000000	-1.225635000000	-3.215186000000
N	-6.831802000000	-0.324132000000	-3.244349000000
H	-7.841674000000	-0.297136000000	-3.341858000000
O	-2.882509000000	2.090717000000	-2.996286000000
H	-6.784209000000	4.045385000000	-3.249906000000
C	-3.872771000000	-2.532286000000	-3.082839000000
N	-3.173565000000	-3.745318000000	-3.106212000000
C	-3.836956000000	-4.948660000000	-3.193957000000
N	-5.188456000000	-5.008656000000	-3.231115000000
C	-5.801756000000	-3.824993000000	-3.210225000000
N	-5.247397000000	-2.608569000000	-3.145420000000
H	-2.125382000000	-3.692121000000	-3.062301000000
H	-2.083952000000	-6.062815000000	-3.209083000000
N	-3.134240000000	-6.078501000000	-3.232652000000
H	-3.663498000000	-6.939190000000	-3.328696000000
O	-3.250185000000	-1.449365000000	-2.999800000000
H	-6.893703000000	-3.852027000000	-3.243252000000
N	3.697805000000	-5.754709000000	-3.236943000000
H	4.179161000000	-6.643409000000	-3.330196000000
O	-0.366445000000	-3.537599000000	-3.003651000000
C	0.258486000000	-4.619268000000	-3.086427000000
N	1.658548000000	-4.621791000000	-3.109669000000
H	-0.111913000000	-7.894801000000	-3.243629000000
H	4.209527000000	-4.837507000000	-3.210069000000
H	2.137342000000	-3.687875000000	-3.062926000000
N	-0.364103000000	-5.847161000000	-3.148733000000
C	0.411523000000	-6.936062000000	-3.211775000000
N	1.743233000000	-6.998320000000	-3.232493000000
C	2.368054000000	-5.798487000000	-3.196630000000

C	-1.976070000000	-4.188584000000	0.000000000000
N	-0.739741000000	-4.845230000000	0.000000000000
C	-0.662440000000	-6.217275000000	0.000000000000
N	-1.774300000000	-6.990379000000	0.000000000000
C	-2.923460000000	-6.313581000000	0.000000000000
N	-3.099996000000	-4.988695000000	0.000000000000
H	0.121847000000	-4.241808000000	0.000000000000
H	1.420638000000	-6.236184000000	0.000000000000
N	0.533103000000	-6.801483000000	0.000000000000
H	0.545337000000	-7.815932000000	0.000000000000
O	-2.026752000000	-2.941084000000	0.000000000000
H	-3.834503000000	-6.916616000000	0.000000000000
C	2.649664000000	-3.807551000000	0.000000000000
N	3.837420000000	-3.066611000000	0.000000000000
C	5.063414000000	-3.686927000000	0.000000000000
N	5.175741000000	-5.036468000000	0.000000000000
C	4.014096000000	-5.691813000000	0.000000000000
N	2.778964000000	-5.181097000000	0.000000000000
H	3.745698000000	-2.018511000000	0.000000000000
H	6.120760000000	-1.892914000000	0.000000000000
N	6.167460000000	-2.944007000000	0.000000000000
H	7.052200000000	-3.440462000000	0.000000000000
O	1.545002000000	-3.226072000000	0.000000000000
H	4.079657000000	-6.782438000000	0.000000000000
C	4.626708000000	0.384324000000	0.000000000000
N	4.576420000000	1.783212000000	0.000000000000
C	5.725278000000	2.536943000000	0.000000000000
N	6.950805000000	1.961057000000	0.000000000000
C	6.939887000000	0.627327000000	0.000000000000
N	5.881468000000	-0.188966000000	0.000000000000
H	3.622060000000	2.225568000000	0.000000000000
H	4.696248000000	4.346874000000	0.000000000000
N	5.631160000000	3.864218000000	0.000000000000
H	6.502062000000	4.384495000000	0.000000000000
O	3.571308000000	-0.282515000000	0.000000000000
H	7.918077000000	0.140577000000	0.000000000000
C	1.976070000000	4.188584000000	0.000000000000
N	0.739741000000	4.845230000000	0.000000000000
C	0.662440000000	6.217275000000	0.000000000000
N	1.774300000000	6.990379000000	0.000000000000
C	2.923460000000	6.313581000000	0.000000000000
N	3.099996000000	4.988695000000	0.000000000000
H	-0.121847000000	4.241808000000	0.000000000000
H	-1.420638000000	6.236184000000	0.000000000000
N	-0.533103000000	6.801483000000	0.000000000000
H	-0.545337000000	7.815932000000	0.000000000000
O	2.026752000000	2.941084000000	0.000000000000
H	3.834503000000	6.916616000000	0.000000000000
N	-2.778964000000	5.181097000000	0.000000000000
N	-5.175741000000	5.036468000000	0.000000000000
C	-5.063414000000	3.686927000000	0.000000000000
C	-4.014096000000	5.691813000000	0.000000000000
H	-3.745698000000	2.018511000000	0.000000000000
H	-6.120760000000	1.892914000000	0.000000000000
H	-4.079657000000	6.782438000000	0.000000000000
N	-6.167460000000	2.944007000000	0.000000000000
H	-7.052200000000	3.440462000000	0.000000000000
O	-1.545002000000	3.226072000000	0.000000000000
C	-2.649664000000	3.807551000000	0.000000000000

N	-3.837420000000	3.066611000000	0.000000000000
C	-4.626708000000	-0.384324000000	0.000000000000
N	-4.576420000000	-1.783212000000	0.000000000000
C	-5.725278000000	-2.536943000000	0.000000000000
N	-6.950805000000	-1.961057000000	0.000000000000
C	-6.939887000000	-0.627327000000	0.000000000000
N	-5.881468000000	0.188966000000	0.000000000000
H	-3.622060000000	-2.225568000000	0.000000000000
H	-4.696248000000	-4.346874000000	0.000000000000
N	-5.631160000000	-3.864218000000	0.000000000000
H	-6.502062000000	-4.384495000000	0.000000000000
O	-3.571308000000	0.282515000000	0.000000000000
H	-7.918077000000	-0.140577000000	0.000000000000
C	4.131453000000	-2.089102000000	3.083401000000
N	4.832094000000	-0.877238000000	3.110518000000
C	6.205617000000	-0.849752000000	3.202729000000
N	6.933137000000	-1.990233000000	3.241266000000
C	6.214828000000	-3.113280000000	3.216254000000
N	4.884475000000	-3.241601000000	3.146114000000
H	4.261996000000	0.003849000000	3.064035000000
H	6.293126000000	1.225635000000	3.215186000000
N	6.831802000000	0.324132000000	3.244349000000
H	7.841674000000	0.297136000000	3.341858000000
O	2.882509000000	-2.090717000000	2.996286000000
H	6.784209000000	-4.045385000000	3.249906000000
C	3.872771000000	2.532286000000	3.082839000000
N	3.173565000000	3.745318000000	3.106212000000
C	3.836956000000	4.948660000000	3.193957000000
N	5.188456000000	5.008656000000	3.231115000000
C	5.801756000000	3.824993000000	3.210225000000
N	5.247397000000	2.608569000000	3.145420000000
H	2.125382000000	3.692121000000	3.062301000000
H	2.083952000000	6.062815000000	3.209083000000
N	3.134240000000	6.078501000000	3.232652000000
H	3.663498000000	6.939190000000	3.328696000000
O	3.250185000000	1.449365000000	2.999800000000
H	6.893703000000	3.852027000000	3.243252000000
N	-1.658548000000	4.621791000000	3.109669000000
C	-2.368054000000	5.798487000000	3.196630000000
N	-1.743233000000	6.998320000000	3.232493000000
C	-0.411523000000	6.936062000000	3.211775000000
N	0.364103000000	5.847161000000	3.148733000000
H	-2.137342000000	3.687875000000	3.062926000000
H	-4.209527000000	4.837507000000	3.210069000000
H	0.111913000000	7.894801000000	3.243629000000
N	-3.697805000000	5.754709000000	3.236943000000
H	-4.179161000000	6.643409000000	3.330196000000
O	0.366445000000	3.537599000000	3.003651000000
C	-0.258486000000	4.619268000000	3.086427000000
C	-4.131453000000	2.089102000000	3.083401000000
N	-4.832094000000	0.877238000000	3.110518000000
C	-6.205617000000	0.849752000000	3.202729000000
N	-6.933137000000	1.990233000000	3.241266000000
C	-6.214828000000	3.113280000000	3.216254000000
N	-4.884475000000	3.241601000000	3.146114000000
H	-4.261996000000	-0.003849000000	3.064035000000
H	-6.293126000000	-1.225635000000	3.215186000000
N	-6.831802000000	-0.324132000000	3.244349000000
H	-7.841674000000	-0.297136000000	3.341858000000

O	-2.882509000000	2.090717000000	2.996286000000
H	-6.784209000000	4.045385000000	3.249906000000
C	-3.872771000000	-2.532286000000	3.082839000000
N	-3.173565000000	-3.745318000000	3.106212000000
C	-3.836956000000	-4.948660000000	3.193957000000
N	-5.188456000000	-5.008656000000	3.231115000000
C	-5.801756000000	-3.824993000000	3.210225000000
N	-5.247397000000	-2.608569000000	3.145420000000
H	-2.125382000000	-3.692121000000	3.062301000000
H	-2.083952000000	-6.062815000000	3.209083000000
N	-3.134240000000	-6.078501000000	3.232652000000
H	-3.663498000000	-6.939190000000	3.328696000000
O	-3.250185000000	-1.449365000000	2.999800000000
H	-6.893703000000	-3.852027000000	3.243252000000
C	0.258486000000	-4.619268000000	3.086427000000
N	1.658548000000	-4.621791000000	3.109669000000
C	2.368054000000	-5.798487000000	3.196630000000
N	1.743233000000	-6.998320000000	3.232493000000
C	0.411523000000	-6.936062000000	3.211775000000
N	-0.364103000000	-5.847161000000	3.148733000000
H	2.137342000000	-3.687875000000	3.062926000000
H	4.209527000000	-4.837507000000	3.210069000000
N	3.697805000000	-5.754709000000	3.236943000000
H	4.179161000000	-6.643409000000	3.330196000000
O	-0.366445000000	-3.537599000000	3.003651000000
H	-0.111913000000	-7.894801000000	3.243629000000
Rb	0.000000000000	0.000000000000	-2.440003000000
Rb	0.000000000000	0.000000000000	2.440003000000

(C_1) *a*AM’$_6$-Rb$^+$-[*a*AM’$_6$]-Rb$^+$-*a*AM’$_6$

C	-1.593591000000	-4.158465000000	0.744898000000
N	-0.318423000000	-4.656783000000	1.019012000000
C	-0.118271000000	-5.983204000000	1.295342000000
N	-1.155537000000	-6.843002000000	1.417115000000
C	-2.356644000000	-6.307324000000	1.174372000000
N	-2.646669000000	-5.046682000000	0.834363000000
H	0.483294000000	-3.992590000000	0.860576000000
H	1.942922000000	-5.828751000000	1.162731000000
N	1.131715000000	-6.425674000000	1.455097000000
H	1.244012000000	-7.421875000000	1.613695000000
O	-1.733924000000	-2.952756000000	0.450575000000
H	-3.207957000000	-6.985550000000	1.266813000000
C	2.818567000000	-3.418144000000	0.138040000000
N	3.820243000000	-2.666905000000	-0.485230000000
C	5.071078000000	-3.185296000000	-0.702087000000
N	5.375368000000	-4.458366000000	-0.361276000000
C	4.377444000000	-5.136348000000	0.213055000000
N	3.139110000000	-4.713010000000	0.487595000000
H	3.595061000000	-1.658725000000	-0.685654000000
H	5.842567000000	-1.383914000000	-1.387168000000
N	6.001332000000	-2.413490000000	-1.268768000000
H	6.933450000000	-2.808606000000	-1.341555000000
O	1.695967000000	-2.907576000000	0.337518000000
H	4.602801000000	-6.167012000000	0.495612000000
C	5.263638000000	2.956690000000	-0.562365000000
N	6.423592000000	2.530398000000	-1.115624000000
C	6.437853000000	1.239757000000	-1.457698000000
N	5.455985000000	0.338978000000	-1.332972000000

H	3.274859000000	2.446897000000	-0.003358000000
H	4.252812000000	4.595789000000	0.223827000000
H	7.368607000000	0.873216000000	-1.896113000000
N	5.147066000000	4.229533000000	-0.183284000000
O	3.268719000000	0.028040000000	-0.635862000000
C	4.263706000000	0.770241000000	-0.788844000000
N	4.200246000000	2.107384000000	-0.385619000000
H	5.916753000000	4.845949000000	-0.424113000000
C	1.593591000000	4.158465000000	0.744898000000
N	0.318423000000	4.656783000000	1.019012000000
C	0.118271000000	5.983204000000	1.295342000000
N	1.155537000000	6.843002000000	1.417115000000
C	2.356644000000	6.307324000000	1.174372000000
N	2.646669000000	5.046682000000	0.834363000000
H	-0.483294000000	3.992590000000	0.860576000000
H	-1.942922000000	5.828751000000	1.162731000000
N	-1.131715000000	6.425674000000	1.455097000000
H	-1.244012000000	7.421875000000	1.613695000000
O	1.733924000000	2.952756000000	0.450575000000
H	3.207957000000	6.985550000000	1.266813000000
C	-2.818567000000	3.418144000000	0.138040000000
N	-3.820243000000	2.666905000000	-0.485230000000
C	-5.071078000000	3.185296000000	-0.702087000000
N	-5.375368000000	4.458366000000	-0.361276000000
C	-4.377444000000	5.136348000000	0.213055000000
N	-3.139110000000	4.713010000000	0.487595000000
H	-3.595061000000	1.658725000000	-0.685654000000
H	-5.842567000000	1.383914000000	-1.387168000000
N	-6.001332000000	2.413490000000	-1.268768000000
H	-6.933450000000	2.808606000000	-1.341555000000
O	-1.695967000000	2.907576000000	0.337518000000
H	-4.602801000000	6.167012000000	0.495612000000
C	-4.263706000000	-0.770241000000	-0.788844000000
N	-4.200246000000	-2.107384000000	-0.385619000000
C	-5.263638000000	-2.956690000000	-0.562365000000
N	-6.423592000000	-2.530398000000	-1.115624000000
C	-6.437853000000	-1.239757000000	-1.457698000000
N	-5.455985000000	-0.338978000000	-1.332972000000
H	-3.274859000000	-2.446897000000	-0.003358000000
H	-4.252812000000	-4.595789000000	0.223827000000
N	-5.147066000000	-4.229533000000	-0.183284000000
H	-5.916753000000	-4.845949000000	-0.424113000000
O	-3.268719000000	-0.028040000000	-0.635862000000
H	-7.368607000000	-0.873216000000	-1.896113000000
C	-5.668520000000	0.714578000000	-4.624985000000
N	-6.375356000000	1.840020000000	-4.880491000000
C	-5.734044000000	2.979875000000	-4.613011000000
N	-4.502740000000	3.139156000000	-4.110469000000
H	-3.847038000000	-0.101353000000	-3.923364000000
H	-5.735232000000	-1.372517000000	-4.618783000000
H	-6.290481000000	3.897253000000	-4.820002000000
N	-6.230729000000	-0.475556000000	-4.840631000000
H	-7.160650000000	-0.467917000000	-5.248290000000
O	-2.630680000000	2.018259000000	-3.338831000000
C	-3.779936000000	1.998590000000	-3.840567000000
N	-4.384927000000	0.773742000000	-4.139218000000
C	-3.570828000000	-2.644474000000	-3.532196000000
N	-2.972539000000	-3.780801000000	-2.972305000000
C	-3.626724000000	-4.985464000000	-2.933182000000

N	-4.884981000000	-5.117796000000	-3.409615000000
C	-5.404839000000	-4.005791000000	-3.940031000000
N	-4.839244000000	-2.795804000000	-4.043547000000
H	-1.982801000000	-3.674837000000	-2.623405000000
H	-2.010577000000	-5.996392000000	-2.102095000000
N	-3.014248000000	-6.040344000000	-2.385466000000
H	-3.500306000000	-6.928882000000	-2.454621000000
O	-2.953865000000	-1.554331000000	-3.522522000000
H	-6.419788000000	-4.097103000000	-4.332360000000
C	0.320331000000	-4.547098000000	-2.288775000000
N	1.672746000000	-4.530852000000	-2.636254000000
C	2.431083000000	-5.675529000000	-2.601340000000
N	1.916689000000	-6.844905000000	-2.155588000000
C	0.618265000000	-6.803565000000	-1.843009000000
N	-0.217417000000	-5.755942000000	-1.895797000000
H	2.067165000000	-3.603329000000	-2.942808000000
H	4.072650000000	-4.740697000000	-3.444574000000
N	3.699813000000	-5.614434000000	-3.004050000000
H	4.230873000000	-6.479101000000	-2.990233000000
O	-0.339989000000	-3.486121000000	-2.348467000000
H	0.181450000000	-7.743322000000	-1.497145000000
C	3.779936000000	-1.998590000000	-3.840567000000
N	4.384927000000	-0.773742000000	-4.139218000000
C	5.668520000000	-0.714578000000	-4.624985000000
N	6.375356000000	-1.840020000000	-4.880491000000
C	5.734044000000	-2.979875000000	-4.613011000000
N	4.502740000000	-3.139156000000	-4.110469000000
H	3.847038000000	0.101353000000	-3.923364000000
H	5.735232000000	1.372517000000	-4.618783000000
N	6.230729000000	0.475556000000	-4.840631000000
H	7.160650000000	0.467917000000	-5.248290000000
O	2.630680000000	-2.018259000000	-3.338831000000
H	6.290481000000	-3.897253000000	-4.820002000000
C	3.570828000000	2.644474000000	-3.532196000000
N	2.972539000000	3.780801000000	-2.972305000000
C	3.626724000000	4.985464000000	-2.933182000000
N	4.884981000000	5.117796000000	-3.409615000000
C	5.404839000000	4.005791000000	-3.940031000000
N	4.839244000000	2.795804000000	-4.043547000000
H	1.982801000000	3.674837000000	-2.623405000000
H	2.010577000000	5.996392000000	-2.102095000000
N	3.014248000000	6.040344000000	-2.385466000000
H	3.500306000000	6.928882000000	-2.454621000000
O	2.953865000000	1.554331000000	-3.522522000000
H	6.419788000000	4.097103000000	-4.332360000000
C	-0.320331000000	4.547098000000	-2.288775000000
N	-1.672746000000	4.530852000000	-2.636254000000
C	-2.431083000000	5.675529000000	-2.601340000000
N	-1.916689000000	6.844905000000	-2.155588000000
C	-0.618265000000	6.803565000000	-1.843009000000
N	0.217417000000	5.755942000000	-1.895797000000
H	-2.067165000000	3.603329000000	-2.942808000000
H	-4.072650000000	4.740697000000	-3.444574000000
N	-3.699813000000	5.614434000000	-3.004050000000
H	-4.230873000000	6.479101000000	-2.990233000000
O	0.339989000000	3.486121000000	-2.348467000000
H	-0.181450000000	7.743322000000	-1.497145000000
C	3.447783000000	2.799761000000	3.207915000000
N	2.648535000000	3.777575000000	3.810078000000

C	3.150471000000	5.022269000000	4.108227000000
N	4.433428000000	5.348227000000	3.824422000000
C	5.146164000000	4.377075000000	3.249922000000
N	4.751578000000	3.139966000000	2.923166000000
H	1.631068000000	3.550448000000	3.940335000000
H	1.326353000000	5.747727000000	4.786140000000
N	2.354857000000	5.921485000000	4.685092000000
H	2.754450000000	6.840277000000	4.849395000000
O	2.961361000000	1.674691000000	2.953044000000
H	6.182160000000	4.628006000000	3.009519000000
C	-0.842858000000	4.198373000000	4.069583000000
N	-2.197959000000	4.122535000000	3.729998000000
C	-3.050317000000	5.173034000000	3.958777000000
N	-2.614308000000	6.323893000000	4.519817000000
C	-1.314935000000	6.338251000000	4.831255000000
N	-0.407468000000	5.370731000000	4.645752000000
H	-2.548248000000	3.211609000000	3.331524000000
H	-4.709315000000	4.192758000000	3.160480000000
N	-4.333912000000	5.059440000000	3.606409000000
H	-4.941080000000	5.835517000000	3.849948000000
O	-0.087640000000	3.226946000000	3.827969000000
H	-0.946774000000	7.259928000000	5.288267000000
C	-4.308355000000	1.545893000000	2.577146000000
N	-4.806704000000	0.260091000000	2.362477000000
C	-6.121806000000	0.050001000000	2.035623000000
N	-6.971310000000	1.084298000000	1.842249000000
C	-6.446314000000	2.291995000000	2.080347000000
N	-5.194112000000	2.595498000000	2.448747000000
H	-4.126562000000	-0.531830000000	2.520562000000
H	-5.943352000000	-1.998238000000	2.213843000000
N	-6.548298000000	-1.207613000000	1.888759000000
H	-7.538248000000	-1.336964000000	1.705909000000
O	-3.098845000000	1.692088000000	2.863037000000
H	-7.122301000000	3.139568000000	1.948932000000
C	-3.447783000000	-2.799761000000	3.207915000000
N	-2.648535000000	-3.777575000000	3.810078000000
C	-3.150471000000	-5.022269000000	4.108227000000
N	-4.433428000000	-5.348227000000	3.824422000000
C	-5.146164000000	-4.377075000000	3.249922000000
N	-4.751578000000	-3.139966000000	2.923166000000
H	-1.631068000000	-3.550448000000	3.940335000000
H	-1.326353000000	-5.747727000000	4.786140000000
N	-2.354857000000	-5.921485000000	4.685092000000
H	-2.754450000000	-6.840277000000	4.849395000000
O	-2.961361000000	-1.674691000000	2.953044000000
H	-6.182160000000	-4.628006000000	3.009519000000
C	0.842858000000	-4.198373000000	4.069583000000
N	2.197959000000	-4.122535000000	3.729998000000
C	3.050317000000	-5.173034000000	3.958777000000
N	2.614308000000	-6.323893000000	4.519817000000
C	1.314935000000	-6.338251000000	4.831255000000
N	0.407468000000	-5.370731000000	4.645752000000
H	2.548248000000	-3.211609000000	3.331524000000
H	4.709315000000	-4.192758000000	3.160480000000
N	4.333912000000	-5.059440000000	3.606409000000
H	4.941080000000	-5.835517000000	3.849948000000
O	0.087640000000	-3.226946000000	3.827969000000
H	0.946774000000	-7.259928000000	5.288267000000
C	6.121806000000	-0.050001000000	2.035623000000

N	6.971310000000	-1.084298000000	1.842249000000
C	6.446314000000	-2.291995000000	2.080347000000
N	5.194112000000	-2.595498000000	2.448747000000
H	4.126562000000	0.531830000000	2.520562000000
H	5.943352000000	1.998238000000	2.213843000000
H	7.122301000000	-3.139568000000	1.948932000000
N	6.548298000000	1.207613000000	1.888759000000
H	7.538248000000	1.336964000000	1.705909000000
O	3.098845000000	-1.692088000000	2.863037000000
C	4.308355000000	-1.545893000000	2.577146000000
N	4.806704000000	-0.260091000000	2.362477000000
Rb	0.000000000000	0.000000000000	-2.292291000000
Rb	0.000000000000	0.000000000000	2.433716000000

(C_{2h})	aAM'$_6$-Cs$^+$-[aAM'$_6$]-Cs$^+$-aAM'$_6$		
C	4.162884000000	-2.119604000000	-3.084602000000
N	4.854829000000	-0.900817000000	-3.105726000000
C	6.227596000000	-0.862731000000	-3.201719000000
N	6.962691000000	-1.998134000000	-3.248750000000
C	6.253089000000	-3.127428000000	-3.228078000000
N	4.923969000000	-3.266355000000	-3.154528000000
H	4.284325000000	-0.021083000000	-3.059382000000
H	6.311072000000	1.219167000000	-3.213138000000
N	6.847006000000	0.315667000000	-3.236626000000
H	7.856021000000	0.292355000000	-3.345029000000
O	2.915251000000	-2.131517000000	-2.993886000000
H	6.829793000000	-4.054667000000	-3.268115000000
C	3.915227000000	2.543425000000	-3.085073000000
N	3.205383000000	3.752301000000	-3.103200000000
C	3.858913000000	4.960330000000	-3.196395000000
N	5.209778000000	5.029679000000	-3.242340000000
C	5.833068000000	3.850613000000	-3.224567000000
N	5.288860000000	2.629827000000	-3.155018000000
H	2.158195000000	3.698144000000	-3.059070000000
H	2.097873000000	6.073808000000	-3.210785000000
N	3.148489000000	6.086254000000	-3.230215000000
H	3.673822000000	6.948116000000	-3.338281000000
O	3.302407000000	1.456425000000	-2.997713000000
H	6.924441000000	3.886680000000	-3.264206000000
C	-0.247344000000	4.661228000000	-3.088569000000
N	-1.649128000000	4.652583000000	-3.105875000000
C	-2.367528000000	5.823198000000	-3.198166000000
N	-1.750948000000	7.027063000000	-3.242962000000
C	-0.418178000000	6.975649000000	-3.225854000000
N	0.366142000000	5.893258000000	-3.158449000000
H	-2.126603000000	3.719077000000	-3.059048000000
H	-4.212574000000	4.855180000000	-3.210626000000
N	-3.697750000000	5.770965000000	-3.233320000000
H	-4.182002000000	6.656891000000	-3.338846000000
O	0.385930000000	3.585915000000	-3.001964000000
H	0.097252000000	7.938356000000	-3.264447000000
C	-4.162884000000	2.119604000000	-3.084602000000
N	-4.854829000000	0.900817000000	-3.105726000000
C	-6.227596000000	0.862731000000	-3.201719000000
N	-6.962691000000	1.998134000000	-3.248750000000
C	-6.253089000000	3.127428000000	-3.228078000000
N	-4.923969000000	3.266355000000	-3.154528000000
H	-4.284325000000	0.021083000000	-3.059382000000

H	-6.311072000000	-1.219167000000	-3.213138000000
N	-6.847006000000	-0.315667000000	-3.236626000000
H	-7.856021000000	-0.292355000000	-3.345029000000
O	-2.915251000000	2.131517000000	-2.993886000000
H	-6.829793000000	4.054667000000	-3.268115000000
C	-3.915227000000	-2.543425000000	-3.085073000000
N	-3.205383000000	-3.752301000000	-3.103200000000
C	-3.858913000000	-4.960330000000	-3.196395000000
N	-5.209778000000	-5.029679000000	-3.242340000000
C	-5.833068000000	-3.850613000000	-3.224567000000
N	-5.288860000000	-2.629827000000	-3.155018000000
H	-2.158195000000	-3.698144000000	-3.059070000000
H	-2.097873000000	-6.073808000000	-3.210785000000
N	-3.148489000000	-6.086254000000	-3.230215000000
H	-3.673822000000	-6.948116000000	-3.338281000000
O	-3.302407000000	-1.456425000000	-2.997713000000
H	-6.924441000000	-3.886680000000	-3.264206000000
N	3.697750000000	-5.770965000000	-3.233320000000
H	4.182002000000	-6.656891000000	-3.338846000000
O	-0.385930000000	-3.585915000000	-3.001964000000
C	0.247344000000	-4.661228000000	-3.088569000000
N	1.649128000000	-4.652583000000	-3.105875000000
H	-0.097252000000	-7.938356000000	-3.264447000000
H	4.212574000000	-4.855180000000	-3.210626000000
H	2.126603000000	-3.719077000000	-3.059048000000
N	-0.366142000000	-5.893258000000	-3.158449000000
C	0.418178000000	-6.975649000000	-3.225854000000
N	1.750948000000	-7.027063000000	-3.242962000000
C	2.367528000000	-5.823198000000	-3.198166000000
C	-1.898690000000	-4.194628000000	0.000000000000
N	-0.659468000000	-4.842418000000	0.000000000000
C	-0.572686000000	-6.214302000000	0.000000000000
N	-1.678968000000	-6.995259000000	0.000000000000
C	-2.831709000000	-6.325964000000	0.000000000000
N	-3.016423000000	-5.001641000000	0.000000000000
H	0.195462000000	-4.228837000000	0.000000000000
H	1.504872000000	-6.207324000000	0.000000000000
N	0.627446000000	-6.787841000000	0.000000000000
H	0.651203000000	-7.802012000000	0.000000000000
O	-1.957327000000	-2.945944000000	0.000000000000
H	-3.739106000000	-6.934418000000	0.000000000000
C	2.688119000000	-3.743267000000	0.000000000000
N	3.869118000000	-2.994624000000	0.000000000000
C	5.100202000000	-3.605860000000	0.000000000000
N	5.222957000000	-4.954408000000	0.000000000000
C	4.066466000000	-5.617329000000	0.000000000000
N	2.827344000000	-5.114862000000	0.000000000000
H	3.764913000000	-1.947377000000	0.000000000000
H	6.131710000000	-1.802905000000	0.000000000000
N	6.196747000000	-2.852963000000	0.000000000000
H	7.087248000000	-3.338886000000	0.000000000000
O	1.578032000000	-3.168681000000	0.000000000000
H	4.139290000000	-6.707457000000	0.000000000000
C	4.587095000000	0.452242000000	0.000000000000
N	4.527814000000	1.849190000000	0.000000000000
C	5.671962000000	2.610994000000	0.000000000000
N	6.901476000000	2.043776000000	0.000000000000
C	6.898751000000	0.710762000000	0.000000000000
N	5.844811000000	-0.112138000000	0.000000000000

H	3.568501000000	2.281557000000	0.000000000000
H	4.624367000000	4.404573000000	0.000000000000
N	5.566776000000	3.936870000000	0.000000000000
H	6.432085000000	4.466281000000	0.000000000000
O	3.534765000000	-0.222422000000	0.000000000000
H	7.879720000000	0.229726000000	0.000000000000
C	1.898690000000	4.194628000000	0.000000000000
N	0.659468000000	4.842418000000	0.000000000000
C	0.572686000000	6.214302000000	0.000000000000
N	1.678968000000	6.995259000000	0.000000000000
C	2.831709000000	6.325964000000	0.000000000000
N	3.016423000000	5.001641000000	0.000000000000
H	-0.195462000000	4.228837000000	0.000000000000
H	-1.504872000000	6.207324000000	0.000000000000
N	-0.627446000000	6.787841000000	0.000000000000
H	-0.651203000000	7.802012000000	0.000000000000
O	1.957327000000	2.945944000000	0.000000000000
H	3.739106000000	6.934418000000	0.000000000000
N	-2.827344000000	5.114862000000	0.000000000000
N	-5.222957000000	4.954408000000	0.000000000000
C	-5.100202000000	3.605860000000	0.000000000000
C	-4.066466000000	5.617329000000	0.000000000000
H	-3.764913000000	1.947377000000	0.000000000000
H	-6.131710000000	1.802905000000	0.000000000000
H	-4.139290000000	6.707457000000	0.000000000000
N	-6.196747000000	2.852963000000	0.000000000000
H	-7.087248000000	3.338886000000	0.000000000000
O	-1.578032000000	3.168681000000	0.000000000000
C	-2.688119000000	3.743267000000	0.000000000000
N	-3.869118000000	2.994624000000	0.000000000000
C	-4.587095000000	-0.452242000000	0.000000000000
N	-4.527814000000	-1.849190000000	0.000000000000
C	-5.671962000000	-2.610994000000	0.000000000000
N	-6.901476000000	-2.043776000000	0.000000000000
C	-6.898751000000	-0.710762000000	0.000000000000
N	-5.844811000000	0.112138000000	0.000000000000
H	-3.568501000000	-2.281557000000	0.000000000000
H	-4.624367000000	-4.404573000000	0.000000000000
N	-5.566776000000	-3.936870000000	0.000000000000
H	-6.432085000000	-4.466281000000	0.000000000000
O	-3.534765000000	0.222422000000	0.000000000000
H	-7.879720000000	-0.229726000000	0.000000000000
C	4.162884000000	-2.119604000000	3.084602000000
N	4.854829000000	-0.900817000000	3.105726000000
C	6.227596000000	-0.862731000000	3.201719000000
N	6.962691000000	-1.998134000000	3.248750000000
C	6.253089000000	-3.127428000000	3.228078000000
N	4.923969000000	-3.266355000000	3.154528000000
H	4.284325000000	-0.021083000000	3.059382000000
H	6.311072000000	1.219167000000	3.213138000000
N	6.847006000000	0.315667000000	3.236626000000
H	7.856021000000	0.292355000000	3.345029000000
O	2.915251000000	-2.131517000000	2.993886000000
H	6.829793000000	-4.054667000000	3.268115000000
C	3.915227000000	2.543425000000	3.085073000000
N	3.205383000000	3.752301000000	3.103200000000
C	3.858913000000	4.960330000000	3.196395000000
N	5.209778000000	5.029679000000	3.242340000000
C	5.833068000000	3.850613000000	3.224567000000

N	5.288860000000	2.629827000000	3.155018000000
H	2.158195000000	3.698144000000	3.059070000000
H	2.097873000000	6.073808000000	3.210785000000
N	3.148489000000	6.086254000000	3.230215000000
H	3.673822000000	6.948116000000	3.338281000000
O	3.302407000000	1.456425000000	2.997713000000
H	6.924441000000	3.886680000000	3.264206000000
N	-1.649128000000	4.652583000000	3.105875000000
C	-2.367528000000	5.823198000000	3.198166000000
N	-1.750948000000	7.027063000000	3.242962000000
C	-0.418178000000	6.975649000000	3.225854000000
N	0.366142000000	5.893258000000	3.158449000000
H	-2.126603000000	3.719077000000	3.059048000000
H	-4.212574000000	4.855180000000	3.210626000000
H	0.097252000000	7.938356000000	3.264447000000
N	-3.697750000000	5.770965000000	3.233320000000
H	-4.182002000000	6.656891000000	3.338846000000
O	0.385930000000	3.585915000000	3.001964000000
C	-0.247344000000	4.661228000000	3.088569000000
C	-4.162884000000	2.119604000000	3.084602000000
N	-4.854829000000	0.900817000000	3.105726000000
C	-6.227596000000	0.862731000000	3.201719000000
N	-6.962691000000	1.998134000000	3.248750000000
C	-6.253089000000	3.127428000000	3.228078000000
N	-4.923969000000	3.266355000000	3.154528000000
H	-4.284325000000	0.021083000000	3.059382000000
H	-6.311072000000	-1.219167000000	3.213138000000
N	-6.847006000000	-0.315667000000	3.236626000000
H	-7.856021000000	-0.292355000000	3.345029000000
O	-2.915251000000	2.131517000000	2.993886000000
H	-6.829793000000	4.054667000000	3.268115000000
C	-3.915227000000	-2.543425000000	3.085073000000
N	-3.205383000000	-3.752301000000	3.103200000000
C	-3.858913000000	-4.960330000000	3.196395000000
N	-5.209778000000	-5.029679000000	3.242340000000
C	-5.833068000000	-3.850613000000	3.224567000000
N	-5.288860000000	-2.629827000000	3.155018000000
H	-2.158195000000	-3.698144000000	3.059070000000
H	-2.097873000000	-6.073808000000	3.210785000000
N	-3.148489000000	-6.086254000000	3.230215000000
H	-3.673822000000	-6.948116000000	3.338281000000
O	-3.302407000000	-1.456425000000	2.997713000000
H	-6.924441000000	-3.886680000000	3.264206000000
C	0.247344000000	-4.661228000000	3.088569000000
N	1.649128000000	-4.652583000000	3.105875000000
C	2.367528000000	-5.823198000000	3.198166000000
N	1.750948000000	-7.027063000000	3.242962000000
C	0.418178000000	-6.975649000000	3.225854000000
N	-0.366142000000	-5.893258000000	3.158449000000
H	2.126603000000	-3.719077000000	3.059048000000
H	4.212574000000	-4.855180000000	3.210626000000
N	3.697750000000	-5.770965000000	3.233320000000
H	4.182002000000	-6.656891000000	3.338846000000
O	-0.385930000000	-3.585915000000	3.001964000000
H	-0.097252000000	-7.938356000000	3.264447000000
Cs	0.000000000000	0.000000000000	-2.217151000000
Cs	0.000000000000	0.000000000000	2.217151000000

(C1)	*a*AM'$_6$-Cs$^+$-[*a*AM'$_6$]-Cs$^+$-*a*AM'$_6$		
C	-1.606484000000	-4.195520000000	0.619371000000
N	-0.325645000000	-4.692508000000	0.873761000000
C	-0.118525000000	-6.022154000000	1.136905000000
N	-1.150975000000	-6.889930000000	1.243654000000
C	-2.354985000000	-6.357563000000	1.012872000000
N	-2.652850000000	-5.092146000000	0.698830000000
H	0.474581000000	-4.023738000000	0.735324000000
H	1.946117000000	-5.854353000000	1.029449000000
N	1.131537000000	-6.460267000000	1.298735000000
H	1.246118000000	-7.458462000000	1.442773000000
O	-1.759663000000	-2.985301000000	0.349955000000
H	-3.202429000000	-7.042146000000	1.093324000000
C	2.860769000000	-3.433501000000	0.113037000000
N	3.899559000000	-2.662191000000	-0.417661000000
C	5.154943000000	-3.183517000000	-0.601403000000
N	5.432944000000	-4.474787000000	-0.308290000000
C	4.405779000000	-5.165975000000	0.192243000000
N	3.158863000000	-4.742682000000	0.426134000000
H	3.696588000000	-1.642376000000	-0.581395000000
H	5.972553000000	-1.363536000000	-1.191393000000
N	6.117721000000	-2.397061000000	-1.085693000000
H	7.046058000000	-2.802514000000	-1.147327000000
O	1.727762000000	-2.929376000000	0.269187000000
H	4.609762000000	-6.209430000000	0.442469000000
C	5.346644000000	3.004035000000	-0.517219000000
N	6.536053000000	2.568952000000	-0.996483000000
C	6.579668000000	1.265814000000	-1.284142000000
N	5.603948000000	0.358214000000	-1.161474000000
H	3.344259000000	2.486531000000	-0.016104000000
H	4.277943000000	4.658338000000	0.145489000000
H	7.531806000000	0.893767000000	-1.668445000000
N	5.193082000000	4.292659000000	-0.213428000000
O	3.402215000000	0.041809000000	-0.516304000000
C	4.388859000000	0.793232000000	-0.675837000000
N	4.289535000000	2.146724000000	-0.340621000000
H	5.963133000000	4.910612000000	-0.448746000000
C	1.606484000000	4.195520000000	0.619371000000
N	0.325645000000	4.692508000000	0.873761000000
C	0.118525000000	6.022154000000	1.136905000000
N	1.150975000000	6.889930000000	1.243654000000
C	2.354985000000	6.357563000000	1.012872000000
N	2.652850000000	5.092146000000	0.698830000000
H	-0.474581000000	4.023738000000	0.735324000000
H	-1.946117000000	5.854353000000	1.029449000000
N	-1.131537000000	6.460267000000	1.298735000000
H	-1.246118000000	7.458462000000	1.442773000000
O	1.759663000000	2.985301000000	0.349955000000
H	3.202429000000	7.042146000000	1.093324000000
C	-2.860769000000	3.433501000000	0.113037000000
N	-3.899559000000	2.662191000000	-0.417661000000
C	-5.154943000000	3.183517000000	-0.601403000000
N	-5.432944000000	4.474787000000	-0.308290000000
C	-4.405779000000	5.165975000000	0.192243000000
N	-3.158863000000	4.742682000000	0.426134000000
H	-3.696588000000	1.642376000000	-0.581395000000
H	-5.972553000000	1.363536000000	-1.191393000000
N	-6.117721000000	2.397061000000	-1.085693000000

H	-7.046058000000	2.802514000000	-1.147327000000
O	-1.727762000000	2.929376000000	0.269187000000
H	-4.609762000000	6.209430000000	0.442469000000
C	-4.388859000000	-0.793232000000	-0.675837000000
N	-4.289535000000	-2.146724000000	-0.340621000000
C	-5.346644000000	-3.004035000000	-0.517219000000
N	-6.536053000000	-2.568952000000	-0.996483000000
C	-6.579668000000	-1.265814000000	-1.284142000000
N	-5.603948000000	-0.358214000000	-1.161474000000
H	-3.344259000000	-2.486531000000	-0.016104000000
H	-4.277943000000	-4.658338000000	0.145489000000
N	-5.193082000000	-4.292659000000	-0.213428000000
H	-5.963133000000	-4.910612000000	-0.448746000000
O	-3.402215000000	-0.041809000000	-0.516304000000
H	-7.531806000000	-0.893767000000	-1.668445000000
C	-5.809050000000	0.745048000000	-4.391104000000
N	-6.527119000000	1.871171000000	-4.610535000000
C	-5.872815000000	3.010330000000	-4.375127000000
N	-4.616152000000	3.168251000000	-3.939977000000
H	-3.960163000000	-0.074681000000	-3.775821000000
H	-5.875917000000	-1.344266000000	-4.406031000000
H	-6.438881000000	3.928360000000	-4.550817000000
N	-6.380824000000	-0.443219000000	-4.589494000000
H	-7.326906000000	-0.430862000000	-4.957968000000
O	-2.703871000000	2.050616000000	-3.272153000000
C	-3.879734000000	2.028530000000	-3.705251000000
N	-4.505453000000	0.802508000000	-3.959866000000
C	-3.671458000000	-2.646848000000	-3.467409000000
N	-3.038206000000	-3.803759000000	-2.991915000000
C	-3.682343000000	-5.015429000000	-2.983297000000
N	-4.959207000000	-5.136943000000	-3.412069000000
C	-5.511852000000	-4.005445000000	-3.860763000000
N	-4.962021000000	-2.785631000000	-3.924137000000
H	-2.032666000000	-3.711922000000	-2.690696000000
H	-2.028615000000	-6.052457000000	-2.259126000000
N	-3.041021000000	-6.090407000000	-2.515135000000
H	-3.525371000000	-6.977888000000	-2.607149000000
O	-3.064736000000	-1.552213000000	-3.437298000000
H	-6.542307000000	-4.087158000000	-4.212605000000
C	0.304273000000	-4.610088000000	-2.395013000000
N	1.673747000000	-4.581584000000	-2.673875000000
C	2.434274000000	-5.725135000000	-2.623164000000
N	1.902657000000	-6.905257000000	-2.228944000000
C	0.590940000000	-6.874370000000	-1.977445000000
N	-0.244297000000	-5.828154000000	-2.048764000000
H	2.083756000000	-3.649241000000	-2.937717000000
H	4.123304000000	-4.769558000000	-3.350081000000
N	3.721985000000	-5.654762000000	-2.957844000000
H	4.250417000000	-6.520925000000	-2.936819000000
O	-0.359779000000	-3.552815000000	-2.467264000000
H	0.142192000000	-7.821620000000	-1.669566000000
C	3.879734000000	-2.028530000000	-3.705251000000
N	4.505453000000	-0.802508000000	-3.959866000000
C	5.809050000000	-0.745048000000	-4.391104000000
N	6.527119000000	-1.871171000000	-4.610535000000
C	5.872815000000	-3.010330000000	-4.375127000000
N	4.616152000000	-3.168251000000	-3.939977000000
H	3.960163000000	0.074681000000	-3.775821000000
H	5.875917000000	1.344266000000	-4.406031000000

N	6.380824000000	0.443219000000	-4.589494000000
H	7.326906000000	0.430862000000	-4.957968000000
O	2.703871000000	-2.050616000000	-3.272153000000
H	6.438881000000	-3.928360000000	-4.550817000000
C	3.671458000000	2.646848000000	-3.467409000000
N	3.038206000000	3.803759000000	-2.991915000000
C	3.682343000000	5.015429000000	-2.983297000000
N	4.959207000000	5.136943000000	-3.412069000000
C	5.511852000000	4.005445000000	-3.860763000000
N	4.962021000000	2.785631000000	-3.924137000000
H	2.032666000000	3.711922000000	-2.690696000000
H	2.028615000000	6.052457000000	-2.259126000000
N	3.041021000000	6.090407000000	-2.515135000000
H	3.525371000000	6.977888000000	-2.607149000000
O	3.064736000000	1.552213000000	-3.437298000000
H	6.542307000000	4.087158000000	-4.212605000000
C	-0.304273000000	4.610088000000	-2.395013000000
N	-1.673747000000	4.581584000000	-2.673875000000
C	-2.434274000000	5.725135000000	-2.623164000000
N	-1.902657000000	6.905257000000	-2.228944000000
C	-0.590940000000	6.874370000000	-1.977445000000
N	0.244297000000	5.828154000000	-2.048764000000
H	-2.083756000000	3.649241000000	-2.937717000000
H	-4.123304000000	4.769558000000	-3.350081000000
N	-3.721985000000	5.654762000000	-2.957844000000
H	-4.250417000000	6.520925000000	-2.936819000000
O	0.359779000000	3.552815000000	-2.467264000000
H	-0.142192000000	7.821620000000	-1.669566000000
C	3.472866000000	2.836165000000	3.146008000000
N	2.658611000000	3.838028000000	3.688563000000
C	3.153888000000	5.094952000000	3.946920000000
N	4.442470000000	5.411949000000	3.677855000000
C	5.169030000000	4.419214000000	3.161801000000
N	4.782372000000	3.168612000000	2.879914000000
H	1.639233000000	3.619476000000	3.812172000000
H	1.318000000000	5.848528000000	4.575251000000
N	2.347599000000	6.017561000000	4.468219000000
H	2.747854000000	6.940213000000	4.607379000000
O	2.996755000000	1.699861000000	2.925538000000
H	6.210237000000	4.660784000000	2.934950000000
C	-0.851913000000	4.292765000000	3.949812000000
N	-2.215346000000	4.197072000000	3.646587000000
C	-3.068891000000	5.250077000000	3.862558000000
N	-2.625850000000	6.422169000000	4.372295000000
C	-1.320415000000	6.453124000000	4.655197000000
N	-0.412509000000	5.484162000000	4.481638000000
H	-2.571387000000	3.273833000000	3.286330000000
H	-4.748155000000	4.231563000000	3.154460000000
N	-4.360662000000	5.118328000000	3.549598000000
H	-4.964416000000	5.899695000000	3.784976000000
O	-0.094322000000	3.321619000000	3.718082000000
H	-0.947391000000	7.390902000000	5.073950000000
C	-4.365861000000	1.573889000000	2.631831000000
N	-4.864530000000	0.281611000000	2.451213000000
C	-6.190180000000	0.061912000000	2.170995000000
N	-7.054946000000	1.089835000000	2.014482000000
C	-6.528692000000	2.302230000000	2.220297000000
N	-5.263119000000	2.616084000000	2.528251000000
H	-4.176663000000	-0.507542000000	2.572364000000

H	-5.987304000000	-1.993455000000	2.298362000000
N	-6.614157000000	-1.196472000000	2.032338000000
H	-7.608667000000	-1.329682000000	1.879793000000
O	-3.147433000000	1.733918000000	2.866234000000
H	-7.215992000000	3.144360000000	2.114527000000
C	-3.472866000000	-2.836165000000	3.146008000000
N	-2.658611000000	-3.838028000000	3.688563000000
C	-3.153888000000	-5.094952000000	3.946920000000
N	-4.442470000000	-5.411949000000	3.677855000000
C	-5.169030000000	-4.419214000000	3.161801000000
N	-4.782372000000	-3.168612000000	2.879914000000
H	-1.639233000000	-3.619476000000	3.812172000000
H	-1.318000000000	-5.848528000000	4.575251000000
N	-2.347599000000	-6.017561000000	4.468219000000
H	-2.747854000000	-6.940213000000	4.607379000000
O	-2.996755000000	-1.699861000000	2.925538000000
H	-6.210237000000	-4.660784000000	2.934950000000
C	0.851913000000	-4.292765000000	3.949812000000
N	2.215346000000	-4.197072000000	3.646587000000
C	3.068891000000	-5.250077000000	3.862558000000
N	2.625850000000	-6.422169000000	4.372295000000
C	1.320415000000	-6.453124000000	4.655197000000
N	0.412509000000	-5.484162000000	4.481638000000
H	2.571387000000	-3.273833000000	3.286330000000
H	4.748155000000	-4.231563000000	3.154460000000
N	4.360662000000	-5.118328000000	3.549598000000
H	4.964416000000	-5.899695000000	3.784976000000
O	0.094322000000	-3.321619000000	3.718082000000
H	0.947391000000	-7.390902000000	5.073950000000
C	6.190180000000	-0.061912000000	2.170995000000
N	7.054946000000	-1.089835000000	2.014482000000
C	6.528692000000	-2.302230000000	2.220297000000
N	5.263119000000	-2.616084000000	2.528251000000
H	4.176663000000	0.507542000000	2.572364000000
H	5.987304000000	1.993455000000	2.298362000000
H	7.215992000000	-3.144360000000	2.114527000000
N	6.614157000000	1.196472000000	2.032338000000
H	7.608667000000	1.329682000000	1.879793000000
O	3.147433000000	-1.733918000000	2.866234000000
C	4.365861000000	-1.573889000000	2.631831000000
N	4.864530000000	-0.281611000000	2.451213000000
Cs	0.000000000000	0.000000000000	-2.225382000000
Cs	0.000000000000	0.000000000000	2.365277000000

*b*AM’			
N	1.47370459	4.41981462	-5.40375510
C	0.75632840	3.48383552	-5.94484505
N	0.51996904	2.25742074	-5.38492482
C	1.11680744	1.98273688	-4.17874614
N	1.86710094	2.88488142	-3.55805456
C	2.01330382	4.14452815	-4.13288541
H	-0.14746889	1.55690031	-5.82789055
H	0.23658972	0.08745224	-4.06995912
O	2.62387834	5.04383091	-3.51988701
N	0.92134175	0.75585215	-3.65671188
H	0.28798967	3.64595076	-6.91564753
H	1.26616684	0.60447254	-2.71896936

(C_S)	*b*AM’$_2$		
C	-1.991195	4.377997	0.000000
N	-3.222669	5.020368	0.000000
C	-3.210266	6.403484	0.000000
N	-2.144052	7.130778	0.000000
C	-0.871852	6.437017	0.000000
N	-0.853255	5.022944	0.000000
H	-4.107699	4.490593	0.000000
O	0.169985	7.086107	0.000000
H	-1.119440	2.547261	0.000000
N	-2.020268	3.009480	0.000000
H	-2.891466	2.477448	0.000000
H	-4.200293	6.868746	0.000000
C	-7.234950	0.453601	0.000000
N	-7.132434	1.735784	0.000000
C	-5.810745	2.288852	0.000000
N	-4.690585	1.435971	0.000000
H	-6.299297	-1.421134	0.000000
H	-8.214302	-0.031212	0.000000
O	-5.670059	3.513227	0.000000
H	-2.880907	-0.316619	0.000000
N	-3.812955	-0.714829	0.000000
H	-3.912998	-1.721677	0.000000
C	-4.872584	0.138419	0.000000
N	-6.148048	-0.415644	0.000000

(C_{2h})	*b*AM’$_6$		
C	-2.61845300	-4.01138300	0.00000000
N	-2.68282900	-5.38987500	0.00000000
C	-3.93888300	-5.95795700	0.00000000
N	-5.06148200	-5.30055900	0.00000000
C	-4.97017900	-3.88634600	0.00000000
N	-3.72200500	-3.27222900	0.00000000
H	-1.79469500	-5.96082300	0.00000000
O	-6.00554400	-3.18490900	0.00000000
H	-1.38973800	-2.42026800	0.00000000
N	-1.40495400	-3.43187000	0.00000000
H	-0.50508700	-3.98220900	0.00000000
H	-3.94870400	-7.04957900	0.00000000
C	-7.12792400	0.43477300	0.00000000
N	-7.11957900	1.73573100	0.00000000
C	-5.84928900	2.36371000	0.00000000
N	-4.69344200	1.58959900	0.00000000
H	-6.05750400	-1.42378900	0.00000000
H	-8.07842200	-0.10225900	0.00000000
O	-5.75972000	3.61114400	0.00000000
H	-2.79011200	-0.00593700	0.00000000
N	-3.67418100	-0.49776800	0.00000000
H	-3.70067600	-1.55234100	0.00000000
C	-4.78216600	0.26427800	0.00000000
N	-6.00806300	-0.36913500	0.00000000
C	2.16309800	-4.27884600	0.00000000
N	3.32512400	-5.02295900	0.00000000
C	3.18992500	-6.39497300	0.00000000
N	2.05968900	-7.03901500	0.00000000
C	0.87980200	-6.25361900	0.00000000
N	0.97141100	-4.86558000	0.00000000

H	4.26275800	-4.53768300	0.00000000
O	-0.24477900	-6.80072100	0.00000000
H	1.39954000	-2.41901600	0.00000000
N	2.26783500	-2.93819900	0.00000000
H	3.19360000	-2.43296800	0.00000000
H	4.13082800	-6.94862100	0.00000000
C	4.78216600	-0.26427800	0.00000000
N	6.00806300	0.36913500	0.00000000
C	7.12792400	-0.43477300	0.00000000
N	7.11957900	-1.73573100	0.00000000
C	5.84928900	-2.36371000	0.00000000
N	4.69344200	-1.58959900	0.00000000
H	6.05750400	1.42378900	0.00000000
O	5.75972000	-3.61114400	0.00000000
H	2.79011200	0.00593700	0.00000000
N	3.67418100	0.49776800	0.00000000
H	3.70067600	1.55234100	0.00000000
H	8.07842200	0.10225900	0.00000000
C	2.61845300	4.01138300	0.00000000
N	2.68282900	5.38987500	0.00000000
C	3.93888300	5.95795700	0.00000000
N	5.06148200	5.30055900	0.00000000
C	4.97017900	3.88634600	0.00000000
N	3.72200500	3.27222900	0.00000000
H	1.79469500	5.96082300	0.00000000
O	6.00554400	3.18490900	0.00000000
H	1.38973800	2.42026800	0.00000000
N	1.40495400	3.43187000	0.00000000
H	0.50508700	3.98220900	0.00000000
H	3.94870400	7.04957900	0.00000000
C	-2.16309800	4.27884600	0.00000000
N	-3.32512400	5.02295900	0.00000000
C	-3.18992500	6.39497300	0.00000000
N	-2.05968900	7.03901500	0.00000000
C	-0.87980200	6.25361900	0.00000000
N	-0.97141100	4.86558000	0.00000000
H	-4.26275800	4.53768300	0.00000000
O	0.24477900	6.80072100	0.00000000
H	-1.39954000	2.41901600	0.00000000
N	-2.26783500	2.93819900	0.00000000
H	-3.19360000	2.43296800	0.00000000
H	-4.13082800	6.94862100	0.00000000

(C_1) *b*AM’$_6$

C	6.49774000	2.03424000	-0.85332100
N	5.76487000	1.09031000	-0.18026500
C	4.61496600	1.50765400	0.45181800
N	4.19897200	2.76916300	0.37559200
C	4.94947100	3.67385600	-0.37828000
N	6.16992300	3.28779600	-0.97160400
H	6.07089100	0.06616000	-0.13790900
H	4.09745200	-0.43232900	0.99307300
O	4.53588300	4.84545800	-0.54300500
N	3.94134000	0.58352400	1.17720500
H	7.41852000	1.66572700	-1.30960900
H	3.05824400	0.88371500	1.57279100
C	1.43115100	6.54704300	-1.10314000
N	1.94257400	5.49843700	-0.38139300

C	1.05744300	4.73342900	0.34473600
N	-0.25177300	4.97018800	0.31487200
C	-0.72153900	6.03206300	-0.45981200
N	0.17134300	6.86593300	-1.16659900
H	2.98446700	5.26262600	-0.39032900
H	2.54757400	3.39767000	0.95072600
O	-1.95325900	6.24652600	-0.54536200
N	1.57419000	3.74188700	1.10450800
H	2.17070700	7.13577000	-1.64929700
H	0.91688700	3.19289500	1.64506700
N	-6.07660000	3.71870900	-0.47096600
C	-5.14583100	4.62723900	-0.44797100
N	-3.84989800	4.41686600	-0.04800700
C	-3.51487400	3.15369800	0.38549900
N	-4.40328700	2.16321900	0.39143700
C	-5.69028500	2.41801300	-0.08380100
H	-3.10004200	5.16925900	-0.15844700
H	-1.51537200	3.69203700	0.69783800
O	-6.51975800	1.48258500	-0.17394900
N	-2.25102600	2.96145800	0.82732500
H	-5.37160600	5.64722000	-0.76475600
H	-1.97409700	2.00013700	0.98533500
N	-4.19897200	-2.76916300	0.37559200
C	-6.49774000	-2.03424000	-0.85332100
N	-5.76487000	-1.09031000	-0.18026500
H	-7.41852000	-1.66572700	-1.30960900
H	-3.05824400	-0.88371500	1.57279100
C	-4.61496600	-1.50765400	0.45181800
C	-4.94947100	-3.67385600	-0.37828000
N	-6.16992300	-3.28779600	-0.97160400
H	-6.07089100	-0.06616000	-0.13790900
H	-4.09745200	0.43232900	0.99307300
O	-4.53588300	-4.84545800	-0.54300500
N	-3.94134000	-0.58352400	1.17720500
C	-1.43115100	-6.54704300	-1.10314000
N	-1.94257400	-5.49843700	-0.38139300
C	-1.05744300	-4.73342900	0.34473600
N	0.25177300	-4.97018800	0.31487200
C	0.72153900	-6.03206300	-0.45981200
N	-0.17134300	-6.86593300	-1.16659900
H	-2.98446700	-5.26262600	-0.39032900
H	-2.54757400	-3.39767000	0.95072600
O	1.95325900	-6.24652600	-0.54536200
N	-1.57419000	-3.74188700	1.10450800
H	-2.17070700	-7.13577000	-1.64929700
H	-0.91688700	-3.19289500	1.64506700
N	6.07660000	-3.71870900	-0.47096600
C	5.14583100	-4.62723900	-0.44797100
N	3.84989800	-4.41686600	-0.04800700
C	3.51487400	-3.15369800	0.38549900
N	4.40328700	-2.16321900	0.39143700
C	5.69028500	-2.41801300	-0.08380100
H	3.10004200	-5.16925900	-0.15844700
H	1.51537200	-3.69203700	0.69783800
O	6.51975800	-1.48258500	-0.17394900
N	2.25102600	-2.96145800	0.82732500
H	5.37160600	-5.64722000	-0.76475600
H	1.97409700	-2.00013700	0.98533500

(C_{2h})	bAM'$_6$-[bAM'$_6$]-bAM'$_6$		
C	-2.448283000000	-4.197400000000	0.000000000000
N	-2.468043000000	-5.568458000000	0.000000000000
C	-3.696578000000	-6.191870000000	0.000000000000
N	-4.838483000000	-5.569404000000	0.000000000000
C	-4.792999000000	-4.167836000000	0.000000000000
N	-3.577941000000	-3.495624000000	0.000000000000
H	-1.567743000000	-6.099466000000	0.000000000000
C	-7.219258000000	0.074637000000	0.000000000000
O	-5.858351000000	-3.500466000000	0.000000000000
H	-1.297545000000	-2.555511000000	0.000000000000
N	-1.258077000000	-3.566407000000	0.000000000000
H	-0.325235000000	-4.052318000000	0.000000000000
H	-3.658291000000	-7.279001000000	0.000000000000
N	-7.254904000000	1.374538000000	0.000000000000
C	2.434030000000	-4.147463000000	0.000000000000
N	3.615042000000	-4.851936000000	0.000000000000
C	3.538505000000	-6.228446000000	0.000000000000
N	2.428243000000	-6.904596000000	0.000000000000
C	1.236851000000	-6.161205000000	0.000000000000
N	1.261703000000	-4.774574000000	0.000000000000
H	4.531052000000	-4.336936000000	0.000000000000
C	-6.018717000000	2.039424000000	0.000000000000
O	0.127936000000	-6.751918000000	0.000000000000
H	1.597037000000	-2.326602000000	0.000000000000
N	2.488387000000	-2.804730000000	0.000000000000
H	3.388010000000	-2.252826000000	0.000000000000
H	4.498195000000	-6.742162000000	0.000000000000
N	-4.829652000000	1.323945000000	0.000000000000
C	4.868190000000	0.005360000000	0.000000000000
N	6.065169000000	0.677778000000	0.000000000000
C	7.219258000000	-0.074637000000	0.000000000000
N	7.254904000000	-1.374538000000	0.000000000000
C	6.018717000000	-2.039424000000	0.000000000000
N	4.829652000000	-1.323945000000	0.000000000000
H	6.073887000000	1.727247000000	0.000000000000
H	-6.073887000000	-1.727247000000	0.000000000000
O	5.975007000000	-3.295597000000	0.000000000000
H	2.870720000000	0.181777000000	0.000000000000
N	3.727970000000	0.718122000000	0.000000000000
H	3.690603000000	1.771109000000	0.000000000000
H	8.142541000000	0.501868000000	0.000000000000
H	-8.142541000000	-0.501868000000	0.000000000000
C	2.448283000000	4.197400000000	0.000000000000
N	2.468043000000	5.568458000000	0.000000000000
C	3.696578000000	6.191870000000	0.000000000000
N	4.838483000000	5.569404000000	0.000000000000
C	4.792999000000	4.167836000000	0.000000000000
N	3.577941000000	3.495624000000	0.000000000000
H	1.567743000000	6.099466000000	0.000000000000
O	-5.975007000000	3.295597000000	0.000000000000
O	5.858351000000	3.500466000000	0.000000000000
H	1.297545000000	2.555511000000	0.000000000000
N	1.258077000000	3.566407000000	0.000000000000
H	0.325235000000	4.052318000000	0.000000000000
H	3.658291000000	7.279001000000	0.000000000000
H	-2.870720000000	-0.181777000000	0.000000000000
C	-2.434030000000	4.147463000000	0.000000000000

N	-3.615042000000	4.851936000000	0.000000000000
C	-3.538505000000	6.228446000000	0.000000000000
N	-2.428243000000	6.904596000000	0.000000000000
C	-1.236851000000	6.161205000000	0.000000000000
N	-1.261703000000	4.774574000000	0.000000000000
H	-4.531052000000	4.336936000000	0.000000000000
N	-3.727970000000	-0.718122000000	0.000000000000
O	-0.127936000000	6.751918000000	0.000000000000
H	-1.597037000000	2.326602000000	0.000000000000
N	-2.488387000000	2.804730000000	0.000000000000
H	-3.388010000000	2.252826000000	0.000000000000
H	-4.498195000000	6.742162000000	0.000000000000
H	-3.690603000000	-1.771109000000	0.000000000000
C	-4.868190000000	-0.005360000000	0.000000000000
N	-6.065169000000	-0.677778000000	0.000000000000
C	3.841211000000	-2.660776000000	-3.246348000000
N	5.202561000000	-2.679682000000	-3.056873000000
C	5.799947000000	-3.903793000000	-2.865035000000
N	5.175505000000	-5.045738000000	-2.880086000000
C	3.783864000000	-5.002195000000	-3.107501000000
N	3.134542000000	-3.784955000000	-3.297831000000
H	5.748345000000	-1.772465000000	-3.038421000000
C	-0.433298000000	-7.094186000000	-2.747126000000
O	3.119467000000	-6.063646000000	-3.110550000000
H	2.231826000000	-1.485573000000	-3.518262000000
N	3.231590000000	-1.463274000000	-3.361915000000
H	3.759272000000	-0.556031000000	-3.379679000000
H	6.872268000000	-3.868996000000	-2.674922000000
N	-1.735250000000	-7.059048000000	-2.719273000000
C	4.115323000000	2.168329000000	-3.336172000000
N	4.860989000000	3.297824000000	-3.101140000000
C	6.205916000000	3.133387000000	-2.864701000000
N	6.823669000000	1.987578000000	-2.861139000000
C	6.033814000000	0.842948000000	-3.087849000000
N	4.670364000000	0.962215000000	-3.347631000000
H	4.394441000000	4.250991000000	-3.093598000000
C	-2.342426000000	-5.869967000000	-3.167346000000
O	6.555607000000	-0.293878000000	-3.022951000000
H	2.316279000000	1.502995000000	-3.920039000000
N	2.780109000000	2.310632000000	-3.519680000000
H	2.357733000000	3.256667000000	-3.686404000000
H	6.754790000000	4.051585000000	-2.658676000000
N	-1.584111000000	-4.860729000000	-3.754273000000
C	0.260270000000	4.982194000000	-3.767772000000
N	-0.364897000000	6.084311000000	-3.229502000000
C	0.433298000000	7.094186000000	-2.747126000000
N	1.735250000000	7.059048000000	-2.719273000000
C	2.342426000000	5.869967000000	-3.167346000000
N	1.584111000000	4.860729000000	-3.754273000000
H	-1.423357000000	6.128450000000	-3.196877000000
H	1.423357000000	-6.128450000000	-3.196877000000
O	3.575058000000	5.706990000000	-3.005694000000
H	-0.028324000000	3.236100000000	-4.724748000000
N	-0.518203000000	4.032727000000	-4.334070000000
H	-1.514947000000	3.926468000000	-4.027940000000
H	-0.098519000000	7.961343000000	-2.358778000000
H	0.098519000000	-7.961343000000	-2.358778000000
C	-3.841211000000	2.660776000000	-3.246348000000
N	-5.202561000000	2.679682000000	-3.056873000000

C	-5.799947000000	3.903793000000	-2.865035000000
N	-5.175505000000	5.045738000000	-2.880086000000
C	-3.783864000000	5.002195000000	-3.107501000000
N	-3.134542000000	3.784955000000	-3.297831000000
H	-5.748345000000	1.772465000000	-3.038421000000
O	-3.575058000000	-5.706990000000	-3.005694000000
O	-3.119467000000	6.063646000000	-3.110550000000
H	-2.231826000000	1.485573000000	-3.518262000000
N	-3.231590000000	1.463274000000	-3.361915000000
H	-3.759272000000	0.556031000000	-3.379679000000
H	-6.872268000000	3.868996000000	-2.674922000000
H	0.028324000000	-3.236100000000	-4.724748000000
C	-4.115323000000	-2.168329000000	-3.336172000000
N	-4.860989000000	-3.297824000000	-3.101140000000
C	-6.205916000000	-3.133387000000	-2.864701000000
N	-6.823669000000	-1.987578000000	-2.861139000000
C	-6.033814000000	-0.842948000000	-3.087849000000
N	-4.670364000000	-0.962215000000	-3.347631000000
H	-4.394441000000	-4.250991000000	-3.093598000000
N	0.518203000000	-4.032727000000	-4.334070000000
O	-6.555607000000	0.293878000000	-3.022951000000
H	-2.316279000000	-1.502995000000	-3.920039000000
N	-2.780109000000	-2.310632000000	-3.519680000000
H	-2.357733000000	-3.256667000000	-3.686404000000
H	-6.754790000000	-4.051585000000	-2.658676000000
H	1.514947000000	-3.926468000000	-4.027940000000
C	-0.260270000000	-4.982194000000	-3.767772000000
N	0.364897000000	-6.084311000000	-3.229502000000
C	3.841211000000	-2.660776000000	3.246348000000
N	5.202561000000	-2.679682000000	3.056873000000
C	5.799947000000	-3.903793000000	2.865035000000
N	5.175505000000	-5.045738000000	2.880086000000
C	3.783864000000	-5.002195000000	3.107501000000
N	3.134542000000	-3.784955000000	3.297831000000
H	5.748345000000	-1.772465000000	3.038421000000
C	-0.433298000000	-7.094186000000	2.747126000000
O	3.119467000000	-6.063646000000	3.110550000000
H	2.231826000000	-1.485573000000	3.518262000000
N	3.231590000000	-1.463274000000	3.361915000000
H	3.759272000000	-0.556031000000	3.379679000000
H	6.872268000000	-3.868996000000	2.674922000000
N	-1.735250000000	-7.059048000000	2.719273000000
C	4.115323000000	2.168329000000	3.336172000000
N	4.860989000000	3.297824000000	3.101140000000
C	6.205916000000	3.133387000000	2.864701000000
N	6.823669000000	1.987578000000	2.861139000000
C	6.033814000000	0.842948000000	3.087849000000
N	4.670364000000	0.962215000000	3.347631000000
H	4.394441000000	4.250991000000	3.093598000000
C	-2.342426000000	-5.869967000000	3.167346000000
O	6.555607000000	-0.293878000000	3.022951000000
H	2.316279000000	1.502995000000	3.920039000000
N	2.780109000000	2.310632000000	3.519680000000
H	2.357733000000	3.256667000000	3.686404000000
H	6.754790000000	4.051585000000	2.658676000000
N	-1.584111000000	-4.860729000000	3.754273000000
C	0.260270000000	4.982194000000	3.767772000000
N	-0.364897000000	6.084311000000	3.229502000000
C	0.433298000000	7.094186000000	2.747126000000

N	1.735250000000	7.059048000000	2.719273000000
C	2.342426000000	5.869967000000	3.167346000000
N	1.584111000000	4.860729000000	3.754273000000
H	-1.423357000000	6.128450000000	3.196877000000
H	1.423357000000	-6.128450000000	3.196877000000
O	3.575058000000	5.706990000000	3.005694000000
H	-0.028324000000	3.236100000000	4.724748000000
N	-0.518203000000	4.032727000000	4.334070000000
H	-1.514947000000	3.926468000000	4.027940000000
H	-0.098519000000	7.961343000000	2.358778000000
H	0.098519000000	-7.961343000000	2.358778000000
C	-3.841211000000	2.660776000000	3.246348000000
N	-5.202561000000	2.679682000000	3.056873000000
C	-5.799947000000	3.903793000000	2.865035000000
N	-5.175505000000	5.045738000000	2.880086000000
C	-3.783864000000	5.002195000000	3.107501000000
N	-3.134542000000	3.784955000000	3.297831000000
H	-5.748345000000	1.772465000000	3.038421000000
O	-3.575058000000	-5.706990000000	3.005694000000
O	-3.119467000000	6.063646000000	3.110550000000
H	-2.231826000000	1.485573000000	3.518262000000
N	-3.231590000000	1.463274000000	3.361915000000
H	-3.759272000000	0.556031000000	3.379679000000
H	-6.872268000000	3.868996000000	2.674922000000
H	0.028324000000	-3.236100000000	4.724748000000
C	-4.115323000000	-2.168329000000	3.336172000000
N	-4.860989000000	-3.297824000000	3.101140000000
C	-6.205916000000	-3.133387000000	2.864701000000
N	-6.823669000000	-1.987578000000	2.861139000000
C	-6.033814000000	-0.842948000000	3.087849000000
N	-4.670364000000	-0.962215000000	3.347631000000
H	-4.394441000000	-4.250991000000	3.093598000000
N	0.518203000000	-4.032727000000	4.334070000000
O	-6.555607000000	0.293878000000	3.022951000000
H	-2.316279000000	-1.502995000000	3.920039000000
N	-2.780109000000	-2.310632000000	3.519680000000
H	-2.357733000000	-3.256667000000	3.686404000000
H	-6.754790000000	-4.051585000000	2.658676000000
H	1.514947000000	-3.926468000000	4.027940000000
C	-0.260270000000	-4.982194000000	3.767772000000
N	0.364897000000	-6.084311000000	3.229502000000

(C_1) *b*AM'$_6$-[*b*AM'$_6$]-*b*AM'$_6$

C	-2.468667000000	-4.100790000000	-0.338800000000
N	-2.427402000000	-5.402055000000	0.092041000000
C	-3.623972000000	-6.017169000000	0.390366000000
N	-4.788346000000	-5.441523000000	0.305609000000
C	-4.798919000000	-4.099553000000	-0.103939000000
N	-3.617765000000	-3.438882000000	-0.419539000000
H	-1.512735000000	-5.923334000000	0.094603000000
C	-7.174414000000	0.099605000000	-0.170769000000
O	-5.883483000000	-3.468915000000	-0.163301000000
H	-1.433434000000	-2.679775000000	-1.289722000000
N	-1.314024000000	-3.476289000000	-0.670677000000
H	-0.389089000000	-3.960068000000	-0.626127000000
H	-3.538092000000	-7.046251000000	0.732685000000
N	-7.204830000000	1.397463000000	-0.087414000000
C	2.411337000000	-4.087492000000	-0.233708000000

N	3.545078000000	-4.754865000000	0.164711000000
C	3.433454000000	-6.095088000000	0.447872000000
N	2.317811000000	-6.763949000000	0.404563000000
C	1.169020000000	-6.046170000000	0.044806000000
N	1.234833000000	-4.702637000000	-0.300464000000
H	4.471250000000	-4.250331000000	0.187018000000
C	-5.971117000000	2.044430000000	0.069700000000
O	0.053228000000	-6.627315000000	0.044229000000
H	1.664305000000	-2.313545000000	-0.797379000000
N	2.534742000000	-2.790664000000	-0.593587000000
H	3.364864000000	-2.224613000000	-0.284838000000
H	4.359321000000	-6.584149000000	0.744952000000
N	-4.790894000000	1.321311000000	0.177223000000
C	4.841041000000	0.006898000000	0.118823000000
N	6.029860000000	0.660661000000	-0.092535000000
C	7.174012000000	-0.100356000000	-0.158978000000
N	7.204115000000	-1.397899000000	-0.070715000000
C	5.970142000000	-2.044063000000	0.087524000000
N	4.789838000000	-1.320393000000	0.190660000000
H	6.052656000000	1.710227000000	-0.135550000000
H	-6.052851000000	-1.710719000000	-0.139130000000
O	5.920858000000	-3.300513000000	0.133646000000
H	2.873446000000	0.220742000000	0.463804000000
N	3.722075000000	0.741066000000	0.277449000000
H	3.666784000000	1.744558000000	-0.025248000000
H	8.093341000000	0.463590000000	-0.305311000000
H	-8.093498000000	-0.465042000000	-0.315946000000
C	2.468809000000	4.099721000000	-0.350388000000
N	2.426849000000	5.402156000000	0.076893000000
C	3.622948000000	6.018112000000	0.375368000000
N	4.787437000000	5.442189000000	0.294177000000
C	4.798613000000	4.099009000000	-0.111358000000
N	3.618006000000	3.437514000000	-0.427286000000
H	1.512166000000	5.923426000000	0.076555000000
O	-5.922012000000	3.301047000000	0.110970000000
O	5.883233000000	3.468088000000	-0.166706000000
H	1.435052000000	2.676100000000	-1.299127000000
N	1.314711000000	3.474463000000	-0.682664000000
H	0.389626000000	3.958156000000	-0.640379000000
H	3.536558000000	7.048179000000	0.714575000000
H	-2.874805000000	-0.218571000000	0.459966000000
C	-2.411389000000	4.086598000000	-0.251955000000
N	-3.545959000000	4.755450000000	0.141628000000
C	-3.434824000000	6.096666000000	0.420293000000
N	-2.319050000000	6.765301000000	0.377017000000
C	-1.169541000000	6.046196000000	0.022249000000
N	-1.234717000000	4.701463000000	-0.318413000000
H	-4.472199000000	4.251050000000	0.163819000000
N	-3.722931000000	-0.739582000000	0.273315000000
O	-0.053711000000	6.627283000000	0.021953000000
H	-1.663201000000	2.310553000000	-0.807338000000
N	-2.534105000000	2.788508000000	-0.607503000000
H	-3.364993000000	2.223644000000	-0.298534000000
H	-4.361291000000	6.586839000000	0.713656000000
H	-3.667164000000	-1.744496000000	-0.024618000000
C	-4.841771000000	-0.006258000000	0.110371000000
N	-6.030211000000	-0.660995000000	-0.100190000000
C	3.945351000000	-2.886996000000	-3.737915000000
N	5.188681000000	-2.878443000000	-3.150470000000

C	5.665624000000	-4.067305000000	-2.651088000000
N	4.985935000000	-5.177169000000	-2.618768000000
C	3.665656000000	-5.120206000000	-3.105601000000
N	3.197314000000	-3.984156000000	-3.762808000000
H	5.760314000000	-1.986141000000	-3.145062000000
C	-0.632222000000	-6.968484000000	-2.710322000000
O	2.898513000000	-6.094679000000	-2.923158000000
H	2.566358000000	-1.750354000000	-4.663696000000
N	3.515940000000	-1.736842000000	-4.311030000000
H	3.930929000000	-0.821504000000	-4.028563000000
H	6.675501000000	-4.030443000000	-2.245293000000
N	-1.932722000000	-6.913280000000	-2.711859000000
C	4.130278000000	1.943290000000	-3.386253000000
N	4.895880000000	3.069460000000	-3.223581000000
C	6.243184000000	2.899467000000	-3.023102000000
N	6.852196000000	1.748931000000	-3.015714000000
C	6.047654000000	0.609296000000	-3.205624000000
N	4.669315000000	0.731218000000	-3.401807000000
H	4.444410000000	4.032825000000	-3.185079000000
C	-2.508818000000	-5.718466000000	-3.180335000000
O	6.570406000000	-0.527260000000	-3.171474000000
H	2.288362000000	1.293364000000	-3.855110000000
N	2.782038000000	2.104125000000	-3.497382000000
H	2.398825000000	3.054783000000	-3.718841000000
H	6.805831000000	3.816993000000	-2.854326000000
N	-1.724139000000	-4.737433000000	-3.786042000000
C	0.408206000000	4.856014000000	-3.753141000000
N	-0.186662000000	5.960294000000	-3.194886000000
C	0.636799000000	6.959699000000	-2.732714000000
N	1.937302000000	6.904599000000	-2.732125000000
C	2.514196000000	5.708618000000	-3.196660000000
N	1.730541000000	4.726021000000	-3.801167000000
H	-1.245946000000	6.029503000000	-3.133573000000
H	1.251204000000	-6.039424000000	-3.110640000000
O	3.741588000000	5.512298000000	-3.034375000000
H	0.040781000000	3.222807000000	-4.874299000000
N	-0.392379000000	3.869606000000	-4.224987000000
H	-1.436414000000	3.952568000000	-4.186234000000
H	0.126452000000	7.837384000000	-2.339362000000
H	-0.122547000000	-7.845194000000	-2.313951000000
C	-3.939078000000	2.874990000000	-3.754602000000
N	-5.183595000000	2.868668000000	-3.169647000000
C	-5.661428000000	4.059361000000	-2.675530000000
N	-4.981663000000	5.169248000000	-2.645755000000
C	-3.660399000000	5.110389000000	-3.129653000000
N	-3.190887000000	3.971988000000	-3.781936000000
H	-5.755256000000	1.976404000000	-3.161971000000
O	-3.736453000000	-5.521645000000	-3.020514000000
O	-2.893490000000	6.085396000000	-2.949063000000
H	-2.558107000000	1.734850000000	-4.672945000000
N	-3.508664000000	1.722760000000	-4.322830000000
H	-3.924140000000	0.808523000000	-4.037518000000
H	-6.672151000000	4.024074000000	-2.271705000000
H	-0.032759000000	-3.236909000000	-4.860112000000
C	-4.124559000000	-1.953671000000	-3.384704000000
N	-4.890353000000	-3.079339000000	-3.219512000000
C	-6.238119000000	-2.908797000000	-3.022581000000
N	-6.847269000000	-1.758314000000	-3.020631000000
C	-6.042476000000	-0.619260000000	-3.213141000000

N	-4.663720000000	-0.741744000000	-3.405989000000
H	-4.438906000000	-4.042539000000	-3.176888000000
N	0.399442000000	-3.882077000000	-4.208540000000
O	-6.565420000000	0.517343000000	-3.184355000000
H	-2.281752000000	-1.305136000000	-3.851798000000
N	-2.776047000000	-2.114719000000	-3.492247000000
H	-2.392332000000	-3.066102000000	-3.709795000000
H	-6.801006000000	-3.825776000000	-2.851675000000
H	1.443438000000	-3.965300000000	-4.168535000000
C	-0.401893000000	-4.867375000000	-3.735635000000
N	0.192032000000	-5.970350000000	-3.173832000000
C	3.919831000000	-2.403141000000	3.351162000000
N	5.285185000000	-2.428355000000	3.196757000000
C	5.885608000000	-3.654273000000	3.050580000000
N	5.263832000000	-4.796820000000	3.105807000000
C	3.873069000000	-4.745734000000	3.318986000000
N	3.210004000000	-3.524214000000	3.426077000000
H	5.832312000000	-1.518830000000	3.122544000000
C	-0.263879000000	-6.965712000000	2.870634000000
O	3.219761000000	-5.812960000000	3.381908000000
H	2.326221000000	-1.209595000000	3.633230000000
N	3.310926000000	-1.195277000000	3.394946000000
H	3.867469000000	-0.323741000000	3.561366000000
H	6.959166000000	-3.624940000000	2.865479000000
N	-1.566264000000	-7.002638000000	2.877634000000
C	4.294242000000	2.344862000000	3.765985000000
N	4.895241000000	3.459457000000	3.224280000000
C	6.164570000000	3.312830000000	2.712047000000
N	6.810714000000	2.184206000000	2.654971000000
C	6.123845000000	1.041945000000	3.105886000000
N	4.878889000000	1.150720000000	3.717886000000
H	4.400614000000	4.393905000000	3.261521000000
C	-2.227700000000	-5.863731000000	3.375771000000
O	6.631567000000	-0.091037000000	2.923803000000
H	2.722071000000	1.684651000000	4.839506000000
N	3.085653000000	2.491440000000	4.346929000000
H	2.528317000000	3.375323000000	4.287982000000
H	6.613435000000	4.224050000000	2.318787000000
N	-1.511713000000	-4.804976000000	3.929446000000
C	0.177850000000	4.860125000000	3.879711000000
N	-0.491821000000	5.927757000000	3.324179000000
C	0.259576000000	6.974881000000	2.847633000000
N	1.561935000000	7.012029000000	2.856902000000
C	2.222659000000	5.874404000000	3.358923000000
N	1.505809000000	4.816800000000	3.913667000000
H	-1.552471000000	5.933973000000	3.314019000000
H	1.547471000000	-5.923921000000	3.337794000000
O	3.471453000000	5.801709000000	3.274842000000
H	-0.043718000000	3.064937000000	4.756526000000
N	-0.565452000000	3.862471000000	4.411888000000
H	-1.553302000000	3.721496000000	4.098941000000
H	-0.311269000000	7.799721000000	2.423632000000
H	0.307601000000	-7.791609000000	2.449558000000
C	-3.924704000000	2.413422000000	3.333581000000
N	-5.289955000000	2.438154000000	3.178316000000
C	-5.890277000000	3.663627000000	3.027952000000
N	-5.268572000000	4.806349000000	3.080274000000
C	-3.878015000000	4.755931000000	3.294946000000
N	-3.214967000000	3.534715000000	3.405715000000

H	-5.837062000000	1.528400000000	3.106577000000
O	-3.476322000000	-5.791005000000	3.289134000000
O	-3.224834000000	5.823339000000	3.355835000000
H	-2.331287000000	1.220776000000	3.620320000000
N	-3.315754000000	1.205680000000	3.381061000000
H	-3.872467000000	0.334797000000	3.550384000000
H	-6.963679000000	3.633713000000	2.842022000000
H	0.036680000000	-3.051734000000	4.771773000000
C	-4.299811000000	-2.332637000000	3.765646000000
N	-4.900607000000	-3.449340000000	3.228124000000
C	-6.169542000000	-3.304609000000	2.714337000000
N	-6.815509000000	-2.176156000000	2.652273000000
C	-6.128835000000	-1.032176000000	3.099056000000
N	-4.884298000000	-1.138633000000	3.712296000000
H	-4.406029000000	-4.383616000000	3.269479000000
N	0.558854000000	-3.849934000000	4.429351000000
O	-6.636316000000	0.100135000000	2.912136000000
H	-2.728652000000	-1.668255000000	4.837989000000
N	-3.091469000000	-2.476796000000	4.347725000000
H	-2.534721000000	-3.361404000000	4.294435000000
H	-6.618207000000	-4.217353000000	2.324390000000
H	1.547207000000	-3.709661000000	4.117727000000
C	-0.183690000000	-4.848610000000	3.898042000000
N	0.486806000000	-5.917586000000	3.346085000000

(C_{2h})	*b*AM'$_6$-Br$^-$-[*b*AM'$_6$]-Br$^-$-*b*AM'$_6$		
C	-2.597229000000	-3.895121000000	0.000000000000
N	-2.642837000000	-5.275680000000	0.000000000000
C	-3.878343000000	-5.869496000000	0.000000000000
N	-5.018008000000	-5.235379000000	0.000000000000
C	-4.946042000000	-3.829614000000	0.000000000000
N	-3.723316000000	-3.186112000000	0.000000000000
H	-1.754348000000	-5.848047000000	0.000000000000
C	-7.024777000000	0.424833000000	0.000000000000
O	-6.001055000000	-3.144094000000	0.000000000000
H	-1.358642000000	-2.279586000000	0.000000000000
N	-1.404634000000	-3.293788000000	0.000000000000
H	-0.518337000000	-3.860329000000	0.000000000000
H	-3.861115000000	-6.958398000000	0.000000000000
N	-7.043958000000	1.728888000000	0.000000000000
C	2.075327000000	-4.195422000000	0.000000000000
N	3.247632000000	-4.926083000000	0.000000000000
C	3.142754000000	-6.292869000000	0.000000000000
N	2.023070000000	-6.961432000000	0.000000000000
C	0.842263000000	-6.195528000000	0.000000000000
N	0.897590000000	-4.814753000000	0.000000000000
H	4.187725000000	-4.443387000000	0.000000000000
C	-5.790255000000	2.368427000000	0.000000000000
O	-0.279141000000	-6.766190000000	0.000000000000
H	1.298350000000	-2.313889000000	0.000000000000
N	2.152878000000	-2.862119000000	0.000000000000
H	3.087400000000	-2.379320000000	0.000000000000
H	4.093684000000	-6.823583000000	0.000000000000
N	-4.622162000000	1.629912000000	0.000000000000
C	4.674861000000	-0.300278000000	0.000000000000
N	5.893718000000	0.349609000000	0.000000000000
C	7.024777000000	-0.424833000000	0.000000000000
N	7.043958000000	-1.728888000000	0.000000000000

C	5.790255000000	-2.368427000000	0.000000000000
N	4.622162000000	-1.629912000000	0.000000000000
H	5.945390000000	1.405230000000	0.000000000000
H	-5.945390000000	-1.405230000000	0.000000000000
O	5.722765000000	-3.624759000000	0.000000000000
H	2.657122000000	-0.034098000000	0.000000000000
N	3.558531000000	0.432952000000	0.000000000000
H	3.606782000000	1.483958000000	0.000000000000
H	7.959937000000	0.133274000000	0.000000000000
H	-7.959937000000	-0.133274000000	0.000000000000
C	2.597229000000	3.895121000000	0.000000000000
N	2.642837000000	5.275680000000	0.000000000000
C	3.878343000000	5.869496000000	0.000000000000
N	5.018008000000	5.235379000000	0.000000000000
C	4.946042000000	3.829614000000	0.000000000000
N	3.723316000000	3.186112000000	0.000000000000
H	1.754348000000	5.848047000000	0.000000000000
O	-5.722765000000	3.624759000000	0.000000000000
O	6.001055000000	3.144094000000	0.000000000000
H	1.358642000000	2.279586000000	0.000000000000
N	1.404634000000	3.293788000000	0.000000000000
H	0.518337000000	3.860329000000	0.000000000000
H	3.861115000000	6.958398000000	0.000000000000
H	-2.657122000000	0.034098000000	0.000000000000
C	-2.075327000000	4.195422000000	0.000000000000
N	-3.247632000000	4.926083000000	0.000000000000
C	-3.142754000000	6.292869000000	0.000000000000
N	-2.023070000000	6.961432000000	0.000000000000
C	-0.842263000000	6.195528000000	0.000000000000
N	-0.897590000000	4.814753000000	0.000000000000
H	-4.187725000000	4.443387000000	0.000000000000
N	-3.558531000000	-0.432952000000	0.000000000000
O	0.279141000000	6.766190000000	0.000000000000
H	-1.298350000000	2.313889000000	0.000000000000
N	-2.152878000000	2.862119000000	0.000000000000
H	-3.087400000000	2.379320000000	0.000000000000
H	-4.093684000000	6.823583000000	0.000000000000
H	-3.606782000000	-1.483958000000	0.000000000000
C	-4.674861000000	0.300278000000	0.000000000000
N	-5.893718000000	-0.349609000000	0.000000000000
C	3.921604000000	-2.591191000000	-3.058532000000
N	5.305487000000	-2.630458000000	-3.105039000000
C	5.901446000000	-3.866340000000	-3.081736000000
N	5.273914000000	-5.008520000000	-3.091573000000
C	3.861086000000	-4.943009000000	-3.088656000000
N	3.217056000000	-3.720579000000	-3.040465000000
H	5.872560000000	-1.738780000000	-3.089810000000
C	-0.399999000000	-7.046880000000	-3.080551000000
O	3.182498000000	-6.000286000000	-3.104118000000
H	2.315079000000	-1.347253000000	-2.887284000000
N	3.320758000000	-1.398773000000	-3.038522000000
H	3.887236000000	-0.514926000000	-3.008969000000
H	6.991303000000	-3.846493000000	-3.046863000000
N	-1.702926000000	-7.073335000000	-3.090276000000
C	4.207098000000	2.101291000000	-3.052715000000
N	4.933233000000	3.279877000000	-3.103170000000
C	6.301522000000	3.177231000000	-3.082557000000
N	6.975970000000	2.062246000000	-3.092166000000
C	6.212575000000	0.871803000000	-3.086574000000

N	4.832036000000	0.926127000000	-3.034216000000
H	4.444472000000	4.216686000000	-3.089073000000
C	-2.351833000000	-5.816853000000	-3.088786000000
O	6.788593000000	-0.244707000000	-3.103194000000
H	2.326111000000	1.333717000000	-2.875914000000
N	2.874290000000	2.178416000000	-3.027513000000
H	2.392161000000	3.111077000000	-3.005510000000
H	6.830089000000	4.130592000000	-3.050229000000
N	-1.614155000000	-4.648227000000	-3.043307000000
C	0.283929000000	4.695052000000	-3.061912000000
N	-0.373395000000	5.913559000000	-3.105455000000
C	0.399999000000	7.046880000000	-3.080551000000
N	1.702926000000	7.073335000000	-3.090276000000
C	2.351833000000	5.816853000000	-3.088786000000
N	1.614155000000	4.648227000000	-3.043307000000
H	-1.429175000000	5.958516000000	-3.090487000000
H	1.429175000000	-5.958516000000	-3.090487000000
O	3.606671000000	5.756722000000	-3.103554000000
H	0.009041000000	2.681982000000	-2.894924000000
N	-0.449138000000	3.578951000000	-3.044577000000
H	-1.498019000000	3.627479000000	-3.015220000000
H	-0.161099000000	7.981382000000	-3.044503000000
H	0.161099000000	-7.981382000000	-3.044503000000
C	-3.921604000000	2.591191000000	-3.058532000000
N	-5.305487000000	2.630458000000	-3.105039000000
C	-5.901446000000	3.866340000000	-3.081736000000
N	-5.273914000000	5.008520000000	-3.091573000000
C	-3.861086000000	4.943009000000	-3.088656000000
N	-3.217056000000	3.720579000000	-3.040465000000
H	-5.872560000000	1.738780000000	-3.089810000000
O	-3.606671000000	-5.756722000000	-3.103554000000
O	-3.182498000000	6.000286000000	-3.104118000000
H	-2.315079000000	1.347253000000	-2.887284000000
N	-3.320758000000	1.398773000000	-3.038522000000
H	-3.887236000000	0.514926000000	-3.008969000000
H	-6.991303000000	3.846493000000	-3.046863000000
H	-0.009041000000	-2.681982000000	-2.894924000000
C	-4.207098000000	-2.101291000000	-3.052715000000
N	-4.933233000000	-3.279877000000	-3.103170000000
C	-6.301522000000	-3.177231000000	-3.082557000000
N	-6.975970000000	-2.062246000000	-3.092166000000
C	-6.212575000000	-0.871803000000	-3.086574000000
N	-4.832036000000	-0.926127000000	-3.034216000000
H	-4.444472000000	-4.216686000000	-3.089073000000
N	0.449138000000	-3.578951000000	-3.044577000000
O	-6.788593000000	0.244707000000	-3.103194000000
H	-2.326111000000	-1.333717000000	-2.875914000000
N	-2.874290000000	-2.178416000000	-3.027513000000
H	-2.392161000000	-3.111077000000	-3.005510000000
H	-6.830089000000	-4.130592000000	-3.050229000000
H	1.498019000000	-3.627479000000	-3.015220000000
C	-0.283929000000	-4.695052000000	-3.061912000000
N	0.373395000000	-5.913559000000	-3.105455000000
Br	0.000000000000	0.000000000000	-2.032130000000
C	3.921604000000	-2.591191000000	3.058532000000
N	5.305487000000	-2.630458000000	3.105039000000
C	5.901446000000	-3.866340000000	3.081736000000
N	5.273914000000	-5.008520000000	3.091573000000
C	3.861086000000	-4.943009000000	3.088656000000

N	3.217056000000	-3.720579000000	3.040465000000
H	5.872560000000	-1.738780000000	3.089810000000
C	-0.399999000000	-7.046880000000	3.080551000000
O	3.182498000000	-6.000286000000	3.104118000000
H	2.315079000000	-1.347253000000	2.887284000000
N	3.320758000000	-1.398773000000	3.038522000000
H	3.887236000000	-0.514926000000	3.008969000000
H	6.991303000000	-3.846493000000	3.046863000000
N	-1.702926000000	-7.073335000000	3.090276000000
C	4.207098000000	2.101291000000	3.052715000000
N	4.933233000000	3.279877000000	3.103170000000
C	6.301522000000	3.177231000000	3.082557000000
N	6.975970000000	2.062246000000	3.092166000000
C	6.212575000000	0.871803000000	3.086574000000
N	4.832036000000	0.926127000000	3.034216000000
H	4.444472000000	4.216686000000	3.089073000000
C	-2.351833000000	-5.816853000000	3.088786000000
O	6.788593000000	-0.244707000000	3.103194000000
H	2.326111000000	1.333717000000	2.875914000000
N	2.874290000000	2.178416000000	3.027513000000
H	2.392161000000	3.111077000000	3.005510000000
H	6.830089000000	4.130592000000	3.050229000000
N	-1.614155000000	-4.648227000000	3.043307000000
C	0.283929000000	4.695052000000	3.061912000000
N	-0.373395000000	5.913559000000	3.105455000000
C	0.399999000000	7.046880000000	3.080551000000
N	1.702926000000	7.073335000000	3.090276000000
C	2.351833000000	5.816853000000	3.088786000000
N	1.614155000000	4.648227000000	3.043307000000
H	-1.429175000000	5.958516000000	3.090487000000
H	1.429175000000	-5.958516000000	3.090487000000
O	3.606671000000	5.756722000000	3.103554000000
H	0.009041000000	2.681982000000	2.894924000000
N	-0.449138000000	3.578951000000	3.044577000000
H	-1.498019000000	3.627479000000	3.015220000000
H	-0.161099000000	7.981382000000	3.044503000000
H	0.161099000000	-7.981382000000	3.044503000000
C	-3.921604000000	2.591191000000	3.058532000000
N	-5.305487000000	2.630458000000	3.105039000000
C	-5.901446000000	3.866340000000	3.081736000000
N	-5.273914000000	5.008520000000	3.091573000000
C	-3.861086000000	4.943009000000	3.088656000000
N	-3.217056000000	3.720579000000	3.040465000000
H	-5.872560000000	1.738780000000	3.089810000000
O	-3.606671000000	-5.756722000000	3.103554000000
O	-3.182498000000	6.000286000000	3.104118000000
H	-2.315079000000	1.347253000000	2.887284000000
N	-3.320758000000	1.398773000000	3.038522000000
H	-3.887236000000	0.514926000000	3.008969000000
H	-6.991303000000	3.846493000000	3.046863000000
H	-0.009041000000	-2.681982000000	2.894924000000
C	-4.207098000000	-2.101291000000	3.052715000000
N	-4.933233000000	-3.279877000000	3.103170000000
C	-6.301522000000	-3.177231000000	3.082557000000
N	-6.975970000000	-2.062246000000	3.092166000000
C	-6.212575000000	-0.871803000000	3.086574000000
N	-4.832036000000	-0.926127000000	3.034216000000
H	-4.444472000000	-4.216686000000	3.089073000000
N	0.449138000000	-3.578951000000	3.044577000000

O	-6.788593000000	0.244707000000	3.103194000000
H	-2.326111000000	-1.333717000000	2.875914000000
N	-2.874290000000	-2.178416000000	3.027513000000
H	-2.392161000000	-3.111077000000	3.005510000000
H	-6.830089000000	-4.130592000000	3.050229000000
H	1.498019000000	-3.627479000000	3.015220000000
C	-0.283929000000	-4.695052000000	3.061912000000
N	0.373395000000	-5.913559000000	3.105455000000
Br	0.000000000000	0.000000000000	2.032130000000

(C_1)	bAM'$_6$-Br$^-$-[bAM'$_6$]-Br$^-$-bAM'$_6$		
C	-2.597250000000	-3.895131000000	0.000001000000
N	-2.642864000000	-5.275691000000	0.000000000000
C	-3.878374000000	-5.869497000000	-0.000001000000
N	-5.018036000000	-5.235375000000	-0.000002000000
C	-4.946062000000	-3.829609000000	-0.000001000000
N	-3.723331000000	-3.186113000000	0.000001000000
H	-1.754385000000	-5.848073000000	0.000000000000
C	-7.024781000000	0.424836000000	0.000001000000
O	-6.001071000000	-3.144082000000	-0.000001000000
H	-1.358637000000	-2.279607000000	0.000003000000
N	-1.404651000000	-3.293808000000	0.000002000000
H	-0.518365000000	-3.860360000000	0.000002000000
H	-3.861149000000	-6.958400000000	-0.000001000000
N	-7.043976000000	1.728892000000	0.000001000000
C	2.075324000000	-4.195386000000	0.000001000000
N	3.247633000000	-4.926040000000	0.000000000000
C	3.142768000000	-6.292829000000	-0.000001000000
N	2.023088000000	-6.961400000000	-0.000001000000
C	0.842275000000	-6.195504000000	-0.000000000000
N	0.897594000000	-4.814729000000	0.000001000000
H	4.187721000000	-4.443339000000	0.000001000000
C	-5.790277000000	2.368441000000	0.000000000000
O	-0.279127000000	-6.766169000000	-0.000000000000
H	1.298322000000	-2.313859000000	0.000004000000
N	2.152855000000	-2.862083000000	0.000003000000
H	3.087365000000	-2.379262000000	0.000002000000
H	4.093698000000	-6.823542000000	-0.000001000000
N	-4.622177000000	1.629937000000	-0.000001000000
C	4.674859000000	-0.300301000000	-0.000000000000
N	5.893711000000	0.349594000000	0.000001000000
C	7.024778000000	-0.424836000000	0.000000000000
N	7.043973000000	-1.728891000000	0.000000000000
C	5.790273000000	-2.368440000000	-0.000000000000
N	4.622174000000	-1.629935000000	-0.000000000000
H	5.945376000000	1.405213000000	0.000001000000
H	-5.945378000000	-1.405212000000	-0.000001000000
O	5.722801000000	-3.624774000000	-0.000000000000
H	2.657119000000	-0.034113000000	-0.000002000000
N	3.558531000000	0.432932000000	-0.000001000000
H	3.606792000000	1.483938000000	-0.000002000000
H	7.959931000000	0.133283000000	0.000001000000
H	-7.959934000000	-0.133283000000	0.000001000000
C	2.597254000000	3.895133000000	0.000001000000
N	2.642868000000	5.275693000000	0.000000000000
C	3.878378000000	5.869499000000	0.000000000000
N	5.018039000000	5.235376000000	0.000001000000
C	4.946065000000	3.829610000000	0.000002000000

N	3.723334000000	3.186114000000	0.000002000000
H	1.754388000000	5.848075000000	-0.000001000000
O	-5.722806000000	3.624775000000	0.000000000000
O	6.001073000000	3.144082000000	0.000002000000
H	1.358639000000	2.279611000000	-0.000001000000
N	1.404654000000	3.293812000000	-0.000000000000
H	0.518368000000	3.860365000000	-0.000001000000
H	3.861153000000	6.958401000000	-0.000000000000
H	-2.657122000000	0.034115000000	-0.000003000000
C	-2.075324000000	4.195382000000	-0.000003000000
N	-3.247634000000	4.926035000000	-0.000001000000
C	-3.142769000000	6.292824000000	0.000001000000
N	-2.023090000000	6.961396000000	0.000001000000
C	-0.842276000000	6.195500000000	-0.000001000000
N	-0.897595000000	4.814726000000	-0.000004000000
H	-4.187721000000	4.443334000000	-0.000001000000
N	-3.558534000000	-0.432930000000	-0.000002000000
O	0.279126000000	6.766166000000	-0.000000000000
H	-1.298320000000	2.313856000000	-0.000007000000
N	-2.152854000000	2.862079000000	-0.000005000000
H	-3.087364000000	2.379256000000	-0.000003000000
H	-4.093699000000	6.823537000000	0.000002000000
H	-3.606795000000	-1.483937000000	-0.000001000000
C	-4.674863000000	0.300303000000	-0.000001000000
N	-5.893714000000	-0.349593000000	-0.000000000000
C	3.921615000000	-2.591193000000	-3.058558000000
N	5.305499000000	-2.630467000000	-3.105079000000
C	5.901449000000	-3.866351000000	-3.081743000000
N	5.273912000000	-5.008528000000	-3.091552000000
C	3.861083000000	-4.943011000000	-3.088645000000
N	3.217060000000	-3.720575000000	-3.040467000000
H	5.872581000000	-1.738794000000	-3.089876000000
C	-0.400010000000	-7.046890000000	-3.080553000000
O	3.182488000000	-6.000281000000	-3.104091000000
H	2.315102000000	-1.347239000000	-2.887292000000
N	3.320775000000	-1.398772000000	-3.038551000000
H	3.887258000000	-0.514931000000	-3.008973000000
H	6.991306000000	-3.846507000000	-3.046861000000
N	-1.702938000000	-7.073348000000	-3.090281000000
C	4.207074000000	2.101288000000	-3.052708000000
N	4.933199000000	3.279877000000	-3.103177000000
C	6.301491000000	3.177247000000	-3.082535000000
N	6.975948000000	2.062267000000	-3.092113000000
C	6.212564000000	0.871818000000	-3.086531000000
N	4.832027000000	0.926134000000	-3.034182000000
H	4.444433000000	4.216682000000	-3.089085000000
C	-2.351847000000	-5.816866000000	-3.088814000000
O	6.788585000000	-0.244689000000	-3.103142000000
H	2.326106000000	1.333693000000	-2.875893000000
N	2.874266000000	2.178392000000	-3.027515000000
H	2.392120000000	3.111044000000	-3.005543000000
H	6.830053000000	4.130610000000	-3.050202000000
N	-1.614171000000	-4.648239000000	-3.043321000000
C	0.283947000000	4.695056000000	-3.061917000000
N	-0.373380000000	5.913561000000	-3.105465000000
C	0.400009000000	7.046886000000	-3.080556000000
N	1.702937000000	7.073345000000	-3.090287000000
C	2.351846000000	5.816863000000	-3.088816000000
N	1.614172000000	4.648235000000	-3.043318000000

H	-1.429157000000	5.958520000000	-3.090497000000
H	1.429157000000	-5.958525000000	-3.090501000000
O	3.606683000000	5.756740000000	-3.103610000000
H	0.009050000000	2.681989000000	-2.894928000000
N	-0.449126000000	3.578960000000	-3.044558000000
H	-1.498007000000	3.627503000000	-3.015194000000
H	-0.161095000000	7.981383000000	-3.044496000000
H	0.161093000000	-7.981388000000	-3.044490000000
C	-3.921614000000	2.591197000000	-3.058560000000
N	-5.305498000000	2.630469000000	-3.105077000000
C	-5.901449000000	3.866354000000	-3.081739000000
N	-5.273912000000	5.008530000000	-3.091548000000
C	-3.861084000000	4.943014000000	-3.088646000000
N	-3.217059000000	3.720579000000	-3.040470000000
H	-5.872580000000	1.738797000000	-3.089874000000
O	-3.606684000000	-5.756742000000	-3.103607000000
O	-3.182489000000	6.000284000000	-3.104093000000
H	-2.315102000000	1.347243000000	-2.887293000000
N	-3.320775000000	1.398776000000	-3.038552000000
H	-3.887257000000	0.514935000000	-3.008973000000
H	-6.991306000000	3.846509000000	-3.046854000000
H	-0.009049000000	-2.681993000000	-2.894938000000
C	-4.207072000000	-2.101289000000	-3.052713000000
N	-4.933199000000	-3.279878000000	-3.103171000000
C	-6.301490000000	-3.177245000000	-3.082527000000
N	-6.975946000000	-2.062264000000	-3.092109000000
C	-6.212560000000	-0.871816000000	-3.086535000000
N	-4.832022000000	-0.926134000000	-3.034192000000
H	-4.444434000000	-4.216683000000	-3.089076000000
N	0.449127000000	-3.578965000000	-3.044566000000
O	-6.788580000000	0.244691000000	-3.103149000000
H	-2.326097000000	-1.333698000000	-2.875934000000
N	-2.874264000000	-2.178397000000	-3.027531000000
H	-2.392119000000	-3.111049000000	-3.005557000000
H	-6.830054000000	-4.130607000000	-3.050189000000
H	1.498008000000	-3.627509000000	-3.015202000000
C	-0.283947000000	-4.695061000000	-3.061922000000
N	0.373379000000	-5.913566000000	-3.105468000000
Br	-0.000014000000	0.000020000000	-2.03205700000
C	3.921614000000	-2.591192000000	3.058559000000
N	5.305498000000	-2.630466000000	3.105076000000
C	5.901448000000	-3.866351000000	3.081737000000
N	5.273911000000	-5.008527000000	3.091546000000
C	3.861082000000	-4.943010000000	3.088645000000
N	3.217059000000	-3.720574000000	3.040470000000
H	5.872580000000	-1.738793000000	3.089874000000
C	-0.400012000000	-7.046890000000	3.080556000000
O	3.182487000000	-6.000280000000	3.104093000000
H	2.315101000000	-1.347238000000	2.887295000000
N	3.320775000000	-1.398772000000	3.038553000000
H	3.887257000000	-0.514931000000	3.008974000000
H	6.991305000000	-3.846507000000	3.046851000000
N	-1.702939000000	-7.073348000000	3.090284000000
C	4.207076000000	2.101289000000	3.052711000000
N	4.933201000000	3.279878000000	3.103177000000
C	6.301493000000	3.177247000000	3.082533000000
N	6.975949000000	2.062266000000	3.092112000000
C	6.212564000000	0.871818000000	3.086535000000
N	4.832027000000	0.926135000000	3.034188000000

H	4.444435000000	4.216683000000	3.089084000000
C	-2.351848000000	-5.816866000000	3.088814000000
O	6.788585000000	-0.244689000000	3.103149000000
H	2.326107000000	1.333695000000	2.875898000000
N	2.874267000000	2.178394000000	3.027518000000
H	2.392122000000	3.111046000000	3.005544000000
H	6.830056000000	4.130609000000	3.050197000000
N	-1.614173000000	-4.648239000000	3.043317000000
C	0.283946000000	4.695056000000	3.061919000000
N	-0.373381000000	5.913562000000	3.105465000000
C	0.400009000000	7.046886000000	3.080554000000
N	1.702936000000	7.073344000000	3.090286000000
C	2.351846000000	5.816862000000	3.088818000000
N	1.614170000000	4.648235000000	3.043321000000
H	-1.429158000000	5.958520000000	3.090495000000
H	1.429155000000	-5.958525000000	3.090500000000
O	3.606683000000	5.756739000000	3.103612000000
H	0.009048000000	2.681988000000	2.894932000000
N	-0.449128000000	3.578961000000	3.044560000000
H	-1.498009000000	3.627503000000	3.015195000000
H	-0.161094000000	7.981384000000	3.044492000000
H	0.161092000000	-7.981388000000	3.044495000000
C	-3.921616000000	2.591198000000	3.058560000000
N	-5.305500000000	2.630471000000	3.105080000000
C	-5.901450000000	3.866356000000	3.081742000000
N	-5.273913000000	5.008532000000	3.091551000000
C	-3.861085000000	4.943015000000	3.088645000000
N	-3.217061000000	3.720580000000	3.040468000000
H	-5.872582000000	1.738798000000	3.089877000000
O	-3.606685000000	-5.756742000000	3.103608000000
O	-3.182490000000	6.000285000000	3.104091000000
H	-2.315103000000	1.347244000000	2.887295000000
N	-3.320777000000	1.398777000000	3.038554000000
H	-3.887258000000	0.514936000000	3.008977000000
H	-6.991307000000	3.846511000000	3.046858000000
H	-0.009051000000	-2.681994000000	2.894930000000
C	-4.207070000000	-2.101288000000	3.052712000000
N	-4.933197000000	-3.279877000000	3.103170000000
C	-6.301488000000	-3.177245000000	3.082528000000
N	-6.975944000000	-2.062265000000	3.092110000000
C	-6.212559000000	-0.871816000000	3.086534000000
N	-4.832022000000	-0.926133000000	3.034190000000
H	-4.444431000000	-4.216682000000	3.089074000000
N	0.449125000000	-3.578966000000	3.044560000000
O	-6.788580000000	0.244691000000	3.103146000000
H	-2.326095000000	-1.333695000000	2.875933000000
N	-2.874262000000	-2.178394000000	3.027530000000
H	-2.392117000000	-3.111047000000	3.005557000000
H	-6.830051000000	-4.130608000000	3.050191000000
H	1.498007000000	-3.627509000000	3.015197000000
C	-0.283948000000	-4.695061000000	3.061919000000
N	0.373378000000	-5.913566000000	3.105467000000
Br	-0.000014000000	-0.000009000000	2.032067000000

(C_{2h})	*b*AM'$_6$-I$^-$-[*b*AM'$_6$]-I$^-$-*b*AM'$_6$		
C	-2.570445000000	-3.942316000000	0.000000000000
N	-2.612387000000	-5.323143000000	0.000000000000
C	-3.846297000000	-5.921008000000	0.000000000000

N	-4.986954000000	-5.289290000000	0.000000000000
C	-4.918812000000	-3.883148000000	0.000000000000
N	-3.698759000000	-3.234953000000	0.000000000000
H	-1.721208000000	-5.888952000000	0.000000000000
C	-7.052978000000	0.373369000000	0.000000000000
O	-5.977403000000	-3.202608000000	0.000000000000
H	-1.342083000000	-2.324208000000	0.000000000000
N	-1.380051000000	-3.338541000000	0.000000000000
H	-0.487663000000	-3.894729000000	0.000000000000
H	-3.825858000000	-7.009728000000	0.000000000000
N	-7.075139000000	1.677080000000	0.000000000000
C	2.128967000000	-4.200695000000	0.000000000000
N	3.303606000000	-4.927665000000	0.000000000000
C	3.203483000000	-6.295241000000	0.000000000000
N	2.085632000000	-6.966317000000	0.000000000000
C	0.902211000000	-6.204017000000	0.000000000000
N	0.951963000000	-4.823276000000	0.000000000000
H	4.238926000000	-4.438499000000	0.000000000000
C	-5.823230000000	2.320478000000	0.000000000000
O	-0.216769000000	-6.780149000000	0.000000000000
H	1.343464000000	-2.327178000000	0.000000000000
N	2.202475000000	-2.867903000000	0.000000000000
H	3.130371000000	-2.373284000000	0.000000000000
H	4.156097000000	-6.822680000000	0.000000000000
N	-4.652292000000	1.586941000000	0.000000000000
C	4.701860000000	-0.256170000000	0.000000000000
N	5.918875000000	0.397336000000	0.000000000000
C	7.052978000000	-0.373369000000	0.000000000000
N	7.075139000000	-1.677080000000	0.000000000000
C	5.823230000000	-2.320478000000	0.000000000000
N	4.652292000000	-1.586941000000	0.000000000000
H	5.963046000000	1.451938000000	0.000000000000
H	-5.963046000000	-1.451938000000	0.000000000000
O	5.761970000000	-3.577488000000	0.000000000000
H	2.686726000000	-0.000596000000	0.000000000000
N	3.584176000000	0.473592000000	0.000000000000
H	3.619358000000	1.524664000000	0.000000000000
H	7.986123000000	0.187821000000	0.000000000000
H	-7.986123000000	-0.187821000000	0.000000000000
C	2.570445000000	3.942316000000	0.000000000000
N	2.612387000000	5.323143000000	0.000000000000
C	3.846297000000	5.921008000000	0.000000000000
N	4.986954000000	5.289290000000	0.000000000000
C	4.918812000000	3.883148000000	0.000000000000
N	3.698759000000	3.234953000000	0.000000000000
H	1.721208000000	5.888952000000	0.000000000000
O	-5.761970000000	3.577488000000	0.000000000000
O	5.977403000000	3.202608000000	0.000000000000
H	1.342083000000	2.324208000000	0.000000000000
N	1.380051000000	3.338541000000	0.000000000000
H	0.487663000000	3.894729000000	0.000000000000
H	3.825858000000	7.009728000000	0.000000000000
H	-2.686726000000	0.000596000000	0.000000000000
C	-2.128967000000	4.200695000000	0.000000000000
N	-3.303606000000	4.927665000000	0.000000000000
C	-3.203483000000	6.295241000000	0.000000000000
N	-2.085632000000	6.966317000000	0.000000000000
C	-0.902211000000	6.204017000000	0.000000000000
N	-0.951963000000	4.823276000000	0.000000000000

H	-4.238926000000	4.438499000000	0.000000000000
N	-3.584176000000	-0.473592000000	0.000000000000
O	0.216769000000	6.780149000000	0.000000000000
H	-1.343464000000	2.327178000000	0.000000000000
N	-2.202475000000	2.867903000000	0.000000000000
H	-3.130371000000	2.373284000000	0.000000000000
H	-4.156097000000	6.822680000000	0.000000000000
H	-3.619358000000	-1.524664000000	0.000000000000
C	-4.701860000000	0.256170000000	0.000000000000
N	-5.918875000000	-0.397336000000	0.000000000000
C	3.974945000000	-2.619240000000	-3.117313000000
N	5.358408000000	-2.677682000000	-3.102356000000
C	5.935449000000	-3.920270000000	-3.016976000000
N	5.291080000000	-5.052846000000	-3.022828000000
C	3.879017000000	-4.969207000000	-3.081105000000
N	3.251110000000	-3.737166000000	-3.096849000000
H	5.926508000000	-1.789882000000	-3.083409000000
C	-0.428900000000	-7.102163000000	-3.020999000000
O	3.188421000000	-6.018232000000	-3.090229000000
H	2.381480000000	-1.363141000000	-3.048124000000
N	3.391624000000	-1.419010000000	-3.161231000000
H	3.951593000000	-0.532393000000	-3.113590000000
H	7.022625000000	-3.914510000000	-2.932320000000
N	-1.731930000000	-7.109353000000	-3.025844000000
C	4.256737000000	2.132575000000	-3.113173000000
N	4.999014000000	3.301502000000	-3.099854000000
C	6.363532000000	3.179908000000	-3.013056000000
N	7.021824000000	2.055409000000	-3.017347000000
C	6.243594000000	0.874238000000	-3.075309000000
N	4.862610000000	0.946732000000	-3.091328000000
H	4.513849000000	4.237236000000	-3.082967000000
C	-2.364954000000	-5.844320000000	-3.079596000000
O	6.807203000000	-0.248210000000	-3.084378000000
H	2.371435000000	1.382079000000	-3.043052000000
N	2.925703000000	2.228291000000	-3.157496000000
H	2.438468000000	3.156802000000	-3.111158000000
H	6.902175000000	4.124228000000	-2.929017000000
N	-1.611051000000	-4.684891000000	-3.090734000000
C	0.281148000000	4.753765000000	-3.112172000000
N	-0.359526000000	5.981374000000	-3.102392000000
C	0.428900000000	7.102163000000	-3.020999000000
N	1.731930000000	7.109353000000	-3.025844000000
C	2.364954000000	5.844320000000	-3.079596000000
N	1.611051000000	4.684891000000	-3.090734000000
H	-1.412493000000	6.029320000000	-3.085330000000
H	1.412493000000	-6.029320000000	-3.085330000000
O	3.618703000000	5.769976000000	-3.089401000000
H	-0.011612000000	2.746160000000	-3.035619000000
N	-0.467504000000	3.649002000000	-3.151292000000
H	-1.515629000000	3.691242000000	-3.109595000000
H	-0.118836000000	8.041638000000	-2.940630000000
H	0.118836000000	-8.041638000000	-2.940630000000
C	-3.974945000000	2.619240000000	-3.117313000000
N	-5.358408000000	2.677682000000	-3.102356000000
C	-5.935449000000	3.920270000000	-3.016976000000
N	-5.291080000000	5.052846000000	-3.022828000000
C	-3.879017000000	4.969207000000	-3.081105000000
N	-3.251110000000	3.737166000000	-3.096849000000
H	-5.926508000000	1.789882000000	-3.083409000000

O	-3.618703000000	-5.769976000000	-3.089401000000
O	-3.188421000000	6.018232000000	-3.090229000000
H	-2.381480000000	1.363141000000	-3.048124000000
N	-3.391624000000	1.419010000000	-3.161231000000
H	-3.951593000000	0.532393000000	-3.113590000000
H	-7.022625000000	3.914510000000	-2.932320000000
H	0.011612000000	-2.746160000000	-3.035619000000
C	-4.256737000000	-2.132575000000	-3.113173000000
N	-4.999014000000	-3.301502000000	-3.099854000000
C	-6.363532000000	-3.179908000000	-3.013056000000
N	-7.021824000000	-2.055409000000	-3.017347000000
C	-6.243594000000	-0.874238000000	-3.075309000000
N	-4.862610000000	-0.946732000000	-3.091328000000
H	-4.513849000000	-4.237236000000	-3.082967000000
N	0.467504000000	-3.649002000000	-3.151292000000
O	-6.807203000000	0.248210000000	-3.084378000000
H	-2.371435000000	-1.382079000000	-3.043052000000
N	-2.925703000000	-2.228291000000	-3.157496000000
H	-2.438468000000	-3.156802000000	-3.111158000000
H	-6.902175000000	-4.124228000000	-2.929017000000
H	1.515629000000	-3.691242000000	-3.109595000000
C	-0.281148000000	-4.753765000000	-3.112172000000
N	0.359526000000	-5.981374000000	-3.102392000000
I	0.000000000000	0.000000000000	-2.120306000000
C	3.974945000000	-2.619240000000	3.117313000000
N	5.358408000000	-2.677682000000	3.102356000000
C	5.935449000000	-3.920270000000	3.016976000000
N	5.291080000000	-5.052846000000	3.022828000000
C	3.879017000000	-4.969207000000	3.081105000000
N	3.251110000000	-3.737166000000	3.096849000000
H	5.926508000000	-1.789882000000	3.083409000000
C	-0.428900000000	-7.102163000000	3.020999000000
O	3.188421000000	-6.018232000000	3.090229000000
H	2.381480000000	-1.363141000000	3.048124000000
N	3.391624000000	-1.419010000000	3.161231000000
H	3.951593000000	-0.532393000000	3.113590000000
H	7.022625000000	-3.914510000000	2.932320000000
N	-1.731930000000	-7.109353000000	3.025844000000
C	4.256737000000	2.132575000000	3.113173000000
N	4.999014000000	3.301502000000	3.099854000000
C	6.363532000000	3.179908000000	3.013056000000
N	7.021824000000	2.055409000000	3.017347000000
C	6.243594000000	0.874238000000	3.075309000000
N	4.862610000000	0.946732000000	3.091328000000
H	4.513849000000	4.237236000000	3.082967000000
C	-2.364954000000	-5.844320000000	3.079596000000
O	6.807203000000	-0.248210000000	3.084378000000
H	2.371435000000	1.382079000000	3.043052000000
N	2.925703000000	2.228291000000	3.157496000000
H	2.438468000000	3.156802000000	3.111158000000
H	6.902175000000	4.124228000000	2.929017000000
N	-1.611051000000	-4.684891000000	3.090734000000
C	0.281148000000	4.753765000000	3.112172000000
N	-0.359526000000	5.981374000000	3.102392000000
C	0.428900000000	7.102163000000	3.020999000000
N	1.731930000000	7.109353000000	3.025844000000
C	2.364954000000	5.844320000000	3.079596000000
N	1.611051000000	4.684891000000	3.090734000000
H	-1.412493000000	6.029320000000	3.085330000000

H	1.412493000000	-6.029320000000	3.085330000000
O	3.618703000000	5.769976000000	3.089401000000
H	-0.011612000000	2.746160000000	3.035619000000
N	-0.467504000000	3.649002000000	3.151292000000
H	-1.515629000000	3.691242000000	3.109595000000
H	-0.118836000000	8.041638000000	2.940630000000
H	0.118836000000	-8.041638000000	2.940630000000
C	-3.974945000000	2.619240000000	3.117313000000
N	-5.358408000000	2.677682000000	3.102356000000
C	-5.935449000000	3.920270000000	3.016976000000
N	-5.291080000000	5.052846000000	3.022828000000
C	-3.879017000000	4.969207000000	3.081105000000
N	-3.251110000000	3.737166000000	3.096849000000
H	-5.926508000000	1.789882000000	3.083409000000
O	-3.618703000000	-5.769976000000	3.089401000000
O	-3.188421000000	6.018232000000	3.090229000000
H	-2.381480000000	1.363141000000	3.048124000000
N	-3.391624000000	1.419010000000	3.161231000000
H	-3.951593000000	0.532393000000	3.113590000000
H	-7.022625000000	3.914510000000	2.932320000000
H	0.011612000000	-2.746160000000	3.035619000000
C	-4.256737000000	-2.132575000000	3.113173000000
N	-4.999014000000	-3.301502000000	3.099854000000
C	-6.363532000000	-3.179908000000	3.013056000000
N	-7.021824000000	-2.055409000000	3.017347000000
C	-6.243594000000	-0.874238000000	3.075309000000
N	-4.862610000000	-0.946732000000	3.091328000000
H	-4.513849000000	-4.237236000000	3.082967000000
N	0.467504000000	-3.649002000000	3.151292000000
O	-6.807203000000	0.248210000000	3.084378000000
H	-2.371435000000	-1.382079000000	3.043052000000
N	-2.925703000000	-2.228291000000	3.157496000000
H	-2.438468000000	-3.156802000000	3.111158000000
H	-6.902175000000	-4.124228000000	2.929017000000
H	1.515629000000	-3.691242000000	3.109595000000
C	-0.281148000000	-4.753765000000	3.112172000000
N	0.359526000000	-5.981374000000	3.102392000000
I	0.000000000000	0.000000000000	2.120306000000

(*C_1*)	*b*AM'$_6$-I$^-$-[*b*AM'$_6$]-I$^-$-*b*AM'$_6$		
C	-2.575975000000	-3.938507000000	0.000095000000
N	-2.619824000000	-5.319267000000	0.000107000000
C	-3.854557000000	-5.915399000000	0.000119000000
N	-4.994359000000	-5.282113000000	0.000119000000
C	-4.924267000000	-3.876054000000	0.000087000000
N	-3.703301000000	-3.229581000000	0.000065000000
H	-1.729436000000	-5.886344000000	0.000130000000
C	-7.052096000000	0.383345000000	0.000127000000
O	-5.981881000000	-3.194018000000	0.000081000000
H	-1.345370000000	-2.322082000000	0.000226000000
N	-1.384756000000	-3.336360000000	0.000118000000
H	-0.493160000000	-3.893829000000	0.000141000000
H	-3.835645000000	-7.004149000000	0.000135000000
N	-7.072427000000	1.687098000000	0.000125000000
C	2.122757000000	-4.203550000000	0.000064000000
N	3.296394000000	-4.932121000000	0.000064000000
C	3.194408000000	-6.299541000000	0.000071000000
N	2.075642000000	-6.969114000000	0.000079000000

C	0.893246000000	-6.205188000000	0.000076000000
N	0.944913000000	-4.824531000000	0.000072000000
H	4.232410000000	-4.444252000000	0.000078000000
C	-5.819604000000	2.328741000000	-0.000005000000
O	-0.226509000000	-6.779791000000	0.000077000000
H	1.339813000000	-2.328938000000	0.000142000000
N	2.198070000000	-2.870860000000	0.000051000000
H	3.126670000000	-2.377565000000	0.000065000000
H	4.146294000000	-6.828294000000	0.000071000000
N	-4.649721000000	1.593532000000	-0.000161000000
C	4.701177000000	-0.262840000000	-0.000043000000
N	5.919103000000	0.388955000000	-0.000036000000
C	7.052100000000	-0.383344000000	0.000026000000
N	7.072427000000	-1.687097000000	0.000072000000
C	5.819603000000	-2.328738000000	0.000052000000
N	4.649722000000	-1.593527000000	0.000002000000
H	5.964780000000	1.443509000000	-0.000063000000
H	-5.964774000000	-1.443506000000	0.000054000000
O	5.756580000000	-3.585653000000	0.000077000000
H	2.686399000000	-0.004386000000	-0.000093000000
N	3.584533000000	0.468508000000	-0.000096000000
H	3.621301000000	1.519529000000	-0.000164000000
H	7.986045000000	0.176512000000	0.000035000000
H	-7.986041000000	-0.176512000000	0.000221000000
C	2.575981000000	3.938511000000	-0.000152000000
N	2.619829000000	5.319271000000	0.000010000000
C	3.854561000000	5.915404000000	0.000147000000
N	4.994364000000	5.282120000000	0.000157000000
C	4.924274000000	3.876060000000	0.000015000000
N	3.703308000000	3.229586000000	-0.000165000000
H	1.729439000000	5.886346000000	0.000011000000
O	-5.756583000000	3.585656000000	0.000026000000
O	5.981889000000	3.194026000000	0.000054000000
H	1.345378000000	2.322084000000	-0.000552000000
N	1.384763000000	3.336362000000	-0.000281000000
H	0.493167000000	3.893830000000	-0.000291000000
H	3.835648000000	7.004154000000	0.000256000000
H	-2.686395000000	0.004394000000	-0.000269000000
C	-2.122755000000	4.203548000000	-0.000178000000
N	-3.296391000000	4.932120000000	0.000003000000
C	-3.194405000000	6.299540000000	0.000136000000
N	-2.075638000000	6.969112000000	0.000131000000
C	-0.893243000000	6.205186000000	-0.000033000000
N	-0.944910000000	4.824528000000	-0.000232000000
H	-4.232407000000	4.444253000000	0.000027000000
N	-3.584528000000	-0.468501000000	-0.000166000000
O	0.226513000000	6.779788000000	0.000003000000
H	-1.339812000000	2.328936000000	-0.000518000000
N	-2.198069000000	2.870858000000	-0.000287000000
H	-3.126671000000	2.377564000000	-0.000242000000
H	-4.146290000000	6.828293000000	0.000258000000
H	-3.621296000000	-1.519522000000	-0.000100000000
C	-4.701174000000	0.262845000000	-0.000104000000
N	-5.919098000000	-0.388952000000	0.000027000000
C	3.972273000000	-2.623348000000	-3.117114000000
N	5.355680000000	-2.683277000000	-3.102551000000
C	5.931401000000	-3.926517000000	-3.017735000000
N	5.285819000000	-5.058396000000	-3.023634000000
C	3.873825000000	-4.973224000000	-3.081243000000

N	3.247239000000	-3.740494000000	-3.096573000000
H	5.924755000000	-1.796099000000	-3.083565000000
C	-0.436509000000	-7.101651000000	-3.021716000000
O	3.182070000000	-6.021488000000	-3.090237000000
H	2.380181000000	-1.365546000000	-3.047384000000
N	3.390234000000	-1.422482000000	-3.160779000000
H	3.951175000000	-0.536481000000	-3.113071000000
H	7.018624000000	-3.921950000000	-2.933547000000
N	-1.739540000000	-7.107412000000	-3.026660000000
C	4.259149000000	2.128088000000	-3.112840000000
N	5.002663000000	3.296241000000	-3.100121000000
C	6.367099000000	3.173240000000	-3.013976000000
N	7.024212000000	2.048056000000	-3.018279000000
C	6.244713000000	0.867686000000	-3.075439000000
N	4.863800000000	0.941625000000	-3.090814000000
H	4.518478000000	4.232487000000	-3.083219000000
C	-2.371189000000	-5.841668000000	-3.079765000000
O	6.807132000000	-0.255364000000	-3.084455000000
H	2.373072000000	1.379576000000	-3.041990000000
N	2.928200000000	2.225172000000	-3.156802000000
H	2.441926000000	3.154190000000	-3.110676000000
H	6.906789000000	4.117028000000	-2.930542000000
N	-1.616021000000	-4.683056000000	-3.090364000000
C	0.286249000000	4.753529000000	-3.112033000000
N	-0.353088000000	5.981845000000	-3.102584000000
C	0.436575000000	7.101804000000	-3.021712000000
N	1.739606000000	7.107558000000	-3.026620000000
C	2.371249000000	5.841816000000	-3.079820000000
N	1.616074000000	4.683209000000	-3.090580000000
H	-1.405999000000	6.030932000000	-3.085462000000
H	1.406061000000	-6.030767000000	-3.085341000000
O	3.624924000000	5.766104000000	-3.089555000000
H	-0.008712000000	2.746255000000	-3.035139000000
N	-0.463628000000	3.649585000000	-3.150820000000
H	-1.511714000000	3.692961000000	-3.109289000000
H	-0.110128000000	8.041921000000	-2.941762000000
H	0.110198000000	-8.041772000000	-2.941842000000
C	-3.972239000000	2.623511000000	-3.117264000000
N	-5.355644000000	2.683446000000	-3.102589000000
C	-5.931351000000	3.926687000000	-3.017673000000
N	-5.285761000000	5.058562000000	-3.023566000000
C	-3.873772000000	4.973385000000	-3.081278000000
N	-3.247195000000	3.740651000000	-3.096742000000
H	-5.924717000000	1.796269000000	-3.083590000000
O	-3.624863000000	-5.765963000000	-3.089560000000
O	-3.182010000000	6.021644000000	-3.090243000000
H	-2.380149000000	1.365710000000	-3.047660000000
N	-3.390209000000	1.422641000000	-3.161019000000
H	-3.951146000000	0.536644000000	-3.113221000000
H	-7.018566000000	3.922124000000	-2.933404000000
H	0.008750000000	-2.746095000000	-3.034718000000
C	-4.259088000000	-2.127937000000	-3.112696000000
N	-5.002598000000	-3.296093000000	-3.099984000000
C	-6.367037000000	-3.173095000000	-3.013887000000
N	-7.024153000000	-2.047912000000	-3.018227000000
C	-6.244657000000	-0.867540000000	-3.075396000000
N	-4.863743000000	-0.941476000000	-3.090734000000
H	-4.518413000000	-4.232338000000	-3.083079000000
N	0.463675000000	-3.649414000000	-3.150433000000

O	-6.807078000000	0.255509000000	-3.084451000000
H	-2.373019000000	-1.379412000000	-3.041772000000
N	-2.928136000000	-2.225017000000	-3.156575000000
H	-2.441860000000	-3.154033000000	-3.110422000000
H	-6.906725000000	-4.116883000000	-2.930456000000
H	1.511764000000	-3.692789000000	-3.108938000000
C	-0.286195000000	-4.753366000000	-3.111791000000
N	0.353150000000	-5.981679000000	-3.102459000000
I	0.000041000000	0.000165000000	-2.119892000000
C	3.972319000000	-2.623328000000	3.117116000000
N	5.355730000000	-2.683198000000	3.102596000000
C	5.931506000000	-3.926418000000	3.017843000000
N	5.285973000000	-5.058325000000	3.023763000000
C	3.873974000000	-4.973211000000	3.081324000000
N	3.247334000000	-3.740507000000	3.096589000000
H	5.924767000000	-1.795998000000	3.083611000000
C	-0.436271000000	-7.101823000000	3.021965000000
O	3.182263000000	-6.021504000000	3.090337000000
H	2.380174000000	-1.365601000000	3.047291000000
N	3.390228000000	-1.422485000000	3.160727000000
H	3.951132000000	-0.536466000000	3.112964000000
H	7.018731000000	-3.921808000000	2.933692000000
N	-1.739302000000	-7.107640000000	3.026921000000
C	4.258983000000	2.128102000000	3.112597000000
N	5.002439000000	3.296294000000	3.100084000000
C	6.366895000000	3.173368000000	3.014152000000
N	7.024065000000	2.048218000000	3.018483000000
C	6.244618000000	0.867805000000	3.075459000000
N	4.863699000000	0.941672000000	3.090596000000
H	4.518215000000	4.232521000000	3.083220000000
C	-2.371004000000	-5.841921000000	3.079972000000
O	6.807092000000	-0.255218000000	3.084528000000
H	2.372951000000	1.379495000000	3.041401000000
N	2.928022000000	2.225114000000	3.156321000000
H	2.441712000000	3.154116000000	3.110238000000
H	6.906549000000	4.117190000000	2.930872000000
N	-1.615885000000	-4.683277000000	3.090519000000
C	0.285994000000	4.753348000000	3.111688000000
N	-0.353412000000	5.981631000000	3.102559000000
C	0.436192000000	7.101654000000	3.021993000000
N	1.739222000000	7.107482000000	3.026939000000
C	2.370933000000	5.841762000000	3.079843000000
N	1.615824000000	4.683110000000	3.090233000000
H	-1.406326000000	6.030676000000	3.085483000000
H	1.406254000000	-6.030859000000	3.085526000000
O	3.624611000000	5.766117000000	3.089660000000
H	-0.008856000000	2.746068000000	3.034319000000
N	-0.463821000000	3.649354000000	3.150174000000
H	-1.511911000000	3.692692000000	3.108742000000
H	-0.110561000000	8.041760000000	2.942276000000
H	0.110476000000	-8.041926000000	2.942132000000
C	-3.972365000000	2.623176000000	3.116950000000
N	-5.355777000000	2.683041000000	3.102552000000
C	-5.931565000000	3.926261000000	3.017894000000
N	-5.286037000000	5.058170000000	3.023811000000
C	-3.874033000000	4.973059000000	3.081260000000
N	-3.247386000000	3.740358000000	3.096391000000
H	-5.924815000000	1.795839000000	3.083582000000
O	-3.624681000000	-5.766267000000	3.089777000000

O	-3.182327000000	6.021355000000	3.090293000000
H	-2.380221000000	1.365450000000	3.046992000000
N	-3.390265000000	1.422335000000	3.160476000000
H	-3.951171000000	0.536311000000	3.112809000000
H	-7.018796000000	3.921649000000	2.933827000000
H	0.008806000000	-2.746248000000	3.034788000000
C	-4.259036000000	-2.128254000000	3.112735000000
N	-5.002496000000	-3.296444000000	3.100172000000
C	-6.366949000000	-3.173514000000	3.014214000000
N	-7.024115000000	-2.048362000000	3.018558000000
C	-6.244665000000	-0.867951000000	3.075558000000
N	-4.863746000000	-0.941821000000	3.090721000000
H	-4.518274000000	-4.232671000000	3.083261000000
N	0.463765000000	-3.649543000000	3.150564000000
O	-6.807137000000	0.255073000000	3.084624000000
H	-2.372998000000	-1.379659000000	3.041632000000
N	-2.928078000000	-2.225270000000	3.156528000000
H	-2.441769000000	-3.154274000000	3.110414000000
H	-6.906604000000	-4.117334000000	2.930902000000
H	1.511855000000	-3.692879000000	3.109037000000
C	-0.286056000000	-4.753529000000	3.111948000000
N	0.353340000000	-5.981815000000	3.102655000000
I	-0.000038000000	-0.000182000000	2.119540000000

(C_1) G			
N	-5.283451	-1.678255	3.537188
O	-1.838566	1.487985	3.513613
N	-3.617805	-0.001639	3.477698
C	-4.954082	-0.333853	3.401399
N	-5.918319	0.558896	3.265121
C	-5.449679	1.838702	3.227072
C	-4.124603	2.303105	3.285104
C	-3.054725	1.343360	3.427648
N	-6.220662	2.977069	3.103977
C	-5.340073	4.060135	3.095441
N	-4.079999	3.690717	3.202099
H	-4.633645	-2.348945	3.133182
H	-6.252487	-1.864377	3.287364
H	-2.919642	-0.724718	3.648190
H	-7.233853	2.999450	3.037783
H	-5.696649	5.080845	3.007543

(C_S) G_2			
N	-5.434325	-0.388049	0.000000
O	-1.423523	2.009203	0.000000
N	-3.460337	0.893064	0.000000
C	-4.838502	0.841259	0.000000
N	-5.615442	1.925547	0.000000
C	-4.898966	3.078973	0.000000
C	-3.505887	3.263254	0.000000
C	-2.652461	2.097114	0.000000
N	-5.426294	4.358399	0.000000
C	-4.342865	5.241504	0.000000
N	-3.182224	4.619056	0.000000
H	-4.941346	-1.282490	0.000000
H	-6.446210	-0.393457	0.000000
H	-2.921464	0.018105	0.000000

H	-6.415268	4.587070	0.000000
H	-4.487509	6.316770	0.000000
N	0.489948	-5.609031	0.000000
O	-1.883226	-1.595387	0.000000
N	-0.787225	-3.631420	0.000000
C	-0.741755	-5.014766	0.000000
N	-1.826145	-5.779307	0.000000
C	-2.978093	-5.060532	0.000000
C	-3.145701	-3.667185	0.000000
C	-1.983849	-2.824873	0.000000
N	-4.263420	-5.569290	0.000000
C	-5.133589	-4.480925	0.000000
N	-4.493656	-3.327170	0.000000
H	1.352950	-5.083115	0.000000
H	0.527107	-6.620713	0.000000
H	0.071385	-3.080809	0.000000
H	-4.509920	-6.554774	0.000000
H	-6.210041	-4.610173	0.000000

(C_S)	G_4		
N	-5.491538	-0.443911	0.000000
O	-1.491209	1.945649	0.000000
N	-3.511074	0.816438	0.000000
C	-4.894338	0.771924	0.000000
N	-5.673775	1.866442	0.000000
C	-4.961559	3.010988	0.000000
C	-3.568888	3.175153	0.000000
C	-2.733945	2.007272	0.000000
N	-5.468128	4.303204	0.000000
C	-4.380008	5.171353	0.000000
N	-3.227720	4.525318	0.000000
H	-5.009867	-1.357566	0.000000
H	-6.504174	-0.436158	0.000000
H	-2.968063	-0.075901	0.000000
H	-6.452707	4.550770	0.000000
H	-4.504995	6.248183	0.000000
N	0.443911	-5.491539	0.000000
O	-1.945649	-1.491210	0.000000
N	-0.816438	-3.511074	0.000000
C	-0.771924	-4.894338	0.000000
N	-1.866442	-5.673776	0.000000
C	-3.010988	-4.961559	0.000000
C	-3.175153	-3.568889	0.000000
C	-2.007272	-2.733946	0.000000
N	-4.303204	-5.468129	0.000000
C	-5.171353	-4.380008	0.000000
N	-4.525318	-3.227720	0.000000
H	1.357566	-5.009867	0.000000
H	0.436158	-6.504175	0.000000
H	0.075901	-2.968064	0.000000
H	-4.550770	-6.452707	0.000000
H	-6.248183	-4.504995	0.000000
N	5.491538	0.443911	0.000000
O	1.491209	-1.945649	0.000000
N	3.511074	-0.816438	0.000000
C	4.894338	-0.771924	0.000000
N	5.673775	-1.866442	0.000000
C	4.961559	-3.010988	0.000000

C	3.568888	-3.175153	0.000000
C	2.733945	-2.007272	0.000000
N	5.468128	-4.303204	0.000000
C	4.380008	-5.171353	0.000000
N	3.227720	-4.525318	0.000000
H	5.009867	1.357566	0.000000
H	6.504174	0.436158	0.000000
H	2.968063	0.075901	0.000000
H	6.452707	-4.550770	0.000000
H	4.504995	-6.248183	0.000000
N	-0.443911	5.491539	0.000000
O	1.945649	1.491210	0.000000
N	0.816438	3.511074	0.000000
C	0.771924	4.894338	0.000000
N	1.866442	5.673776	0.000000
C	3.010988	4.961559	0.000000
C	3.175153	3.568889	0.000000
C	2.007272	2.733946	0.000000
N	4.303204	5.468129	0.000000
C	5.171353	4.380008	0.000000
N	4.525318	3.227720	0.000000
H	-1.357566	5.009867	0.000000
H	-0.436158	6.504175	0.000000
H	-0.075901	2.968064	0.000000
H	4.550770	6.452707	0.000000
H	6.248183	4.504995	0.000000

(C_1) G_4

N	1.206021	-5.106327	-1.152831
O	-1.591149	-1.755290	0.461615
N	-0.262258	-3.470004	-0.338717
C	-0.065867	-4.751306	-0.819035
N	-1.057814	-5.636415	-1.001629
C	-2.268887	-5.131359	-0.684437
C	-2.583365	-3.857636	-0.188623
C	-1.518397	-2.918328	0.022251
N	-3.489833	-5.784698	-0.773145
C	-4.466258	-4.892855	-0.338279
N	-3.954705	-3.728689	0.020891
H	2.032288	-4.626466	-0.758876
H	1.319240	-6.087811	-1.382729
H	0.544011	-2.801996	-0.322381
H	-3.627628	-6.735608	-1.100930
H	-5.516328	-5.160876	-0.310897
N	5.107172	1.206051	1.151728
O	1.755031	-1.590833	-0.460900
N	3.470284	-0.262099	0.338527
C	4.751933	-0.065784	0.817947
N	5.637187	-1.057755	0.999699
C	5.131927	-2.268767	0.682595
C	3.857856	-2.583159	0.187622
C	2.918376	-1.518165	-0.022344
N	5.785362	-3.489717	0.770536
C	4.893233	-4.466060	0.336075
N	3.728796	-3.954451	-0.022137
H	4.627065	2.032359	0.758152
H	6.088831	1.319233	1.380906
H	2.802255	0.544168	0.322790

H	6.736516	-3.627562	1.097593
H	5.161260	-5.516117	0.308250
N	-1.206202	5.106545	-1.151407
O	1.591161	1.755185	0.462042
N	0.262174	3.470059	-0.337799
C	0.065724	4.751468	-0.817811
N	1.057644	5.636627	-1.000302
C	2.268752	5.131514	-0.683335
C	2.583288	3.857688	-0.187824
C	1.518350	2.918319	0.022941
N	3.489682	5.784882	-0.772019
C	4.466157	4.892957	-0.337437
N	3.954648	3.728710	0.021533
H	-2.032413	4.626620	-0.757414
H	-1.319448	6.088081	-1.381073
H	-0.544084	2.802035	-0.321584
H	3.627436	6.735863	-1.099617
H	5.516228	5.160979	-0.310101
N	-5.106981	-1.206350	1.152129
O	-1.755167	1.590964	-0.460431
N	-3.470261	0.262017	0.338983
C	-4.751794	0.065583	0.818665
N	-5.636974	1.057519	1.000966
C	-5.131760	2.268620	0.684126
C	-3.857812	2.583135	0.188914
C	-2.918410	1.518182	-0.021620
N	-5.785130	3.489562	0.772650
C	-4.893091	4.466019	0.338259
N	-3.728763	3.954491	-0.020427
H	-4.626995	-2.032538	0.758156
H	-6.088586	-1.319582	1.381513
H	-2.802249	-0.544257	0.322859
H	-6.736185	3.627334	1.100023
H	-5.161093	5.516093	0.310846

(C_{2h}) G_4–[G_4]–G_4

N	-2.443908000000	4.898351000000	3.306218000000
O	-2.428781000000	0.258563000000	3.248190000000
N	-2.505813000000	2.564968000000	3.207142000000
C	-3.180965000000	3.767238000000	3.227107000000
N	-4.517370000000	3.870459000000	3.202556000000
C	-5.126202000000	2.669405000000	3.197727000000
C	-4.548447000000	1.395610000000	3.222049000000
C	-3.118862000000	1.286062000000	3.228196000000
N	-6.488569000000	2.433120000000	3.179634000000
C	-6.666176000000	1.056319000000	3.197098000000
N	-5.522435000000	0.406841000000	3.227108000000
H	-1.416072000000	4.944878000000	3.234071000000
H	-2.960097000000	5.759547000000	3.191280000000
H	-1.462232000000	2.565191000000	3.202584000000
H	-7.207864000000	3.144765000000	3.162897000000
H	-7.651276000000	0.610108000000	3.185428000000
N	4.898351000000	2.443908000000	3.306218000000
O	0.258563000000	2.428781000000	3.248190000000
N	2.564968000000	2.505813000000	3.207142000000
C	3.767238000000	3.180965000000	3.227107000000
N	3.870459000000	4.517370000000	3.202556000000
C	2.669405000000	5.126202000000	3.197727000000

C	1.395610000000	4.548447000000	3.222049000000
C	1.286062000000	3.118862000000	3.228196000000
N	2.433120000000	6.488569000000	3.179634000000
C	1.056319000000	6.666176000000	3.197098000000
N	0.406841000000	5.522435000000	3.227108000000
H	4.944878000000	1.416072000000	3.234071000000
H	5.759547000000	2.960097000000	3.191280000000
H	2.565191000000	1.462232000000	3.202584000000
H	3.144765000000	7.207864000000	3.162897000000
H	0.610108000000	7.651276000000	3.185428000000
N	2.443908000000	-4.898351000000	3.306218000000
O	2.428781000000	-0.258563000000	3.248190000000
N	2.505813000000	-2.564968000000	3.207142000000
C	3.180965000000	-3.767238000000	3.227107000000
N	4.517370000000	-3.870459000000	3.202556000000
C	5.126202000000	-2.669405000000	3.197727000000
C	4.548447000000	-1.395610000000	3.222049000000
C	3.118862000000	-1.286062000000	3.228196000000
N	6.488569000000	-2.433120000000	3.179634000000
C	6.666176000000	-1.056319000000	3.197098000000
N	5.522435000000	-0.406841000000	3.227108000000
H	1.416072000000	-4.944878000000	3.234071000000
H	2.960097000000	-5.759547000000	3.191280000000
H	1.462232000000	-2.565191000000	3.202584000000
H	7.207864000000	-3.144765000000	3.162897000000
H	7.651276000000	-0.610108000000	3.185428000000
N	-4.898351000000	-2.443908000000	3.306218000000
O	-0.258563000000	-2.428781000000	3.248190000000
N	-2.564968000000	-2.505813000000	3.207142000000
C	-3.767238000000	-3.180965000000	3.227107000000
N	-3.870459000000	-4.517370000000	3.202556000000
C	-2.669405000000	-5.126202000000	3.197727000000
C	-1.395610000000	-4.548447000000	3.222049000000
C	-1.286062000000	-3.118862000000	3.228196000000
N	-2.433120000000	-6.488569000000	3.179634000000
C	-1.056319000000	-6.666176000000	3.197098000000
N	-0.406841000000	-5.522435000000	3.227108000000
H	-4.944878000000	-1.416072000000	3.234071000000
H	-5.759547000000	-2.960097000000	3.191280000000
H	-2.565191000000	-1.462232000000	3.202584000000
H	-3.144765000000	-7.207864000000	3.162897000000
H	-0.610108000000	-7.651276000000	3.185428000000
N	-4.586608000000	2.940657000000	0.000000000000
O	-2.219099000000	-1.041988000000	0.000000000000
N	-3.456403000000	0.903448000000	0.000000000000
C	-4.649836000000	1.594877000000	0.000000000000
N	-5.856113000000	1.005155000000	0.000000000000
C	-5.766409000000	-0.335568000000	0.000000000000
C	-4.620824000000	-1.137836000000	0.000000000000
C	-3.332999000000	-0.505311000000	0.000000000000
N	-6.818617000000	-1.235037000000	0.000000000000
C	-6.266591000000	-2.514660000000	0.000000000000
N	-4.951805000000	-2.482507000000	0.000000000000
H	-3.727082000000	3.508232000000	0.000000000000
H	-5.469952000000	3.429292000000	0.000000000000
H	-2.559404000000	1.437491000000	0.000000000000
H	-7.800115000000	-0.989203000000	0.000000000000
H	-6.883136000000	-3.403756000000	0.000000000000
N	2.940657000000	4.586608000000	0.000000000000

O	-1.041988000000	2.219099000000	0.000000000000
N	0.903448000000	3.456403000000	0.000000000000
C	1.594877000000	4.649836000000	0.000000000000
N	1.005155000000	5.856113000000	0.000000000000
C	-0.335568000000	5.766409000000	0.000000000000
C	-1.137836000000	4.620824000000	0.000000000000
C	-0.505311000000	3.332999000000	0.000000000000
N	-1.235037000000	6.818617000000	0.000000000000
C	-2.514660000000	6.266591000000	0.000000000000
N	-2.482507000000	4.951805000000	0.000000000000
H	3.508232000000	3.727082000000	0.000000000000
H	3.429292000000	5.469952000000	0.000000000000
H	1.437491000000	2.559404000000	0.000000000000
H	-0.989203000000	7.800115000000	0.000000000000
H	-3.403756000000	6.883136000000	0.000000000000
N	4.586608000000	-2.940657000000	0.000000000000
O	2.219099000000	1.041988000000	0.000000000000
N	3.456403000000	-0.903448000000	0.000000000000
C	4.649836000000	-1.594877000000	0.000000000000
N	5.856113000000	-1.005155000000	0.000000000000
C	5.766409000000	0.335568000000	0.000000000000
C	4.620824000000	1.137836000000	0.000000000000
C	3.332999000000	0.505311000000	0.000000000000
N	6.818617000000	1.235037000000	0.000000000000
C	6.266591000000	2.514660000000	0.000000000000
N	4.951805000000	2.482507000000	0.000000000000
H	3.727082000000	-3.508232000000	0.000000000000
H	5.469952000000	-3.429292000000	0.000000000000
H	2.559404000000	-1.437491000000	0.000000000000
H	7.800115000000	0.989203000000	0.000000000000
H	6.883136000000	3.403756000000	0.000000000000
N	-2.940657000000	-4.586608000000	0.000000000000
O	1.041988000000	-2.219099000000	0.000000000000
N	-0.903448000000	-3.456403000000	0.000000000000
C	-1.594877000000	-4.649836000000	0.000000000000
N	-1.005155000000	-5.856113000000	0.000000000000
C	0.335568000000	-5.766409000000	0.000000000000
C	1.137836000000	-4.620824000000	0.000000000000
C	0.505311000000	-3.332999000000	0.000000000000
N	1.235037000000	-6.818617000000	0.000000000000
C	2.514660000000	-6.266591000000	0.000000000000
N	2.482507000000	-4.951805000000	0.000000000000
H	-3.508232000000	-3.727082000000	0.000000000000
H	-3.429292000000	-5.469952000000	0.000000000000
H	-1.437491000000	-2.559404000000	0.000000000000
H	0.989203000000	-7.800115000000	0.000000000000
H	3.403756000000	-6.883136000000	0.000000000000
N	-2.443908000000	4.898351000000	-3.306218000000
O	-2.428781000000	0.258563000000	-3.248190000000
N	-2.505813000000	2.564968000000	-3.207142000000
C	-3.180965000000	3.767238000000	-3.227107000000
N	-4.517370000000	3.870459000000	-3.202556000000
C	-5.126202000000	2.669405000000	-3.197727000000
C	-4.548447000000	1.395610000000	-3.222049000000
C	-3.118862000000	1.286062000000	-3.228196000000
N	-6.488569000000	2.433120000000	-3.179634000000
C	-6.666176000000	1.056319000000	-3.197098000000
N	-5.522435000000	0.406841000000	-3.227108000000
H	-1.416072000000	4.944878000000	-3.234071000000

H	-2.960097000000	5.759547000000	-3.191280000000
H	-1.462232000000	2.565191000000	-3.202584000000
H	-7.207864000000	3.144765000000	-3.162897000000
H	-7.651276000000	0.610108000000	-3.185428000000
N	4.898351000000	2.443908000000	-3.306218000000
O	0.258563000000	2.428781000000	-3.248190000000
N	2.564968000000	2.505813000000	-3.207142000000
C	3.767238000000	3.180965000000	-3.227107000000
N	3.870459000000	4.517370000000	-3.202556000000
C	2.669405000000	5.126202000000	-3.197727000000
C	1.395610000000	4.548447000000	-3.222049000000
C	1.286062000000	3.118862000000	-3.228196000000
N	2.433120000000	6.488569000000	-3.179634000000
C	1.056319000000	6.666176000000	-3.197098000000
N	0.406841000000	5.522435000000	-3.227108000000
H	4.944878000000	1.416072000000	-3.234071000000
H	5.759547000000	2.960097000000	-3.191280000000
H	2.565191000000	1.462232000000	-3.202584000000
H	3.144765000000	7.207864000000	-3.162897000000
H	0.610108000000	7.651276000000	-3.185428000000
N	2.443908000000	-4.898351000000	-3.306218000000
O	2.428781000000	-0.258563000000	-3.248190000000
N	2.505813000000	-2.564968000000	-3.207142000000
C	3.180965000000	-3.767238000000	-3.227107000000
N	4.517370000000	-3.870459000000	-3.202556000000
C	5.126202000000	-2.669405000000	-3.197727000000
C	4.548447000000	-1.395610000000	-3.222049000000
C	3.118862000000	-1.286062000000	-3.228196000000
N	6.488569000000	-2.433120000000	-3.179634000000
C	6.666176000000	-1.056319000000	-3.197098000000
N	5.522435000000	-0.406841000000	-3.227108000000
H	1.416072000000	-4.944878000000	-3.234071000000
H	2.960097000000	-5.759547000000	-3.191280000000
H	1.462232000000	-2.565191000000	-3.202584000000
H	7.207864000000	-3.144765000000	-3.162897000000
H	7.651276000000	-0.610108000000	-3.185428000000
N	-4.898351000000	-2.443908000000	-3.306218000000
O	-0.258563000000	-2.428781000000	-3.248190000000
N	-2.564968000000	-2.505813000000	-3.207142000000
C	-3.767238000000	-3.180965000000	-3.227107000000
N	-3.870459000000	-4.517370000000	-3.202556000000
C	-2.669405000000	-5.126202000000	-3.197727000000
C	-1.395610000000	-4.548447000000	-3.222049000000
C	-1.286062000000	-3.118862000000	-3.228196000000
N	-2.433120000000	-6.488569000000	-3.179634000000
C	-1.056319000000	-6.666176000000	-3.197098000000
N	-0.406841000000	-5.522435000000	-3.227108000000
H	-4.944878000000	-1.416072000000	-3.234071000000
H	-5.759547000000	-2.960097000000	-3.191280000000
H	-2.565191000000	-1.462232000000	-3.202584000000
H	-3.144765000000	-7.207864000000	-3.162897000000
H	-0.610108000000	-7.651276000000	-3.185428000000

(C_{2h})	G_4-Na^+-[G_4]-Na^+-G_4		
N	-2.970311000000	4.688173000000	3.196873000000
O	-2.242989000000	0.121258000000	2.755185000000
N	-2.669371000000	2.377541000000	3.010184000000
C	-3.517899000000	3.458115000000	3.199514000000

N	-4.839541000000	3.337362000000	3.361465000000
C	-5.251215000000	2.062137000000	3.340121000000
C	-4.486518000000	0.900097000000	3.162370000000
C	-3.079380000000	1.030912000000	2.962862000000
N	-6.550802000000	1.614880000000	3.476847000000
C	-6.518548000000	0.232716000000	3.387990000000
N	-5.299452000000	-0.223261000000	3.195605000000
H	-1.943202000000	4.856430000000	3.181829000000
H	-3.592143000000	5.440513000000	3.463784000000
H	-1.641701000000	2.519581000000	2.916939000000
H	-7.355309000000	2.197908000000	3.676692000000
H	-7.414064000000	-0.367494000000	3.474889000000
N	4.688173000000	2.970311000000	3.196873000000
O	0.121258000000	2.242989000000	2.755185000000
N	2.377541000000	2.669371000000	3.010184000000
C	3.458115000000	3.517899000000	3.199514000000
N	3.337362000000	4.839541000000	3.361465000000
C	2.062137000000	5.251215000000	3.340121000000
C	0.900097000000	4.486518000000	3.162370000000
C	1.030912000000	3.079380000000	2.962862000000
N	1.614880000000	6.550802000000	3.476847000000
C	0.232716000000	6.518548000000	3.387990000000
N	-0.223261000000	5.299452000000	3.195605000000
H	4.856430000000	1.943202000000	3.181829000000
H	5.440513000000	3.592143000000	3.463784000000
H	2.519581000000	1.641701000000	2.916939000000
H	2.197908000000	7.355309000000	3.676692000000
H	-0.367494000000	7.414064000000	3.474889000000
N	2.970311000000	-4.688173000000	3.196873000000
O	2.242989000000	-0.121258000000	2.755185000000
N	2.669371000000	-2.377541000000	3.010184000000
C	3.517899000000	-3.458115000000	3.199514000000
N	4.839541000000	-3.337362000000	3.361465000000
C	5.251215000000	-2.062137000000	3.340121000000
C	4.486518000000	-0.900097000000	3.162370000000
C	3.079380000000	-1.030912000000	2.962862000000
N	6.550802000000	-1.614880000000	3.476847000000
C	6.518548000000	-0.232716000000	3.387990000000
N	5.299452000000	0.223261000000	3.195605000000
H	1.943202000000	-4.856430000000	3.181829000000
H	3.592143000000	-5.440513000000	3.463784000000
H	1.641701000000	-2.519581000000	2.916939000000
H	7.355309000000	-2.197908000000	3.676692000000
H	7.414064000000	0.367494000000	3.474889000000
N	-4.688173000000	-2.970311000000	3.196873000000
O	-0.121258000000	-2.242989000000	2.755185000000
N	-2.377541000000	-2.669371000000	3.010184000000
C	-3.458115000000	-3.517899000000	3.199514000000
N	-3.337362000000	-4.839541000000	3.361465000000
C	-2.062137000000	-5.251215000000	3.340121000000
C	-0.900097000000	-4.486518000000	3.162370000000
C	-1.030912000000	-3.079380000000	2.962862000000
N	-1.614880000000	-6.550802000000	3.476847000000
C	-0.232716000000	-6.518548000000	3.387990000000
N	0.223261000000	-5.299452000000	3.195605000000
H	-4.856430000000	-1.943202000000	3.181829000000
H	-5.440513000000	-3.592143000000	3.463784000000
H	-2.519581000000	-1.641701000000	2.916939000000
H	-2.197908000000	-7.355309000000	3.676692000000

H	0.367494000000	-7.414064000000	3.474889000000
N	-5.476892000000	1.169173000000	0.000000000000
O	-1.610493000000	-1.393092000000	0.000000000000
N	-3.580079000000	-0.183609000000	0.000000000000
C	-4.962121000000	-0.071176000000	0.000000000000
N	-5.784039000000	-1.125378000000	0.000000000000
C	-5.127882000000	-2.291666000000	0.000000000000
C	-3.742815000000	-2.517419000000	0.000000000000
C	-2.869620000000	-1.393030000000	0.000000000000
N	-5.692752000000	-3.550739000000	0.000000000000
C	-4.655107000000	-4.464082000000	0.000000000000
N	-3.477374000000	-3.879591000000	0.000000000000
H	-4.876488000000	2.019086000000	0.000000000000
H	-6.484947000000	1.237424000000	0.000000000000
H	-2.970038000000	0.656410000000	0.000000000000
H	-6.685344000000	-3.751447000000	0.000000000000
H	-4.831348000000	-5.530816000000	0.000000000000
N	1.169173000000	5.476892000000	0.000000000000
O	-1.393092000000	1.610493000000	0.000000000000
N	-0.183609000000	3.580079000000	0.000000000000
C	-0.071176000000	4.962121000000	0.000000000000
N	-1.125378000000	5.784039000000	0.000000000000
C	-2.291666000000	5.127882000000	0.000000000000
C	-2.517419000000	3.742815000000	0.000000000000
C	-1.393030000000	2.869620000000	0.000000000000
N	-3.550739000000	5.692752000000	0.000000000000
C	-4.464082000000	4.655107000000	0.000000000000
N	-3.879591000000	3.477374000000	0.000000000000
H	2.019086000000	4.876488000000	0.000000000000
H	1.237424000000	6.484947000000	0.000000000000
H	0.656410000000	2.970038000000	0.000000000000
H	-3.751447000000	6.685344000000	0.000000000000
H	-5.530816000000	4.831348000000	0.000000000000
N	5.476892000000	-1.169173000000	0.000000000000
O	1.610493000000	1.393092000000	0.000000000000
N	3.580079000000	0.183609000000	0.000000000000
C	4.962121000000	0.071176000000	0.000000000000
N	5.784039000000	1.125378000000	0.000000000000
C	5.127882000000	2.291666000000	0.000000000000
C	3.742815000000	2.517419000000	0.000000000000
C	2.869620000000	1.393030000000	0.000000000000
N	5.692752000000	3.550739000000	0.000000000000
C	4.655107000000	4.464082000000	0.000000000000
N	3.477374000000	3.879591000000	0.000000000000
H	4.876488000000	-2.019086000000	0.000000000000
H	6.484947000000	-1.237424000000	0.000000000000
H	2.970038000000	-0.656410000000	0.000000000000
H	6.685344000000	3.751447000000	0.000000000000
H	4.831348000000	5.530816000000	0.000000000000
N	-1.169173000000	-5.476892000000	0.000000000000
O	1.393092000000	-1.610493000000	0.000000000000
N	0.183609000000	-3.580079000000	0.000000000000
C	0.071176000000	-4.962121000000	0.000000000000
N	1.125378000000	-5.784039000000	0.000000000000
C	2.291666000000	-5.127882000000	0.000000000000
C	2.517419000000	-3.742815000000	0.000000000000
C	1.393030000000	-2.869620000000	0.000000000000
N	3.550739000000	-5.692752000000	0.000000000000
C	4.464082000000	-4.655107000000	0.000000000000

N	3.879591000000	-3.477374000000	0.000000000000
H	-2.019086000000	-4.876488000000	0.000000000000
H	-1.237424000000	-6.484947000000	0.000000000000
H	-0.656410000000	-2.970038000000	0.000000000000
H	3.751447000000	-6.685344000000	0.000000000000
H	5.530816000000	-4.831348000000	0.000000000000
N	-2.970311000000	4.688173000000	-3.196873000000
O	-2.242989000000	0.121258000000	-2.755185000000
N	-2.669371000000	2.377541000000	-3.010184000000
C	-3.517899000000	3.458115000000	-3.199514000000
N	-4.839541000000	3.337362000000	-3.361465000000
C	-5.251215000000	2.062137000000	-3.340121000000
C	-4.486518000000	0.900097000000	-3.162370000000
C	-3.079380000000	1.030912000000	-2.962862000000
N	-6.550802000000	1.614880000000	-3.476847000000
C	-6.518548000000	0.232716000000	-3.387990000000
N	-5.299452000000	-0.223261000000	-3.195605000000
H	-1.943202000000	4.856430000000	-3.181829000000
H	-3.592143000000	5.440513000000	-3.463784000000
H	-1.641701000000	2.519581000000	-2.916939000000
H	-7.355309000000	2.197908000000	-3.676692000000
H	-7.414064000000	-0.367494000000	-3.474889000000
N	4.688173000000	2.970311000000	-3.196873000000
O	0.121258000000	2.242989000000	-2.755185000000
N	2.377541000000	2.669371000000	-3.010184000000
C	3.458115000000	3.517899000000	-3.199514000000
N	3.337362000000	4.839541000000	-3.361465000000
C	2.062137000000	5.251215000000	-3.340121000000
C	0.900097000000	4.486518000000	-3.162370000000
C	1.030912000000	3.079380000000	-2.962862000000
N	1.614880000000	6.550802000000	-3.476847000000
C	0.232716000000	6.518548000000	-3.387990000000
N	-0.223261000000	5.299452000000	-3.195605000000
H	4.856430000000	1.943202000000	-3.181829000000
H	5.440513000000	3.592143000000	-3.463784000000
H	2.519581000000	1.641701000000	-2.916939000000
H	2.197908000000	7.355309000000	-3.676692000000
H	-0.367494000000	7.414064000000	-3.474889000000
N	2.970311000000	-4.688173000000	-3.196873000000
O	2.242989000000	-0.121258000000	-2.755185000000
N	2.669371000000	-2.377541000000	-3.010184000000
C	3.517899000000	-3.458115000000	-3.199514000000
N	4.839541000000	-3.337362000000	-3.361465000000
C	5.251215000000	-2.062137000000	-3.340121000000
C	4.486518000000	-0.900097000000	-3.162370000000
C	3.079380000000	-1.030912000000	-2.962862000000
N	6.550802000000	-1.614880000000	-3.476847000000
C	6.518548000000	-0.232716000000	-3.387990000000
N	5.299452000000	0.223261000000	-3.195605000000
H	1.943202000000	-4.856430000000	-3.181829000000
H	3.592143000000	-5.440513000000	-3.463784000000
H	1.641701000000	-2.519581000000	-2.916939000000
H	7.355309000000	-2.197908000000	-3.676692000000
H	7.414064000000	0.367494000000	-3.474889000000
N	-4.688173000000	-2.970311000000	-3.196873000000
O	-0.121258000000	-2.242989000000	-2.755185000000
N	-2.377541000000	-2.669371000000	-3.010184000000
C	-3.458115000000	-3.517899000000	-3.199514000000
N	-3.337362000000	-4.839541000000	-3.361465000000

C	-2.062137000000	-5.251215000000	-3.340121000000
C	-0.900097000000	-4.486518000000	-3.162370000000
C	-1.030912000000	-3.079380000000	-2.962862000000
N	-1.614880000000	-6.550802000000	-3.476847000000
C	-0.232716000000	-6.518548000000	-3.387990000000
N	0.223261000000	-5.299452000000	-3.195605000000
H	-4.856430000000	-1.943202000000	-3.181829000000
H	-5.440513000000	-3.592143000000	-3.463784000000
H	-2.519581000000	-1.641701000000	-2.916939000000
H	-2.197908000000	-7.355309000000	-3.676692000000
H	0.367494000000	-7.414064000000	-3.474889000000
Na	0.000000000000	0.000000000000	-1.560535000000
Na	0.000000000000	0.000000000000	1.560535000000

(C_{2h})	G_4-K^+-[G_4]-K^+-G_4		
N	4.088554290000	-3.745654670000	-3.350738650000
O	-0.330073790000	-2.337022920000	-3.074722220000
N	1.843301190000	-3.101784860000	-3.230630040000
C	2.789946560000	-4.105343610000	-3.345343590000
N	2.474508590000	-5.402408660000	-3.436987200000
C	1.150861060000	-5.625160340000	-3.424047550000
C	0.113117600000	-4.686075530000	-3.318300470000
C	0.449187850000	-3.309951210000	-3.201706720000
N	0.510014390000	-6.849185100000	-3.502435330000
C	-0.857208070000	-6.604680530000	-3.453977380000
N	-1.126911230000	-5.319791930000	-3.339085680000
H	4.436158290000	-2.771037890000	-3.342154820000
H	4.745474080000	-4.491954770000	-3.532774060000
H	2.150146730000	-2.109834480000	-3.189990340000
H	0.965357550000	-7.742257090000	-3.648660990000
H	-1.583190070000	-7.402978630000	-3.508995330000
N	3.745654670000	4.088554290000	-3.350738650000
O	2.337022920000	-0.330073790000	-3.074722220000
N	3.101784860000	1.843301190000	-3.230630040000
C	4.105343610000	2.789946560000	-3.345343590000
N	5.402408660000	2.474508590000	-3.436987200000
C	5.625160340000	1.150861060000	-3.424047550000
C	4.686075530000	0.113117600000	-3.318300470000
C	3.309951210000	0.449187850000	-3.201706720000
N	6.849185100000	0.510014390000	-3.502435330000
C	6.604680530000	-0.857208070000	-3.453977380000
N	5.319791930000	-1.126911230000	-3.339085680000
H	2.771037890000	4.436158290000	-3.342154820000
H	4.491954770000	4.745474080000	-3.532774060000
H	2.109834480000	2.150146730000	-3.189990340000
H	7.742257090000	0.965357550000	-3.648660990000
H	7.402978630000	-1.583190070000	-3.508995330000
N	-4.088554290000	3.745654670000	-3.350738650000
O	0.330073790000	2.337022920000	-3.074722220000
N	-1.843301190000	3.101784860000	-3.230630040000
C	-2.789946560000	4.105343610000	-3.345343590000
N	-2.474508590000	5.402408660000	-3.436987200000
C	-1.150861060000	5.625160340000	-3.424047550000
C	-0.113117600000	4.686075530000	-3.318300470000
C	-0.449187850000	3.309951210000	-3.201706720000
N	-0.510014390000	6.849185100000	-3.502435330000
C	0.857208070000	6.604680530000	-3.453977380000
N	1.126911230000	5.319791930000	-3.339085680000

H	-4.436158290000	2.771037890000	-3.342154820000
H	-4.745474080000	4.491954770000	-3.532774060000
H	-2.150146730000	2.109834480000	-3.189990340000
H	-0.965357550000	7.742257090000	-3.648660990000
H	1.583190070000	7.402978630000	-3.508995330000
N	-3.745654670000	-4.088554290000	-3.350738650000
O	-2.337022920000	0.330073790000	-3.074722220000
N	-3.101784860000	-1.843301190000	-3.230630040000
C	-4.105343610000	-2.789946560000	-3.345343590000
N	-5.402408660000	-2.474508590000	-3.436987200000
C	-5.625160340000	-1.150861060000	-3.424047550000
C	-4.686075530000	-0.113117600000	-3.318300470000
C	-3.309951210000	-0.449187850000	-3.201706720000
N	-6.849185100000	-0.510014390000	-3.502435330000
C	-6.604680530000	0.857208070000	-3.453977380000
N	-5.319791930000	1.126911230000	-3.339085680000
H	-2.771037890000	-4.436158290000	-3.342154820000
H	-4.491954770000	-4.745474080000	-3.532774060000
H	-2.109834480000	-2.150146730000	-3.189990340000
H	-7.742257090000	-0.965357550000	-3.648660990000
H	-7.402978630000	1.583190070000	-3.508995330000
N	3.766549570000	4.069670750000	3.348867160000
O	2.335552450000	-0.341880740000	3.073929080000
N	3.111308360000	1.827641210000	3.229405750000
C	4.119602550000	2.769201030000	3.344231160000
N	5.414992660000	2.447192110000	3.436555680000
C	5.630984410000	1.122398570000	3.424683020000
C	4.686666440000	0.089400000000	3.319056770000
C	3.312370490000	0.432438580000	3.201323150000
N	6.851681060000	0.475390730000	3.504045910000
C	6.600269610000	-0.890586770000	3.456055740000
N	5.314089680000	-1.153830450000	3.340620220000
H	2.793620680000	4.422011580000	3.341854050000
H	4.515985130000	4.722597540000	3.532418130000
H	2.120971590000	2.139553040000	3.188376950000
H	7.747017530000	0.926288560000	3.650186000000
H	7.394883360000	-1.620555110000	3.511774130000
N	-4.069670750000	3.766549570000	3.348867160000
O	0.341880740000	2.335552450000	3.073929080000
N	-1.827641210000	3.111308360000	3.229405750000
C	-2.769201030000	4.119602550000	3.344231160000
N	-2.447192110000	5.414992660000	3.436555680000
C	-1.122398570000	5.630984410000	3.424683020000
C	-0.089400000000	4.686666440000	3.319056770000
C	-0.432438580000	3.312370490000	3.201323150000
N	-0.475390730000	6.851681060000	3.504045910000
C	0.890586770000	6.600269610000	3.456055740000
N	1.153830450000	5.314089680000	3.340620220000
H	-4.422011580000	2.793620680000	3.341854050000
H	-4.722597540000	4.515985130000	3.532418130000
H	-2.139553040000	2.120971590000	3.188376950000
H	-0.926288560000	7.747017530000	3.650186000000
H	1.620555110000	7.394883360000	3.511774130000
N	-3.766549570000	-4.069670750000	3.348867160000
O	-2.335552450000	0.341880740000	3.073929080000
N	-3.111308360000	-1.827641210000	3.229405750000
C	-4.119602550000	-2.769201030000	3.344231160000
N	-5.414992660000	-2.447192110000	3.436555680000
C	-5.630984410000	-1.122398570000	3.424683020000

C	-4.686666440000	-0.089400000000	3.319056770000
C	-3.312370490000	-0.432438580000	3.201323150000
N	-6.851681060000	-0.475390730000	3.504045910000
C	-6.600269610000	0.890586770000	3.456055740000
N	-5.314089680000	1.153830450000	3.340620220000
H	-2.793620680000	-4.422011580000	3.341854050000
H	-4.515985130000	-4.722597540000	3.532418130000
H	-2.120971590000	-2.139553040000	3.188376950000
H	-7.747017530000	-0.926288560000	3.650186000000
H	-7.394883360000	1.620555110000	3.511774130000
N	4.069670750000	-3.766549570000	3.348867160000
O	-0.341880740000	-2.335552450000	3.073929080000
N	1.827641210000	-3.111308360000	3.229405750000
C	2.769201030000	-4.119602550000	3.344231160000
N	2.447192110000	-5.414992660000	3.436555680000
C	1.122398570000	-5.630984410000	3.424683020000
C	0.089400000000	-4.686666440000	3.319056770000
C	0.432438580000	-3.312370490000	3.201323150000
N	0.475390730000	-6.851681060000	3.504045910000
C	-0.890586770000	-6.600269610000	3.456055740000
N	-1.153830450000	-5.314089680000	3.340620220000
H	4.422011580000	-2.793620680000	3.341854050000
H	4.722597540000	-4.515985130000	3.532418130000
H	2.139553040000	-2.120971590000	3.188376950000
H	0.926288560000	-7.747017530000	3.650186000000
H	-1.620555110000	-7.394883360000	3.511774130000
N	5.575802160000	0.138181280000	-0.000180650000
O	1.363522310000	-1.802044480000	-0.000307990000
N	3.490545610000	-0.908468670000	-0.000604940000
C	4.872084080000	-1.006501450000	-0.000586680000
N	5.520144050000	-2.177927210000	-0.000806800000
C	4.696477800000	-3.235222700000	-0.000240610000
C	3.290851310000	-3.243229060000	0.000262590000
C	2.607157480000	-2.000869530000	-0.000143490000
N	5.054955640000	-4.571403500000	-0.000194010000
C	3.883185740000	-5.316493540000	0.000286060000
N	2.810848040000	-4.551825290000	0.000528680000
H	5.151842130000	1.084539790000	0.000285370000
H	6.580754340000	0.039053560000	0.000012400000
H	3.031344290000	0.022733210000	-0.000750330000
H	6.003276620000	-4.926250990000	-0.000735330000
H	3.891636600000	-6.396504560000	0.000370880000
N	-0.138181280000	5.575802160000	-0.000180650000
O	1.802044480000	1.363522310000	-0.000307990000
N	0.908468670000	3.490545610000	-0.000604940000
C	1.006501450000	4.872084080000	-0.000586680000
N	2.177927210000	5.520144050000	-0.000806800000
C	3.235222700000	4.696477800000	-0.000240610000
C	3.243229060000	3.290851310000	0.000262590000
C	2.000869530000	2.607157480000	-0.000143490000
N	4.571403500000	5.054955640000	-0.000194010000
C	5.316493540000	3.883185740000	0.000286060000
N	4.551825290000	2.810848040000	0.000528680000
H	-1.084539790000	5.151842130000	0.000285370000
H	-0.039053560000	6.580754340000	0.000012400000
H	-0.022733210000	3.031344290000	-0.000750330000
H	4.926250990000	6.003276620000	-0.000735330000
H	6.396504560000	3.891636600000	0.000370880000
N	-5.575802160000	-0.138181280000	-0.000180650000

O	-1.363522310000	1.802044480000	-0.000307990000
N	-3.490545610000	0.908468670000	-0.000604940000
C	-4.872084080000	1.006501450000	-0.000586680000
N	-5.520144050000	2.177927210000	-0.000806800000
C	-4.696477800000	3.235222700000	-0.000240610000
C	-3.290851310000	3.243229060000	0.000262590000
C	-2.607157480000	2.000869530000	-0.000143490000
N	-5.054955640000	4.571403500000	-0.000194010000
C	-3.883185740000	5.316493540000	0.000286060000
N	-2.810848040000	4.551825290000	0.000528680000
H	-5.151842130000	-1.084539790000	0.000285370000
H	-6.580754340000	-0.039053560000	0.000012400000
H	-3.031344290000	-0.022733210000	-0.000750330000
H	-6.003276620000	4.926250990000	-0.000735330000
H	-3.891636600000	6.396504560000	0.000370880000
N	0.138181280000	-5.575802160000	-0.000180650000
O	-1.802044480000	-1.363522310000	-0.000307990000
N	-0.908468670000	-3.490545610000	-0.000604940000
C	-1.006501450000	-4.872084080000	-0.000586680000
N	-2.177927210000	-5.520144050000	-0.000806800000
C	-3.235222700000	-4.696477800000	-0.000240610000
C	-3.243229060000	-3.290851310000	0.000262590000
C	-2.000869530000	-2.607157480000	-0.000143490000
N	-4.571403500000	-5.054955640000	-0.000194010000
C	-5.316493540000	-3.883185740000	0.000286060000
N	-4.551825290000	-2.810848040000	0.000528680000
H	1.084539790000	-5.151842130000	0.000285370000
H	0.039053560000	-6.580754340000	0.000012400000
H	0.022733210000	-3.031344290000	-0.000750330000
H	-4.926250990000	-6.003276620000	-0.000735330000
H	-6.396504560000	-3.891636600000	0.000370880000
K	0.000000000000	0.000000000000	-1.776873480000
K	0.000000000000	0.000000000000	1.776762780000
